# Synthesis of Functionalized Azepines via Cu(I)-Catalyzed Tandem Amination/Cyclization Reaction of Fluorinated Allenynes

**DOI:** 10.3390/molecules27165195

**Published:** 2022-08-15

**Authors:** Anna N. Philippova, Daria V. Vorobyeva, Pavel S. Gribanov, Ivan A. Godovikov, Sergey N. Osipov

**Affiliations:** A. N. Nesmeyanov Institute of Organoelement Compounds, Russian Academy of Sciences, 28 Vavilova str., Moscow 119991, Russia

**Keywords:** azepines, cyclic amino acids, amination, cyclization, catalysis

## Abstract

An efficient method for the selective preparation of trifluoromethyl-substituted azepin-2-carboxylates and their phosphorous analogues has been developed via Cu(I)-catalyzed tandem amination/cyclization reaction of functionalized allenynes with primary and secondary amines.

## 1. Introduction

**Figure 1:**
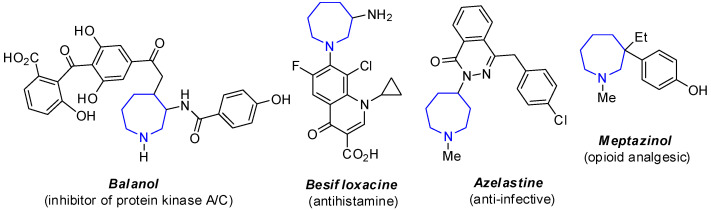
Bioactive molecules containing azepane rings.

Azepane and its functionalized derivatives are important structural motifs present in variety of natural products and bioactive molecules with a wide range of medicinal and pharmaceutical properties [1,2,3,4,5,6,7,8] including antidiabetic [9,10,11], anticancer [12,13,14], and antiviral [15,16,17] activities, thus attracting a significant interest of the researchers in a new lead compound discovery. The most cited example of a natural product containing an azepane core is the fungal metabolite protein kinase inhibitor *Balanol* [18]. Additional examples of bioactive synthetic azepane-based compounds are depicted on Figure 1. Despite remarkable efforts being made to develop efficient synthetic methods for these seven-membered azacycles [19,20,21,22,23,24,25,26,27,28,29,30,31], slow cyclization kinetics have hindered the development of robust methods for the direct construction of these medium-ring heterocyclic systems [32]. Therefore, the development of new effective strategies for the selective preparation of azepane derivatives with unique substitution patterns is of great interest.

On the other hand, organofluorine compounds are currently finding increasingly important applications in modern pharma, crop protection, and materials science [33,34,35,36,37,38]. This fact stimulates synthetic chemists to develop new efficient methodologies for the selective introduction of fluorine and fluorinated groups into different organic molecules. In this context, fluorine-containing amino acids, especially their constrained cyclic derivatives, attract a considerable interest as crucial targets in bioorganic and medicinal chemistry for the design of potent and highly selective bioactive compounds [38,39,40,41].

**Scheme 1:**
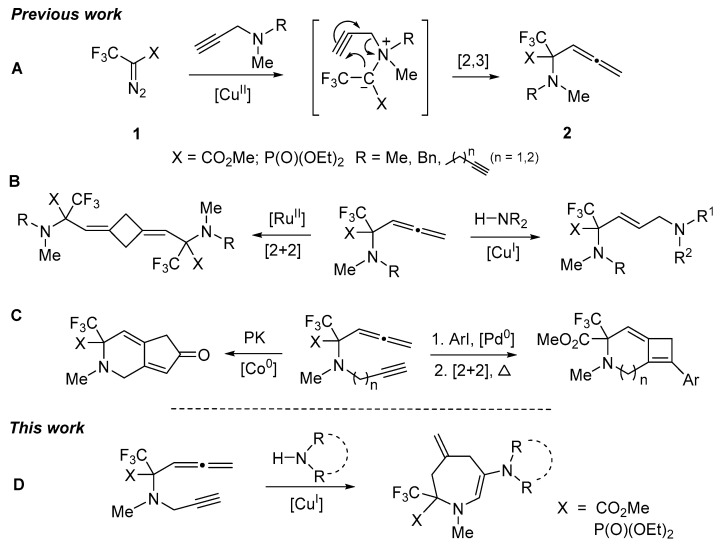
Previous and present work.

Recently, we have developed a convenient one-step protocol for the preparation of functionalized allenes based on [2,3]-sigmatropic rearrangement of propargyl-containing nitrogen ylides generated in situ from α-CF_3_-diazo compounds **1** (Scheme 1A) [42]. The synthetic potential of **2** has been clearly revealed in their intermolecular transformation under transition metal catalysis such as Cu(I)-catalyzed hydroamination [43] and Ru(II)-catalyzed dimerization (Scheme 1B) [44]. In addition, allenynes **2** [R = −(CH_2_)_n_C≡CH] have proved to be unique doubly unsaturated synthons to afford the bicyclic amino acid derivatives via intramolecular Pauson–Khand [42] and [2+2]-cycloaddition [45,46] reactions (Scheme 1C).

According to our long-term program on the study of transition metal-catalyzed reactions of unsaturated α-amino acid derivatives [47,48,49,50,51,52,53,54], now we want to disclose an efficient approach to novel α-CF_3_-containing azepine-2-carboxylates and their phosphorous analogues via a new type of catalytic allenyne transformation involved the combination of intermolecular amine addition with intramolecular cyclization (Scheme 1D). To the best of our knowledge, this reaction constitutes the first example of tandem amination/cyclization of allenynes under metal-catalysis.

## 2. Results and Discussion

Given our recent finding that an allene system is readily capable of undergoing the selective hydroamination with secondary and primary amines in the presence of copper catalysts [41], we were curious to investigate what will happen with allenyne bearing propargyl group with acidic proton on terminal triple bond under the similar catalytic conditions. To answer this question, we began our study by testing the reaction between allenyne **2a** and aniline. Cationic Cu(I) complex Cu(MeCN)_4_PF_6_ was chosen as the most competent catalyst for allene hydroamination process. Anhydrous THF, toluene, 1,2-dichloroethane (DCE), and 1,4-dioxane were employed as the solvents. As a result, the reaction was found to smoothly proceed in the presence of 10 mol% Cu(MeCN)_4_PF_6_ and 2.0 equiv. of aniline in dioxane at 90 °C for 8 h to give an unusual azepine derivative **3a** in 65% NMR yield (Table 1, entry 1).

A prolonged reaction did not lead to a better yield of the product, while causing the formation of a small number of impurities (measured by ^19^F NMR spectroscopy). The subsequent decrease of the catalyst loading to 5 mol% resulted in a notably lower conversion of **2a** affording **3a** in 35% yield (entry 3) along with significant amounts of starting materials. At the same time, the decline of amine amount and reaction temperature have improved the yield of the desired product **3a** (entry 4). Copper(I) salts (CuCl or CuI) have proved to be inactive for the process (entries 8 and 9). Finally, the optimum conditions include the heating of a mixture of allenyne **2a** and 1.2 equiv. aniline in the presence of 10 mol% of catalyst in dioxane at 70 °C for 6 h (entry 10).

**Scheme 2:**
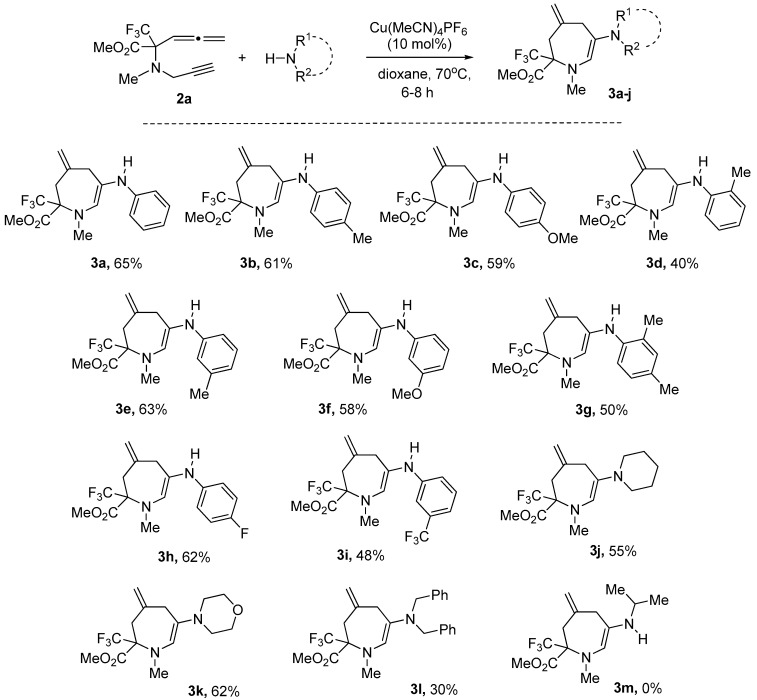
Synthesis of trifluoromethylated azepine-2-carboxylates **3**.

With the optimized conditions in hand, we examined a series of primary and secondary amines (such as substituted anilines, morpholine, and piperidine) as substrates for this catalytic transformation. As a result, we found that the reaction proceeded smoothly with all tested substrates furnishing the corresponding CF_3_-containing azepine-2-carboxylate derivatives **3a**–**j** in moderate to good yields (Scheme 2). However, the only limitation was found for primary aliphatic amines, such as *iso*-propyl and butyl amine; all our attempts to initiate their reactions with allenyne **2a** failed.

**Figure 2:**
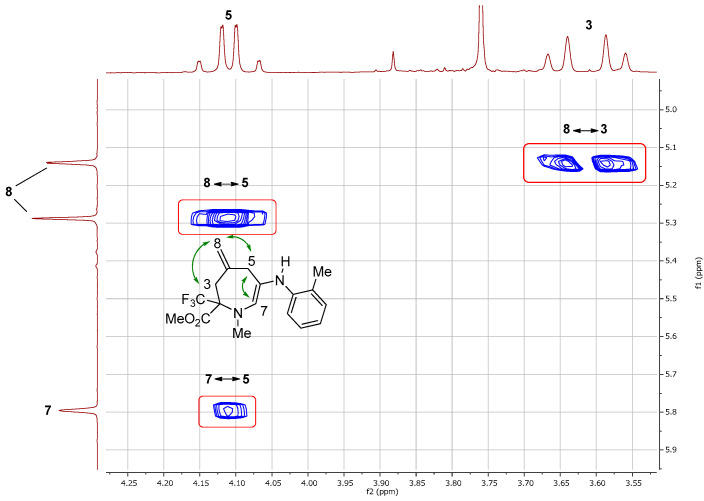
A fragment of 2D ^1^H ROESY NMR spectrum (500 MHz, CDCl_3_) of **3d**.

The characterization of the compounds obtained was performed using standard physicochemical methods (NMR spectroscopy and high-resolution mass spectrometry). The location of exo- and endocyclic double bonds inherent to the seven-membered azepine structure **3** was determined using 2D NMR spectroscopy. Thus, characteristic cross-peaks are observed in the spectrum between protons of the terminal =CH_2_ group at position 8 and protons of neighboring CH_2_ groups at positions 3 and 5 (both are spin AB-systems), unambiguously indicating their spatial proximity (Figure 2).

**Scheme 3:**
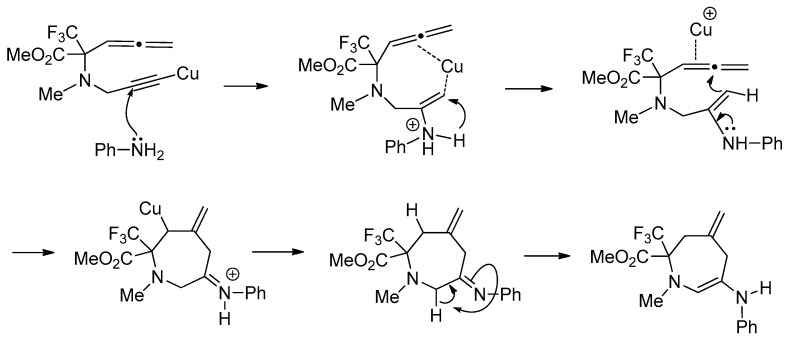
Possible reaction pathway to functional azepines.

A feasible mechanism of this tandem transformation may involve the initial formation of copper acetylide as a key step, which is similar to the well-established Cu(I)-catalyzed click reaction [55,56], due to higher acidity of acetylene proton. Then, apparently, nucleophilic addition of the amine to acetylide occurs according to its inherent polarity, followed by intramolecular cyclization at the central carbon atom of the pre-activated allene system to afford the seven-membered product after typical skeleton reorganization (Scheme 3). More detailed mechanistic study of this unprecedented reaction is currently in progress.

**Scheme 4:**
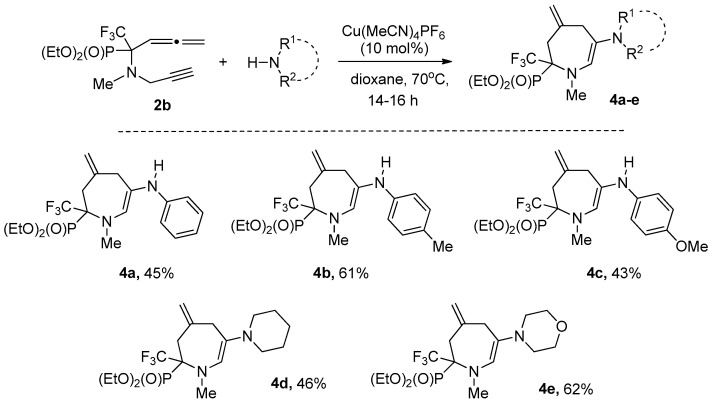
Synthesis of trifluoromethylated azepine-2-phosphonates **4**.

Taking into account that *α*-amino phosphonates are the structural mimics of *α*-amino acids exhibiting a broad spectrum of remarkable biological properties including antibacterial, antiviral, anticancer, and some other types of bioactivity [57,58,59,60,61,62,63], we checked the reactivity of phosphonate-containing allenyne **2b** in the amination/cyclization reaction with amines under the found catalytic conditions. It turned out that **2b** has demonstrated comparable to carboxylate analogue **2a** reactivity towards primary and secondary amines yielding the corresponding trifluoromethylated azepine-2-phosphonates **4a**–**e** in moderate to good yields (Scheme 4).

In general, the NMR yields of **4** exceeded 80% (determined by ^19^F NMR spectroscopy) in all studied reactions; however, moderate yields in some cases were caused by purification to obtain analytically pure samples using column chromatography followed by re-crystallization.

## 3. Materials and Methods

### 3.1. General Information

All solvents used in the reactions were freshly distilled from appropriate drying agents before use. All reagents were used as purchased from Sigma-Aldrich (Munich, Germany). Analytical TLC was performed with Merck silica gel 60 F_254_ plates (Darmstadt, Germany); visualization was accomplished with UV light, iodine vapors, or by spraying with Ce(SO_4_)_2_ solution in 5% H_2_SO_4_. Chromatography was carried out using Merck silica gel (Kieselgel 60, 0.063–0.200 mm, Darmstadt, Germany) and petroleum ether/ethyl acetate, ethyl acetate, ethyl acetate/methanol as an eluent. NMR spectra were obtained with Bruker AV-300 (^19^F, ^31^P) and AV-400 (^1^H, ^13^C, ^19^F, ^31^P) spectrometers (Karlsruhe, Germany) operating at 400 MHz for ^1^H (TMS reference), at 101 MHz for ^13^C, 282 and at 376 MHz for ^19^F (CCl_3_F reference), and at 121 MHz for ^31^P (H_3_PO_4_ reference). High-Resolution Mass Spectrometry spectra were carried out using AB Sciex TripleTOF 5600+ (Framingham, MA, USA) supported different ionization sources. The starting allenynes **2a**,**b** were synthesized via the previously described protocol [42].

### 3.2. General Procedure

A mixture of amine (0.485 mmol), allenyne (0.404 mmol), and [Cu(CH_3_CN)_4_PF_6_] (10 mol%) in anhydrous 1,4-dioxane (3 mL) was stirred under argon at 70 °C for 6–16 h. Then, the reaction mixture was cooled to room temperature, solvent was removed under reduced pressure, and the residue was subjected to column chromatography on silica gel (petroleum ether/ethyl acetate or ethyl acetate/methanol for phosphonate derivatives) to yield the purified product (See Supplementary Materials).

*Methyl 1-methyl-4-methylene-6-(phenylamino)-2-(trifluoromethyl)-2,3,4,7-tetrahydro-1H-azepine-2-carboxylate (* **3a** *)*

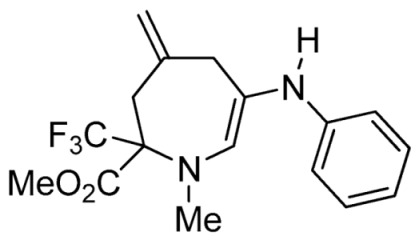

Yield: 65% as a light brown solid. ^1^H NMR (400 MHz, CDCl_3_) δ 7.17 (t, *J* = 7.9 Hz, 2H), 6.72 (t, *J* = 7.3 Hz, 1H), 6.57 (d, *J* = 7.8 Hz, 2H), 5.81 (s, 1H), 5.24 (s, 1H), 5.11 (s, 1H), 4.03 (d, *J* = 3.7 Hz, 2H), 3.93 (s, 1H), 3.74 (s, 3H), 3.63 (d, *J* = 13.7 Hz, 1H), 3.55 (d, *J* = 13.6 Hz, 1H), 2.57 (s, 3H). ^13^C NMR (101 MHz, CDCl_3_) δ 167.4, 147.7, 137.2, 137.1, 129.2, 124.8 (q, *J* = 290.8, 276.5 Hz), 118.9, 117.9, 113.0, 111.0, 70.4 (q, *J* = 25.0 Hz), 55.1, 52.8, 45.2, 40.2. ^19^F NMR (376 MHz, CDCl_3_) δ −68.30. HRMS (ESI) calcd. for C_17_H_20_F_3_N_2_O_2_ [M + H]^+^: 341.1471, found: 341.1472.

*Methyl 1-methyl-4-methylene-6-(p-tolylamino)-2-(trifluoromethyl)-2,3,4,7-tetrahydro-1H-azepine-2-carboxylate (* **3b** *)*

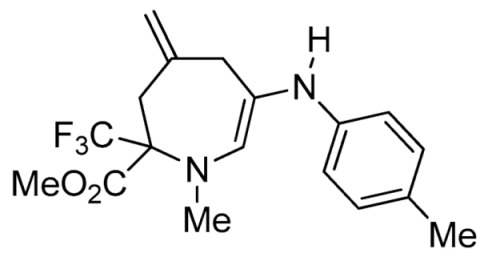

Yield: 61% as a light brown solid. ^1^H NMR (400 MHz, CDCl_3_) δ 6.97 (d, *J* = 8.1 Hz, 2H), 6.49 (d, *J* = 8.2 Hz, 2H), 5.81 (s, 1H), 5.23 (s, 1H), 5.10 (s, 1H), 4.06–3.95 (m, 2H), 3.79 (s, 1H), 3.75 (s, 3H), 3.65–3.50 (m, 3H), 2.56 (s, 3H), 2.24 (s, 3H). ^13^C NMR (101 MHz, CDCl_3_) δ 167.5, 145.5, 137.4, 137.2, 129.7, 127.1, 124.9 (q, *J* = 290.7 Hz), 113.1, 111.0, 70.4 (d, *J* = 25.5 Hz), 55.1, 52.8, 45.7, 40.3, 20.5. ^19^F NMR (376 MHz, CDCl_3_) δ −68.33. HRMS (ESI) calcd. for C_18_H_22_F_3_N_2_O_2_ [M + H]^+^: 355.1628, found: 355.1628.

*Methyl 6-(4-methoxyphenylamino)-1-methyl-4-methylene-2-(trifluoromethyl)-2,3,4,7-tetrahydro-1H-azepine-2-carboxylate (* **3c** *)*

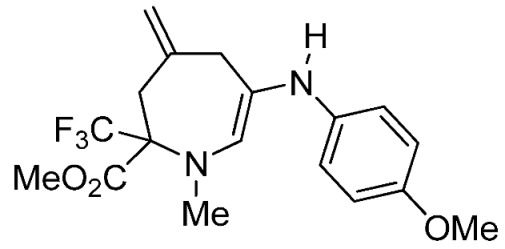

Yield: 59% as a thick brown oil. ^1^H NMR (400 MHz, CDCl_3_) δ 6.75 (d, *J* = 8.3 Hz, 2H), 6.52 (d, *J* = 8.4 Hz, 2H), 5.80 (s, 1H), 5.22 (s, 1H), 5.09 (s, 1H), 4.02–3.92 (m, 2H), 3.74 (s, 6H), 3.61 (d, *J* = 13.6 Hz, 1H), 3.52 (d, *J* = 13.5 Hz, 1H), 2.55 (s, 3H). ^13^C NMR (101 MHz, CDCl_3_) δ 167.4, 152.4, 142.0, 137.4, 137.2, 124.9 (q, *J* = 290.5 Hz), 118.8, 114.8, 114.2, 110.9, 70.4 (q, *J* = 24.9 Hz), 55.8, 55.0, 52.7, 46.1, 40.2. ^19^F NMR (376 MHz, CDCl_3_) δ −68.33. HRMS (ESI) calcd. for C_18_H_22_F_3_N_2_O_3_ [M + H]^+^: 371.1577, found: 371.1579.

*Methyl 1-methyl-4-methylene-6-(o-tolylamino)-2-(trifluoromethyl)-2,3,4,7-tetrahydro-1H-azepine-2-carboxylate (* **3d** *)*

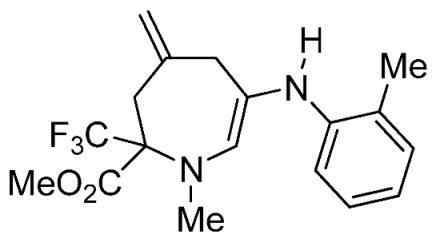

Yield: 40% as a light brown solid. ^1^H NMR (400 MHz, CDCl_3_) δ 7.11–7.04 (m, 2H), 6.70–6.64 (m, 1H), 6.46 (d, *J* = 7.9 Hz, 1H), 5.77 (s, 1H), 5.27 (s, 1H), 5.13 (s, 1H), 4.14–4.04 (m, 2H), 3.82 (s, 1H), 3.74 (s, 3H), 3.64 (d, *J* = 13.7 Hz, 1H), 3.56 (d, *J* = 13.6 Hz, 1H), 2.57 (s, 3H), 2.17 (s, 3H). ^13^C NMR (101 MHz, CDCl_3_) δ 167.4, 145.6, 137.2, 137.2, 130.1, 127.1, 124.9 (q, *J* = 290.9 Hz), 121.9, 118.9, 117.5, 111.0, 110.3, 70.9–69.9 (m), 55.1, 52.8, 45.3, 40.2, 17.6. ^19^F NMR (376 MHz, CDCl_3_) δ −68.32. HRMS (ESI) calcd. for C_18_H_22_F_3_N_2_O_2_ [M + H]^+^: 355.1628, found: 355.1629.

*Methyl 1-methyl-4-methylene-6-(m-tolylamino)-2-(trifluoromethyl)-2,3,4,7-tetrahydro-1H-azepine-2-carboxylate (* **3e** *)*

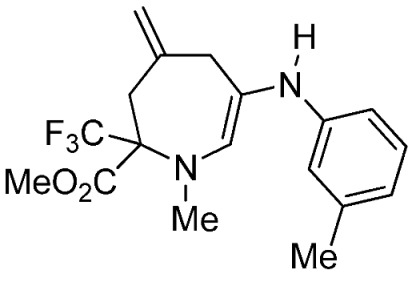

Yield: 63% as a thick light brown oil. ^1^H NMR (400 MHz, CDCl_3_) δ 7.1 (t, *J* = 7.5 Hz, 1H), 6.5 (d, *J* = 7.0 Hz, 1H), 6.4–6.4 (m, 2H), 5.8 (s, 1H), 5.2 (s, 1H), 5.1 (s, 1H), 4.1–4.0 (m, 2H), 3.8 (s, 3H), 3.6 (d, *J* = 13.7 Hz, 1H), 3.5 (d, *J* = 13.6 Hz, 1H), 2.6 (s, 3H), 2.3 (s, 3H). ^13^C NMR (101 MHz, CDCl_3_) δ 167.4, 147.8, 139.0, 137.2, 137.2, 129.1, 124.9 (q, *J* = 290.5 Hz), 118.9–118.9 (m), 118.8, 113.7, 111.0, 110.2, 70.4 (q, *J* = 25.2 Hz), 55.1, 52.7, 45.4, 40.2, 21.7. ^19^F NMR (376 MHz, CDCl_3_) δ −68.4. HRMS (ESI) calcd. for C_18_H_22_F_3_N_2_O_2_ [M + H]^+^: 355.1628, found: 355.1628.

*Methyl 6-(3-methoxyphenylamino)-1-methyl-4-methylene-2-(trifluoromethyl)-2,3,4,7-tetrahydro-1H-azepine-2-carboxylate (* **3f** *)*

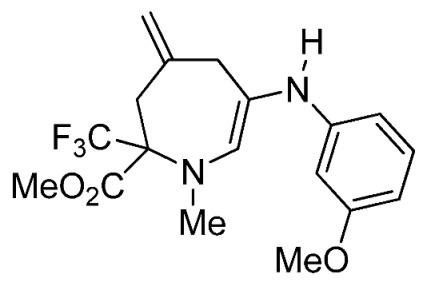

Yield: 58% as a thick brown oil. ^1^H NMR (400 MHz, CDCl_3_) δ 7.06 (t, *J* = 8.1 Hz, 1H), 6.29 (d, *J* = 7.9 Hz, 1H), 6.19 (d, *J* = 8.0 Hz, 1H), 6.11 (s, 1H), 5.80 (s, 1H), 5.22 (s, 1H), 5.10 (s, 1H), 4.08–3.94 (m, 2H), 3.75 (s, 6H), 3.61 (d, *J* = 13.7 Hz, 1H), 3.53 (d, *J* = 13.5 Hz, 1H), 2.56 (s, 3H). ^13^C NMR (101 MHz, CDCl_3_) δ 167.4, 160.9, 149.2, 137.2, 137.1, 130.0, 124.9 (q, *J* = 290.5 Hz), 118.9, 111.0, 106.1, 103.1, 99.0, 70.4 (q, *J* = 25.3 Hz), 55.1, 55.1, 52.7, 45.3, 40.2. ^19^F NMR (376 MHz, CDCl_3_) δ −68.42. HRMS (ESI) calcd. for C_18_H_22_F_3_N_2_O_3_ [M + H]^+^: 371.1577, found: 371.1578.

*Methyl 6-(2,4-dimethylphenylamino)-1-methyl-4-methylene-2-(trifluoromethyl)-2,3,4,7-tetrahydro-1H-azepine-2-carboxylate (* **3g** *)*

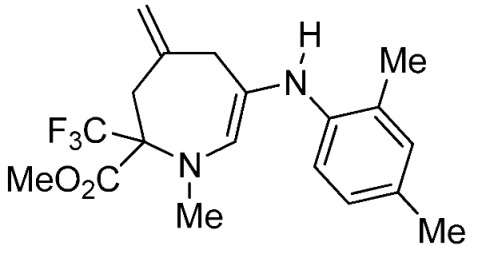

Yield: 50% as a thick light brown oil. ^1^H NMR (400 MHz, CDCl_3_) δ 6.97–6.83 (m, 1H), 6.39 (d, *J* = 7.9 Hz, 1H), 5.79 (s, 1H), 5.27 (s, 1H), 5.12 (s, 1H), 4.13–3.97 (m, 1H), 3.75 (s, 2H), 3.64 (d, *J* = 13.7 Hz, 1H), 3.55 (d, *J* = 13.3 Hz, 1H), 2.57 (s, 2H), 2.23 (s, 2H), 2.15 (s, 2H). ^13^C NMR (101 MHz, CDCl_3_) δ 167.4, 143.4, 137.4, 137.2, 131.0, 127.4, 126.6, 124.9 (q, *J* = 290.7 Hz), 122.1, 118.9, 111.0, 110.5, 70.4 (q, *J* = 25.8 Hz), 55.1, 52.7, 45.7, 40.2, 20.4, 17.5. ^19^F NMR (376 MHz, CDCl_3_) δ −68.32. HRMS (ESI) calcd. for C_19_H_24_F_3_N_2_O_2_ [M + H]^+^: 369.1784, found: 369.1789.

*Methyl 6-(4-fluorophenylamino)-1-methyl-4-methylene-2-(trifluoromethyl)-2,3,4,5-tetrahydro-1H-azepine-2-carboxylate (* **3h** *)*

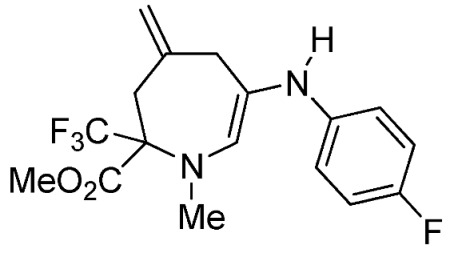

Yield: 62% as a thick brown oil. ^1^H NMR (400 MHz, CDCl_3_) δ 6.87 (t, *J* = 8.7 Hz, 2H), 6.54–6.46 (m, 2H), 5.77 (s, 1H), 5.22 (s, 1H), 5.11 (s, 1H), 4.07–3.92 (m, 2H), 3.74 (s, 3H), 3.64–3.48 (m, 2H), 2.55 (s, 3H). ^13^C NMR (101 MHz, CDCl_3_) δ 167.4, 157.3, 155.0, 143.9, 137.2, 136.9, 124.9 (q, *J* = 290.6 Hz), 119.0, 115.8, 115.6, 113.9, 113.8, 111.0, 70.4 (q, *J* = 25.1, 24.6 Hz), 55.1, 52.8, 45.8, 40.3. ^19^F NMR (376 MHz, CDCl_3_) δ −68.35, −127.72. HRMS (ESI) calcd. for C_17_H_19_F_4_N_2_O_2_ [M + H]^+^: 359.1377, found: 359.1377.

*Methyl 1-methyl-4-methylene-2-(trifluoromethyl)-6-(3-(trifluoromethyl)phenylamino)-2,3,4,5-tetrahydro-1H-azepine-2-carboxylate (* **3i** *)*

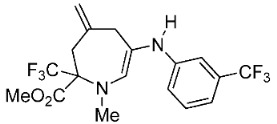

Yield: 48% as a thick brown oil. ^1^H NMR (400 MHz, CDCl_3_) δ 7.25–7.22 (m, 1H), 6.95 (d, *J* = 7.8 Hz, 1H), 6.77–6.68 (m, 2H), 5.76 (s, 1H), 5.23 (s, 1H), 5.13 (s, 1H), 4.14–3.98 (m, 2H), 3.73 (s, 3H), 3.65–3.49 (m, 2H), 2.55 (s, 3H). ^13^C NMR (101 MHz, CDCl_3_) δ 167.4, 147.8, 137.0, 136.4, 129.7, 127.1 (d, *J* = 272.8 Hz), 124.8 (q, *J* = 290.7 Hz), 119.0, 116.2, 114.4 (d, *J* = 3.9 Hz), 111.2, 109.0 (d, *J* = 3.9 Hz), 70.4 (q, *J* = 25.4, 25.0 Hz), 55.1, 52.8, 45.0, 40.3, 29.8. ^19^F NMR (376 MHz, CDCl_3_) δ −62.92, −68.60. HRMS (ESI) calcd. for C_18_H_19_F_6_N_2_O_2_ [M + H]^+^: 409.1345, found: 409.1344.

*Methyl 1-methyl-4-methylene-6-(piperidin-1-yl)-2-(trifluoromethyl)-2,3,4,7-tetrahydro-1H-azepine-2-carboxylate (* **3j** *)*

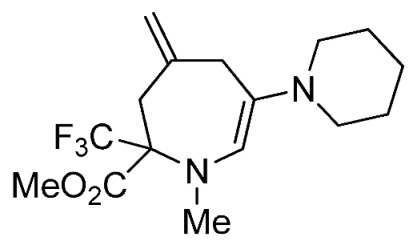

Yield: 55% as a thick brown oil. ^1^H NMR (400 MHz, CDCl_3_) δ 5.78 (s, 1H), 5.42 (s, 1H), 5.04 (s, 1H), 3.81 (s, 3H), 3.57 (d, *J* = 14.1 Hz, 1H), 3.47 (d, *J* = 13.9 Hz, 1H), 3.11 (q, *J* = 9.2 Hz, 2H), 2.54 (s, 3H), 2.33 (s, 4H), 1.57–1.51 (m, 4H), 1.41 (s, 2H). ^13^C NMR (101 MHz, CDCl_3_) δ 167.7, 137.7, 137.4, 125.0 (q, *J* = 290.4 Hz), 119.7, 111.8, 60.3, 55.3, 54.8, 52.7, 40.3, 26.1, 24.5. ^19^F NMR (376 MHz, CDCl_3_) δ −68.73. HRMS (ESI) calcd. for C_16_H_24_F_3_N_2_O_2_ [M + H]^+^: 333.1784, found: 333.1786.

*Methyl 1-methyl-4-methylene-6-morpholino-2-(trifluoromethyl)-2,3,4,7-tetrahydro-1H-azepine-2-carboxylate (* **3k** *)*

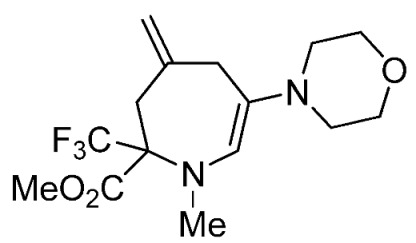

Yield: 62% as a thick brown oil. ^1^H NMR (400 MHz, CDCl_3_) δ 5.77 (s, 1H), 5.44 (s, 1H), 5.06 (s, 1H), 3.80 (s, 3H), 3.69–3.65 (m, 4H), 3.56 (d, *J* = 13.6 Hz, 1H), 3.45 (d, *J* = 13.5 Hz, 1H), 3.13 (q, 2H), 2.52 (s, 3H), 2.42–2.36 (m, 4H). ^13^C NMR (101 MHz, CDCl_3_) δ 167.5, 137.4, 136.7, 129.7–120.5 (m), 120.3, 112.1, 70.4 (q, *J* = 25.2 Hz), 67.1, 60.2, 55.2, 53.7, 52.8, 40.3. ^19^F NMR (376 MHz, CDCl_3_) δ −68.76. HRMS (ESI) calcd. for C_15_H_22_F_3_N_2_O_3_ [M + H]^+^: 335.1577, found: 335.1583.

*Methyl 6-(dibenzylamino)-1-methyl-4-methylene-2-(trifluoromethyl)-2,3,4,7-tetrahydro-1H-azepine-2-carboxylate (* **3l** *)*

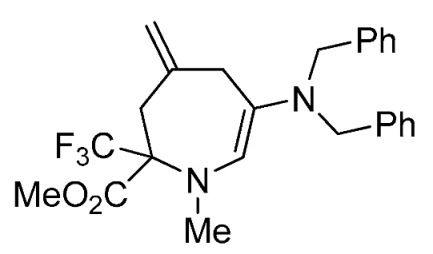

Yield: 30% as thick yellow oil. ^1^H NMR (400 MHz, CDCl_3_) δ 7.35–7.28 (m, 8H), 7.26–7.21 (m, 2H), 6.05 (s, 1H), 5.28 (s, 1H), 5.03 (s, 1H), 3.76 (s, 3H), 3.56–3.49 (m, 5H), 3.45 (d, *J* = 13.5 Hz, 1H), 3.24 (s, 2H), 2.53 (s, 3H). ^13^C NMR (101 MHz, CDCl_3_) δ 167.7, 139.2, 138.2, 137.4, 129.0, 128.4, 127.1, 123.5 (t, *J* = 290.0 Hz), 120.1, 112.1, 70.5 (d, *J* = 25.2 Hz), 58.4, 55.4, 55.2, 52.7, 40.3. ^19^F NMR (376 MHz, CDCl_3_) δ −68.73. HRMS (ESI) calcd. for C_25_H_28_F_3_N_2_O_2_ [M + H]^+^: 445.2097, found: 445.2092.

*Diethyl 1-methyl-4-methylene-6-(phenylamino)-2-(trifluoromethyl)-2,3,4,7-tetrahydro-1H-azepin-2-ylphosphonate (* **4a** *)*

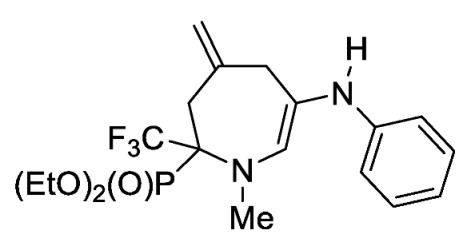

Yield: 45% as a yellow solid. ^1^H NMR (400 MHz, CDCl_3_) δ 7.15 (t, *J* = 7.8 Hz, 2H), 6.70 (t, *J* = 7.3 Hz, 1H), 6.58 (d, *J* = 7.8 Hz, 2H), 5.89 (d, *J* = 5.3 Hz, 1H), 5.19 (s, 1H), 5.04 (s, 1H), 4.13 – 3.99 (m, 6H), 3.48 (s, 2H), 2.85 (s, 3H), 1.24–1.16 (m, 6H). ^13^C NMR (101 MHz, CDCl_3_) δ 147.8, 137.7 (d, *J* = 3.4 Hz), 136.9 (d, *J* = 9.9 Hz), 129.2, 129.7–120.6 (m), 119.1–118.8 (m), 117.8, 113.0, 110.2 (d, *J* = 2.2 Hz), 64.6 (d, *J* = 7.3 Hz), 62.9 (d, *J* = 8.0 Hz), 55.3 (d, *J* = 7.4 Hz), 45.4 (d, *J* = 1.9 Hz), 41.0, 16.5 (d, *J* = 5.9 Hz), 16.3 (d, *J* = 6.0 Hz). ^19^F NMR (376 MHz, CDCl_3_) δ −64.56 (d, *J* = 6.5 Hz). ^31^P NMR (121 MHz, CDCl_3_) δ 14.5 (q, *J* = 6.8 Hz). HRMS (ESI) calcd. for C_19_H_27_F_3_N_2_O_3_P [M + H]^+^: 419.1706, found: 419.1709.

*Diethyl 1-methyl-4-methylene-6-(p-tolylamino)-2-(trifluoromethyl)-2,3,4,7-tetrahydro-1H-azepin-2-ylphosphonate (* **4b** *)*

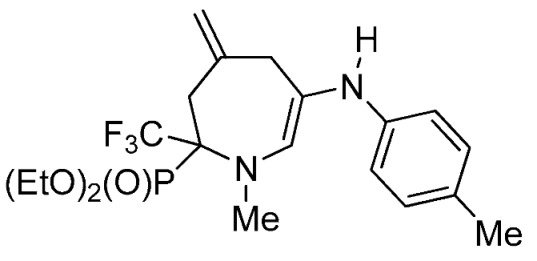

Yield: 61% as a thick brown oil. ^1^H NMR (400 MHz, CDCl_3_) δ 6.97 (d, *J* = 7.9 Hz, 2H), 6.52 (d, *J* = 7.6 Hz, 2H), 5.94–5.84 (m, 1H), 5.20 (s, 1H), 5.04 (s, 1H), 4.14–3.97 (m, 6H), 3.48 (s, 2H), 2.85 (s, 3H), 2.22 (s, 3H), 1.25–1.16 (m, 6H). ^13^C NMR (101 MHz, CDCl_3_) δ 145.5, 137.7 (d, *J* = 3.3 Hz), 137.2 (d, *J* = 9.7 Hz), 129.7, 127.0, 125.2 (dd, *J* = 292.9, 12.6 Hz). 119.0 (d, *J* = 7.7 Hz), 113.2, 110.2, 64.6 (d, *J* = 7.3 Hz), 63.0 (d, *J* = 8.0 Hz), 55.3 (d, *J* = 7.3 Hz), 45.8, 41.0, 20.5, 16.8–15.6 (m). ^19^F NMR (376 MHz, CDCl_3_) δ −64.57 (d, *J* = 6.3 Hz). ^31^P NMR (121 MHz, CDCl_3_) δ 14.5 (q, *J* = 7.2 Hz). HRMS (ESI) calcd. for C_20_H_29_F_3_N_2_O_3_P [M + H]^+^: 433.1862, found: 433.1862.

*Diethyl 6-(4-methoxyphenylamino)-1-methyl-4-methylene-2-(trifluoromethyl)-2,3,4,7-tetrahydro-1H-azepin-2-ylphosphonate (* **4c** *)*

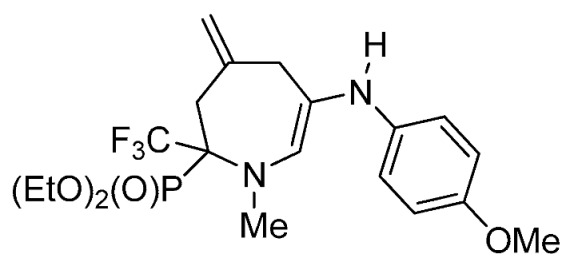

Yield: 43% as a thick brown oil. ^1^H NMR (400 MHz, CDCl_3_) δ 6.75 (d, *J* = 8.8 Hz, 2H), 6.55 (d, *J* = 8.7 Hz, 2H), 5.90 (d, *J* = 5.3 Hz, 1H), 5.19 (s, 1H), 5.03 (s, 1H), 4.15–4.01 (m, 4H), 3.98 (s, 2H), 3.73 (s, 3H), 3.47 (s, 2H), 2.85 (s, 3H), 1.24–1.19 (m, 6H). ^13^C NMR (101 MHz, CDCl_3_) δ 152.4, 142.1, 137.8 (d, *J* = 3.3 Hz), 137.3 (d, *J* = 9.8 Hz), 125.2 (dd, *J* = 293.1, 12.8 Hz), 118.9 (d, *J* = 10.3 Hz), 114.9, 114.3, 110.2, 64.6 (d, *J* = 7.2 Hz), 63.0 (d, *J* = 7.8 Hz), 55.4 (d, *J* = 7.3 Hz), 46.3, 41.0, 29.8, 16.4 (dd, *J* = 21.8, 5.8 Hz). ^19^F NMR (376 MHz, CDCl_3_) δ −64.57 (d, *J* = 6.7 Hz). ^31^P NMR (121 MHz, CDCl_3_) δ 14.6 (q, *J* = 7.1 Hz). HRMS (ESI) calcd. for C_20_H_29_F_3_N_2_O_4_P [M + H]^+^: 449.1812, found: 449.1813.

*Diethyl 1-methyl-4-methylene-6-(piperidin-1-yl)-2-(trifluoromethyl)-2,3,4,7-tetrahydro-1H-azepin-2-ylphosphonate (* **4d** *)*

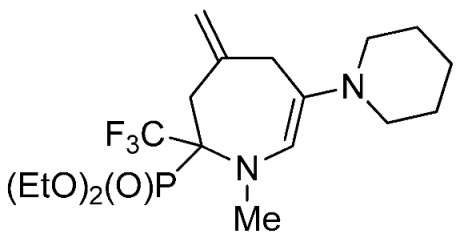

Yield: 46% as a thick brown oil. ^1^H NMR (400 MHz, CDCl_3_) δ 6.00–5.96 (m, 1H), 5.40 (s, 1H), 5.06 (s, 1H), 4.21–4.15 (m, 4H), 3.50–3.36 (m, 4H), 2.83 (s, 3H), 2.67–2.55 (m, 4H), 1.66 (p, *J* = 5.7 Hz, 4H), 1.52–1.44 (m, 2H), 1.33 (t, *J* = 7.1 Hz, 3H), 1.28–1.22 (m, 3H). ^13^C NMR (101 MHz, CDCl_3_) δ 137.7 (d, *J* = 3.7 Hz), 135.1–134.8 (m), 125.2 (d, *J* = 280.8 Hz), 122.7–122.3 (m), 112.0, 64.8 (d, *J* = 7.7 Hz), 63.4 (d, *J* = 7.7 Hz), 59.5, 55.2 (d, *J* = 7.7 Hz), 54.5, 40.9, 25.1, 23.6, 16.5 (dd, *J* = 5.7, 3.5 Hz). ^19^F NMR (282 MHz, C_6_D_6_) δ −63.08 (d, *J* = 7.7 Hz). ^31^P NMR (121 MHz, CDCl_3_) δ 14.84 (q, *J* = 7.3 Hz). HRMS (ESI) calcd. for C_18_H_31_F_3_N_2_O_3_P [M + H]^+^: 411.2019, found: 411.2024.

*Diethyl 1-methyl-4-methylene-6-morpholino-2-(trifluoromethyl)-2,3,4,7-tetrahydro-1H-azepin-2-ylphosphonate (* **4e** *)*

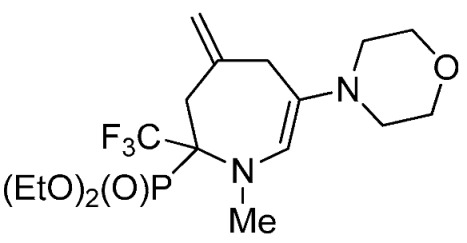

Yield: 62% as a thick brown oil. ^1^H NMR (400 MHz, CDCl_3_) δ 5.91–5.81 (m, 1H), 5.39 (s, 1H), 4.99 (s, 1H), 4.20–4.10 (m, 4H), 3.69–3.65 (m, 4H), 3.45 (d, *J* = 13.6 Hz, 1H), 3.37 (d, *J* = 13.2 Hz, 1H), 3.15 (d, *J* = 2.7 Hz, 2H), 2.82 (s, 3H), 2.41 (s, 4H), 1.31 (t, *J* = 7.1 Hz, 3H), 1.21 (t, *J* = 7.1 Hz, 3H). ^13^C NMR (101 MHz, CDCl_3_) δ 137.9 (d, *J* = 3.4 Hz), 136.2 (d, *J* = 9.9 Hz), 125.3 (qd, *J* = 293.4, 13.2 Hz), 120.7–120.3 (m), 111.2 (d, *J* = 2.1 Hz), 67.1, 64.7 (d, *J* = 7.2 Hz), 63.0 (d, *J* = 7.7 Hz), 60.6–60.5 (m), 55.3 (d, *J* = 8.0 Hz), 53.7, 40.9, 16.5 (dd, *J* = 5.7, 2.0 Hz). ^19^F NMR (376 MHz, CDCl_3_) δ −64.24 (d, *J* = 7.4 Hz). ^31^P NMR (121 MHz, CDCl_3_) δ 14.6. HRMS (ESI) calcd. for C_17_H_29_F_3_N_2_O_4_P [M + H]^+^: 413.1812, found: 413.1819.

## 4. Conclusions

In conclusion, we have elaborated an effective protocol for the preparation of novel CF_3_-containing azepin-2-carboxylate and azepin-2-phosphonate derivatives. The method is based on a new type of tandem transformation of functionalized allenynes under copper(I) catalysis, which includes a combination of intermolecular addition of an amine to a copper-activated triple bond followed by intramolecular cyclization along the allenyl group. The reactions can be readily accomplished in dioxane within a few hours at 70 °C in the presence of complex Cu(MeCN)_4_PF_6_ (10 mol%) providing access to a new family of functionally substituted seven-membered azacycles.

## Figures and Tables

**Table 1:**
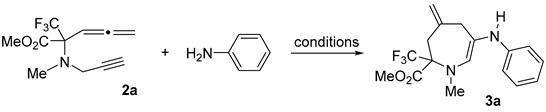
Optimization of amination/cyclization of allenyne **2a** with aniline ^1^.

Entry	Amine (Equiv.)	Catalyst (mol%)	Solv./Temp. (°C)	Time (h)	Yield ^2^ (%)
1	2.0	Cu(MeCN)_4_PF_6_ (10)	dioxane/90	8	65 (43 ^3^)
2	2.0	Cu(MeCN)_4_PF_6_ (10)	dioxane/90	16	60
3	2.0	Cu(MeCN)_4_PF_6_ (5)	dioxane/90	8	35
4	1.5	Cu(MeCN)_4_PF_6_ (10)	dioxane/80	8	77
5	1.5	Cu(MeCN)_4_PF_6_ (10)	DCE/80	16	35
6	1.5	Cu(MeCN)_4_PF_6_ (10)	toluene/80	16	43
7	1.5	Cu(MeCN)_4_PF_6_ (10)	THF/70	16	75
8	2.0	CuI (10)	dioxane/90	8	NR
9	2.0	CuCl (10)	dioxane/90	8	NR
**10**	**1.2**	**Cu(MeCN)_4_PF_6_ (10)**	**dioxane/70**	**6**	**91 (65 ^3^)**
11	2.0	-	dioxane/90	16	NR

^1^*Reagents and conditions*: Allenyne **2a** (0.2 mmol), solvent (3 mL). ^2^ Determined by ^19^F NMR spectroscopy. ^3^ Isolated yield.

## Data Availability

Data are contained within the article and Supplementary Materials.

## Supplementary Materials

The following are available online: https://www.mdpi.com/article/10.3390/molecules27165195/s1, copies of ^1^H, ^13^C NMR and HRMS spectra for all novel compounds.

## Sample Availability

Samples of all of the compounds are available from the authors.
