# Supplementary material for: Synthesis of Functionalized Azepines via Cu(I)-Catalyzed Tandem Amination/Cyclization Reaction of Fluorinated Allenynes
Source: Molecules. 2022 Aug 15;27(16):5195. doi: 10.3390/molecules27165195 (PMC9416787; doi:10.3390/molecules27165195)
Supplement: Supplementary file 1 [file molecules-27-05195-s001.zip › molecules-1852144-supplementary.pdf]

Table of contents

1. ^{1}H, ^{13}C, ^{19}F, ^{31}P NMR and HRMS spectra of **3a-l** and **4a-e**……………………S2-S46

2. 2D NMR spectra of **3a** and **3d**…………………………………………………..S47

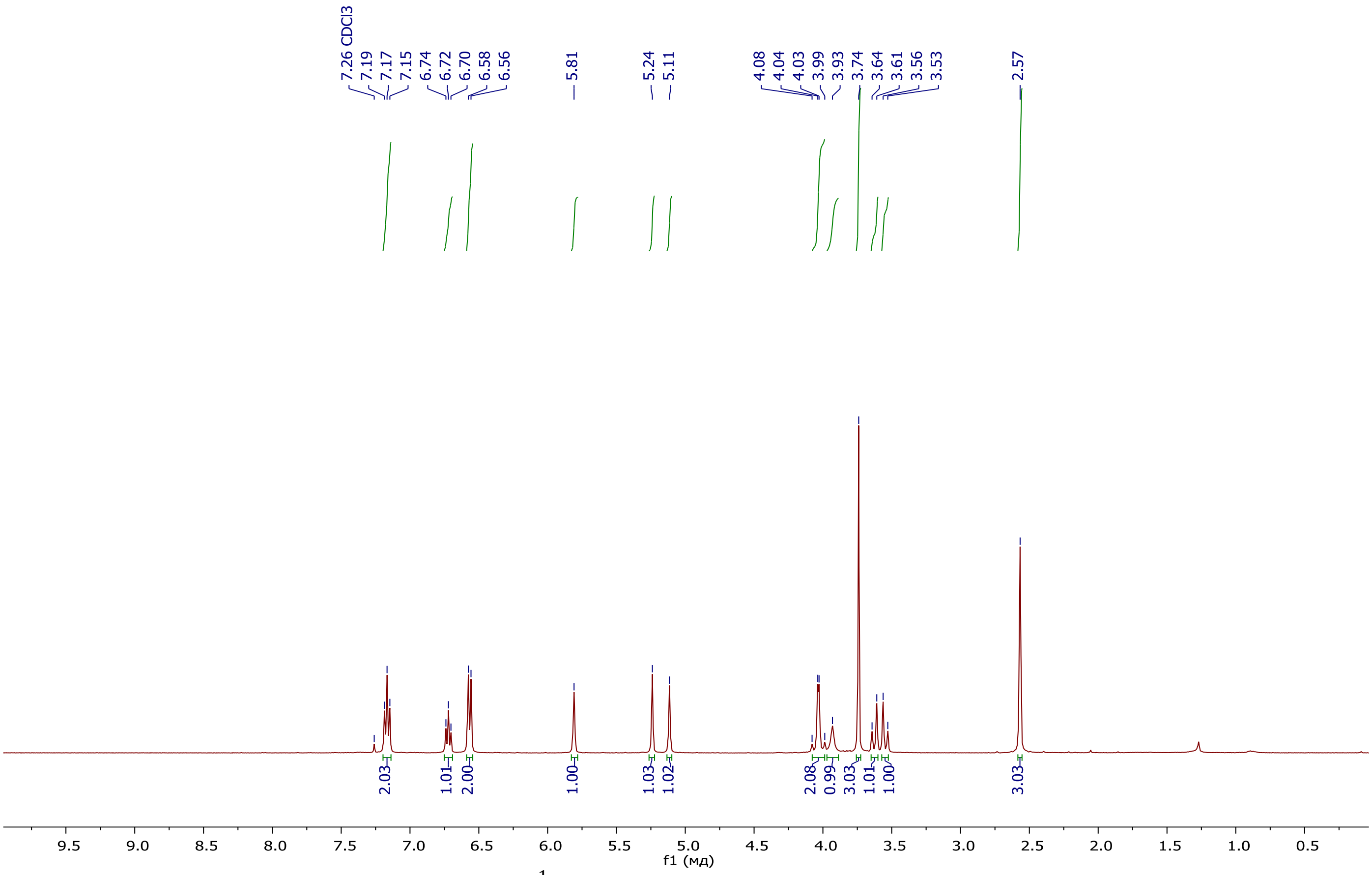

Figure S1. ^{1}H NMR spectrum of **3a** in $CDCl_3$

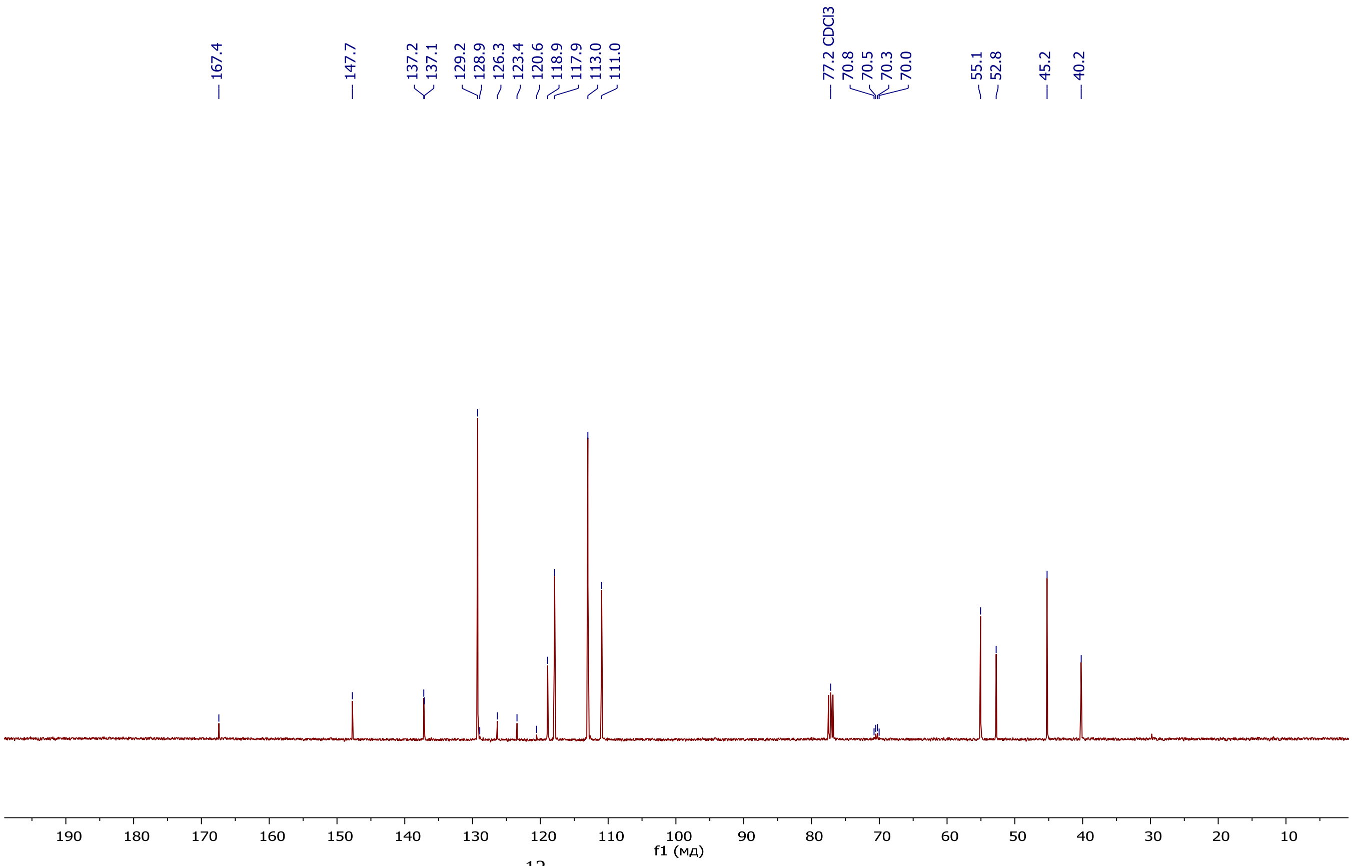

Figure S2. ^{13}C NMR spectrum of **3a** in $CDCl_3$

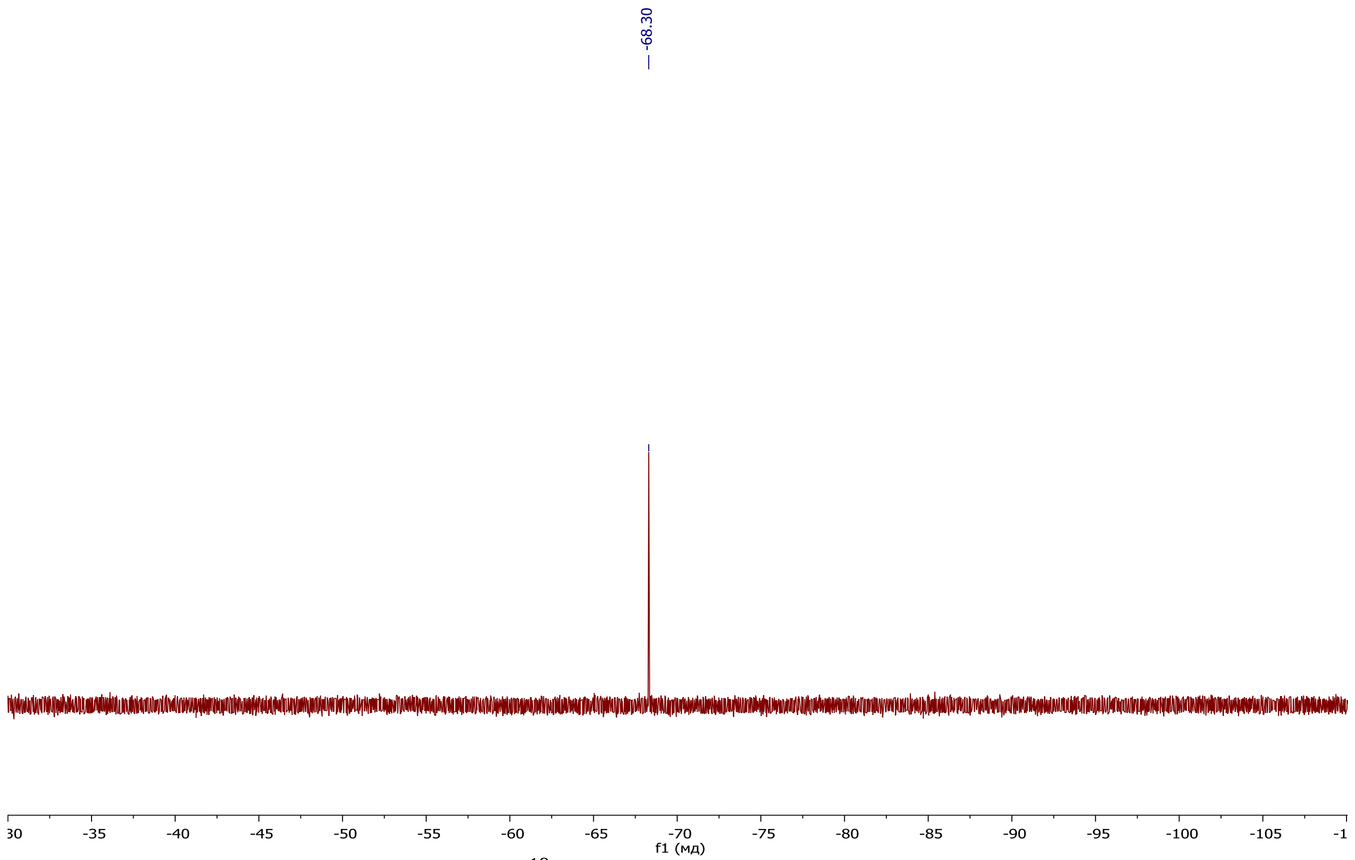

Figure S3. ^{19}F NMR spectrum of **3a** in $CDCl_3$

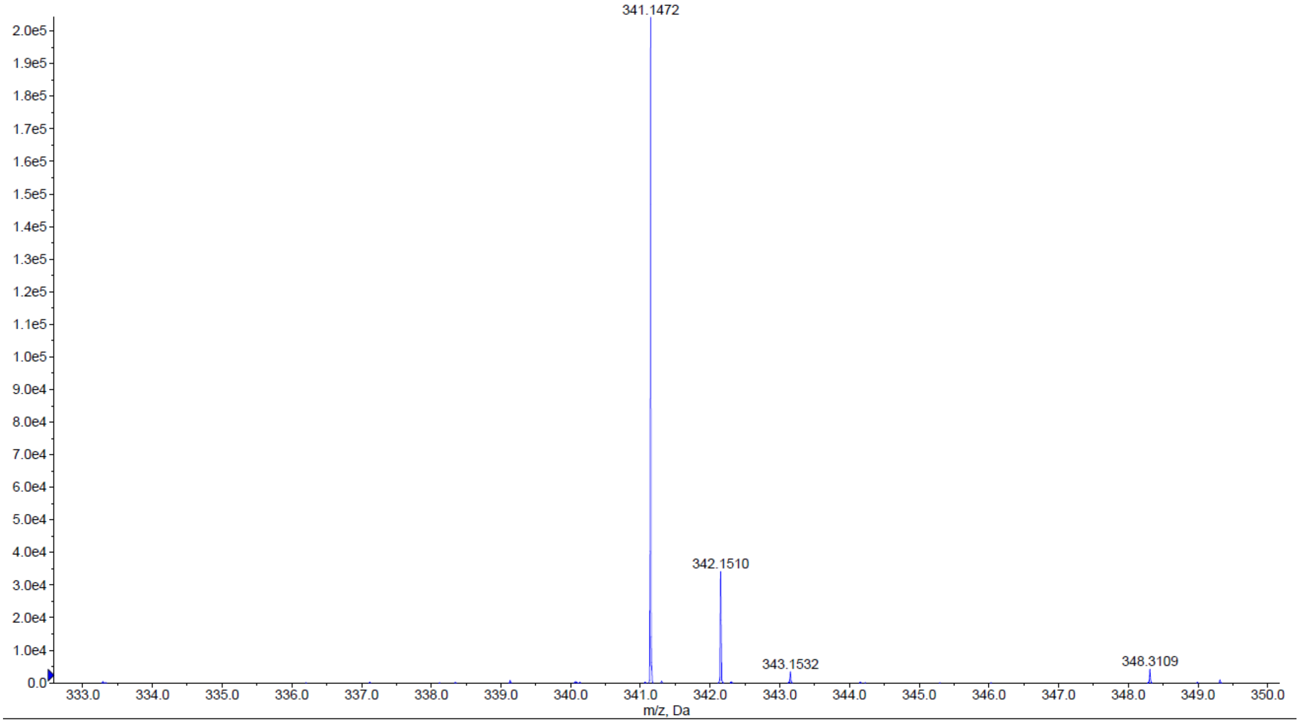

Figure S4. HRMS of **3a**

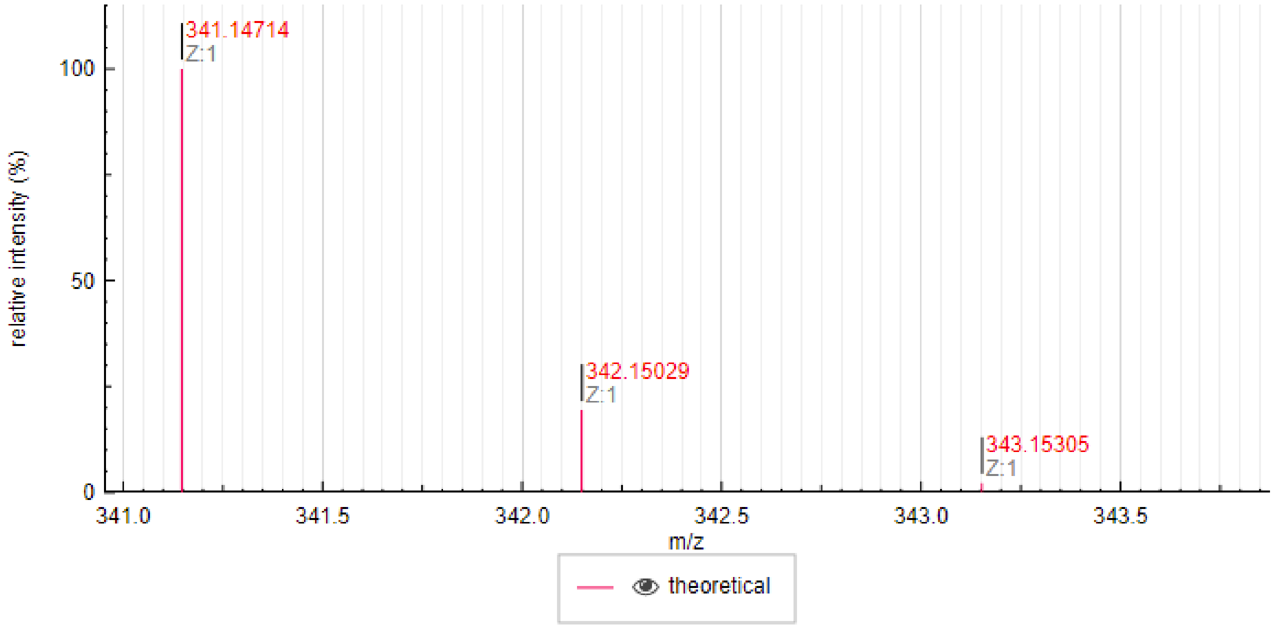

Figure S5. Theoretical HRMS $[M+H]^+$ of **3a**

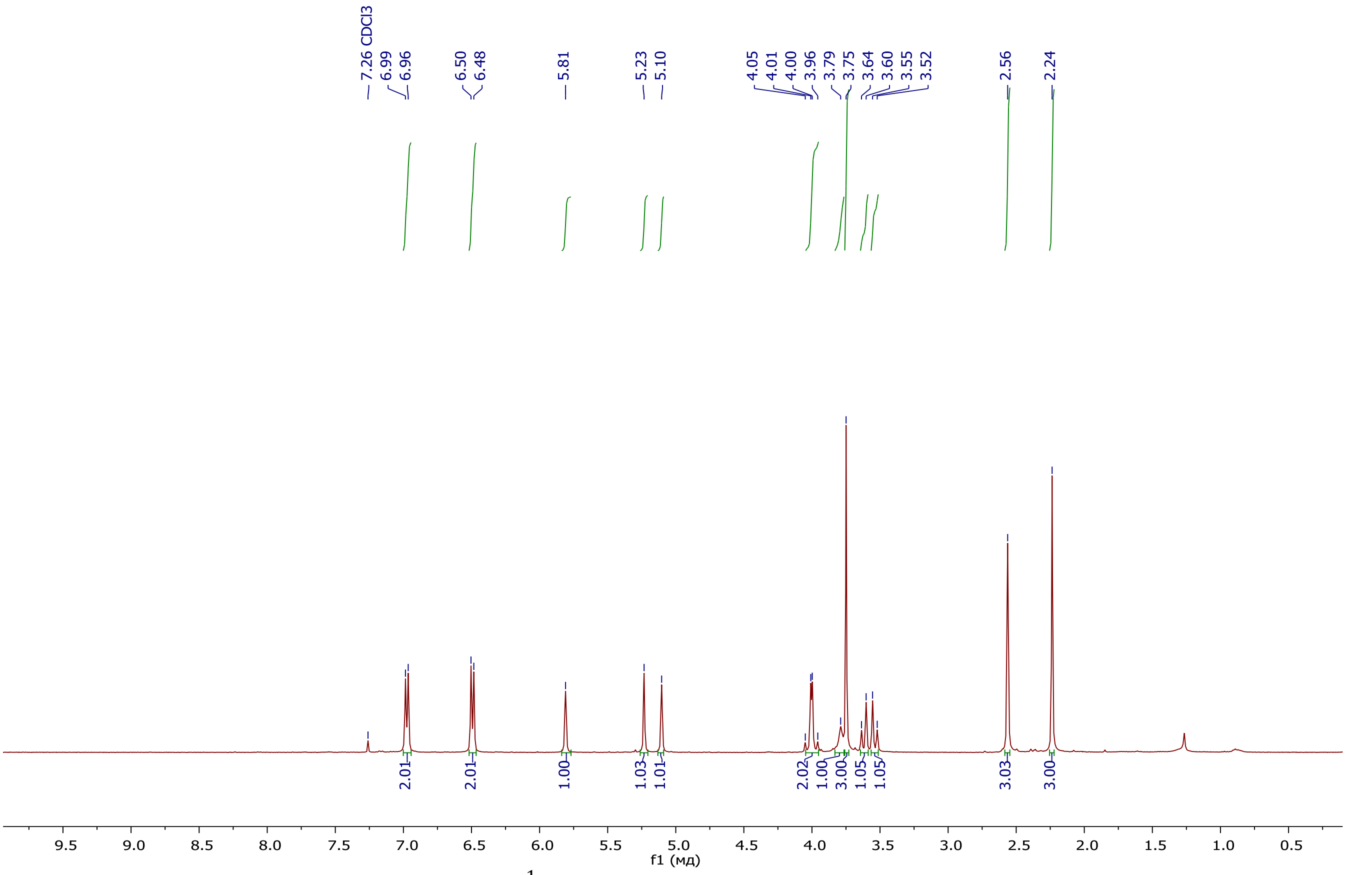

Figure S6. ^{1}H NMR spectrum of **3b** in $CDCl_3$

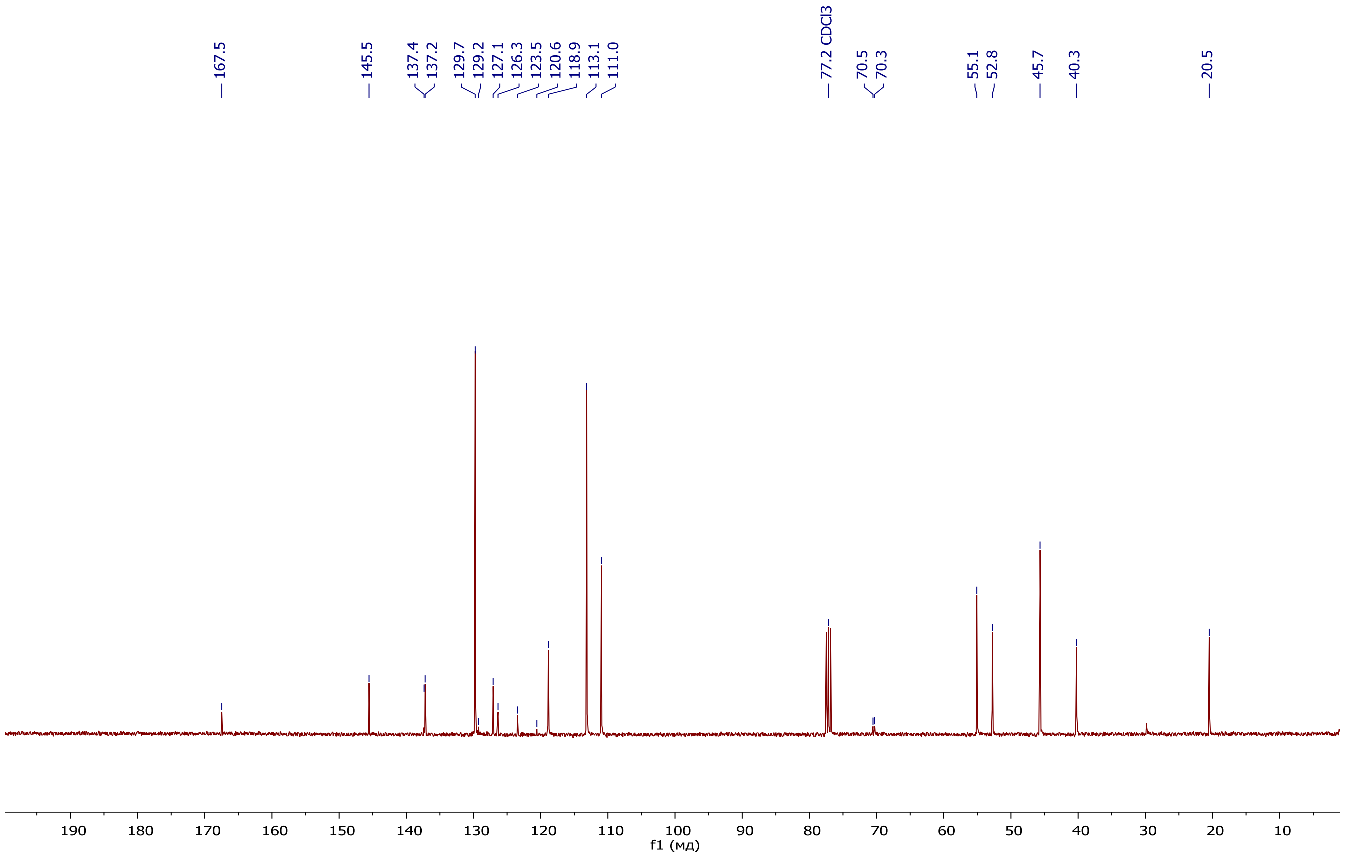

Figure S7. ^{13}C NMR of **3b** in $CDCl_3$

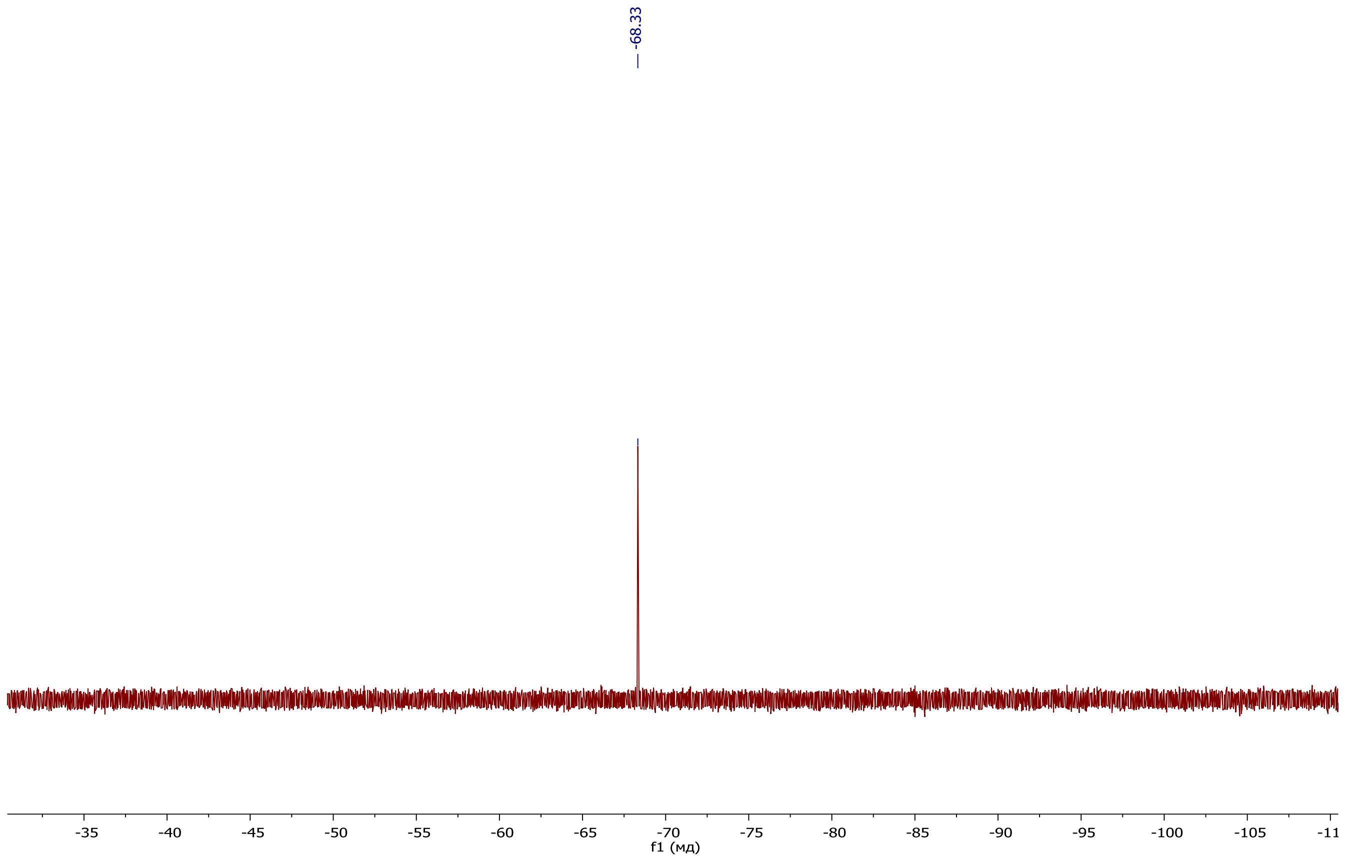

Figure S8. ^{19}F NMR of **3b** in $CDCl_3$

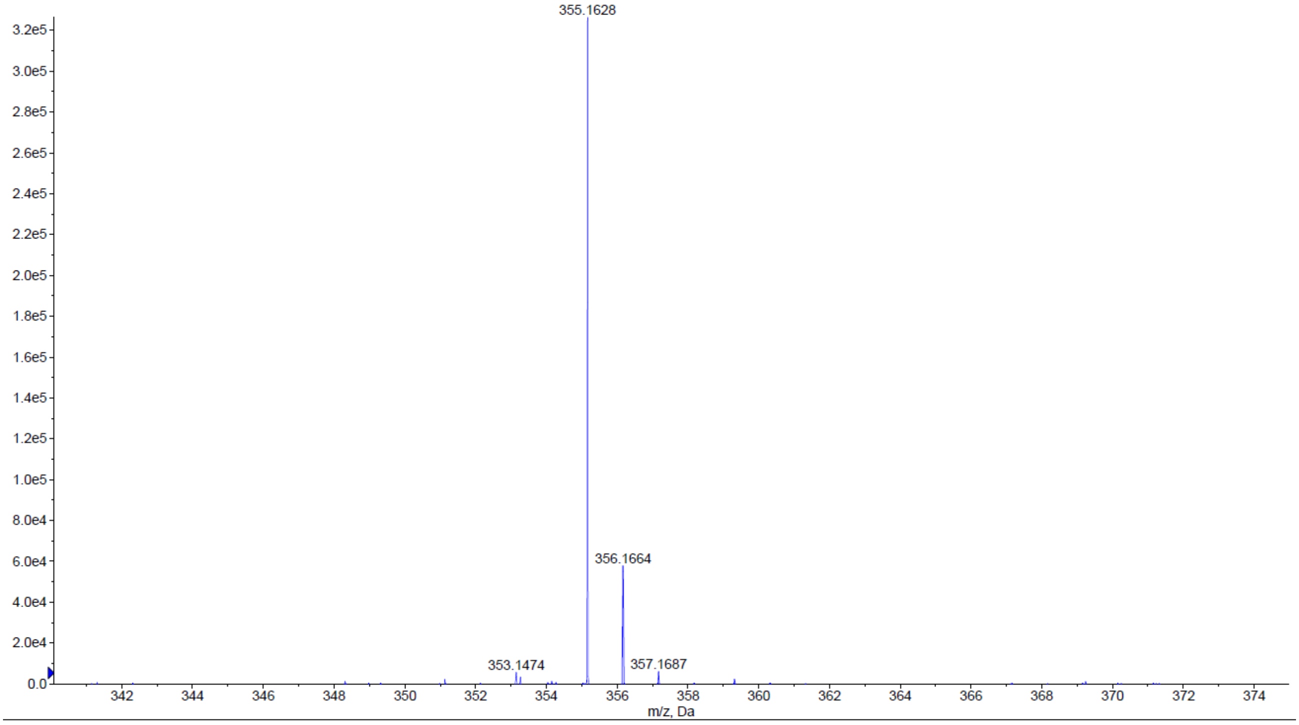

Figure S9. HRMS of **3b**

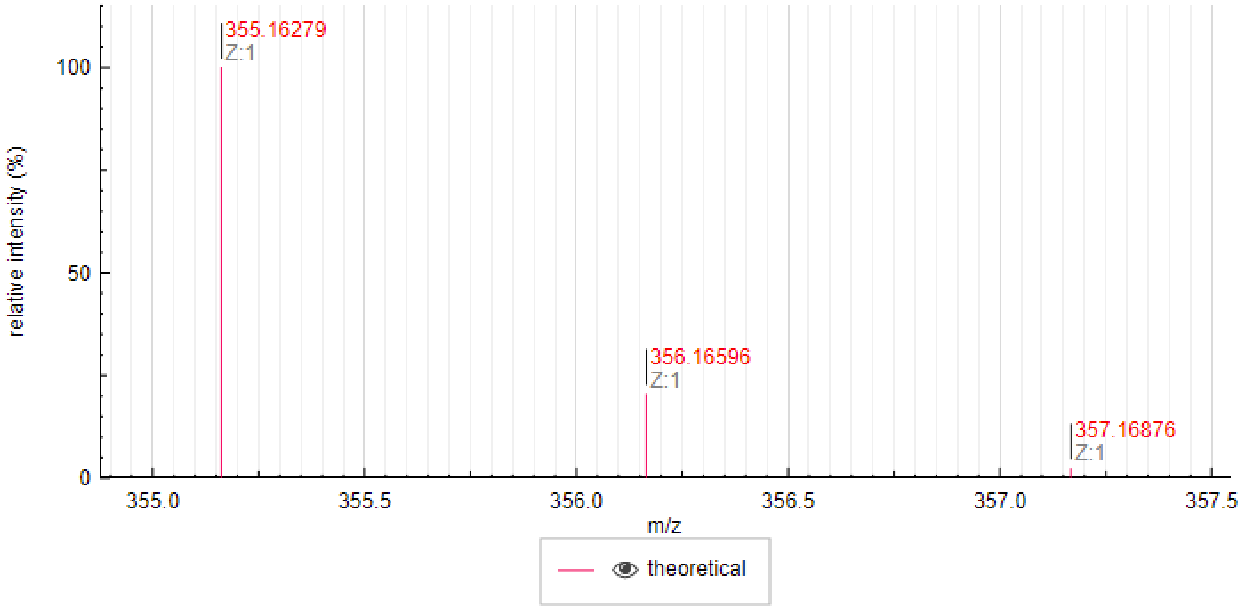

Figure S10. Theoretical HRMS $[M+H]^+$ of **3b**

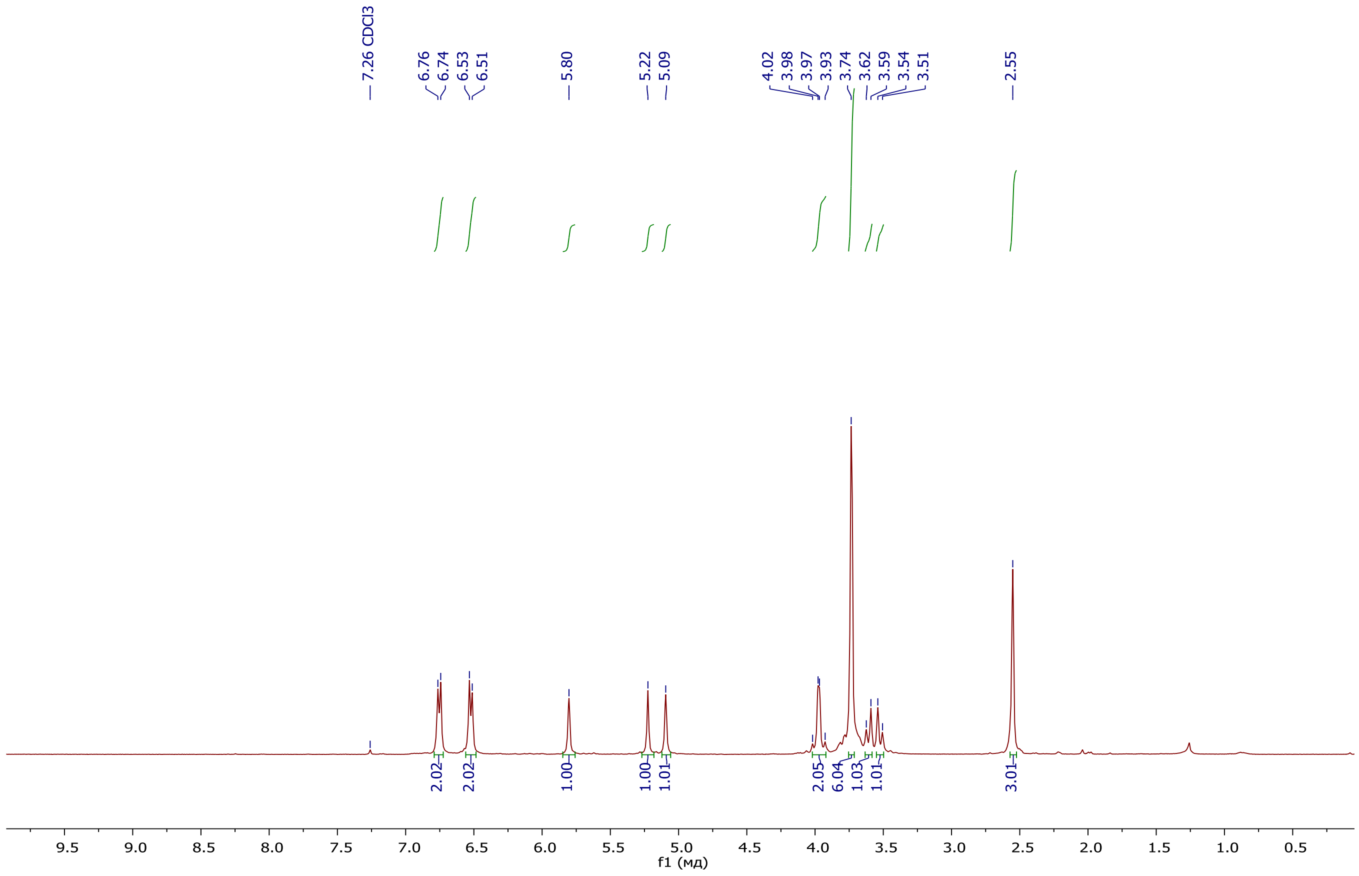

Figure S11. ^{1}H NMR of **3c** in $CDCl_3$

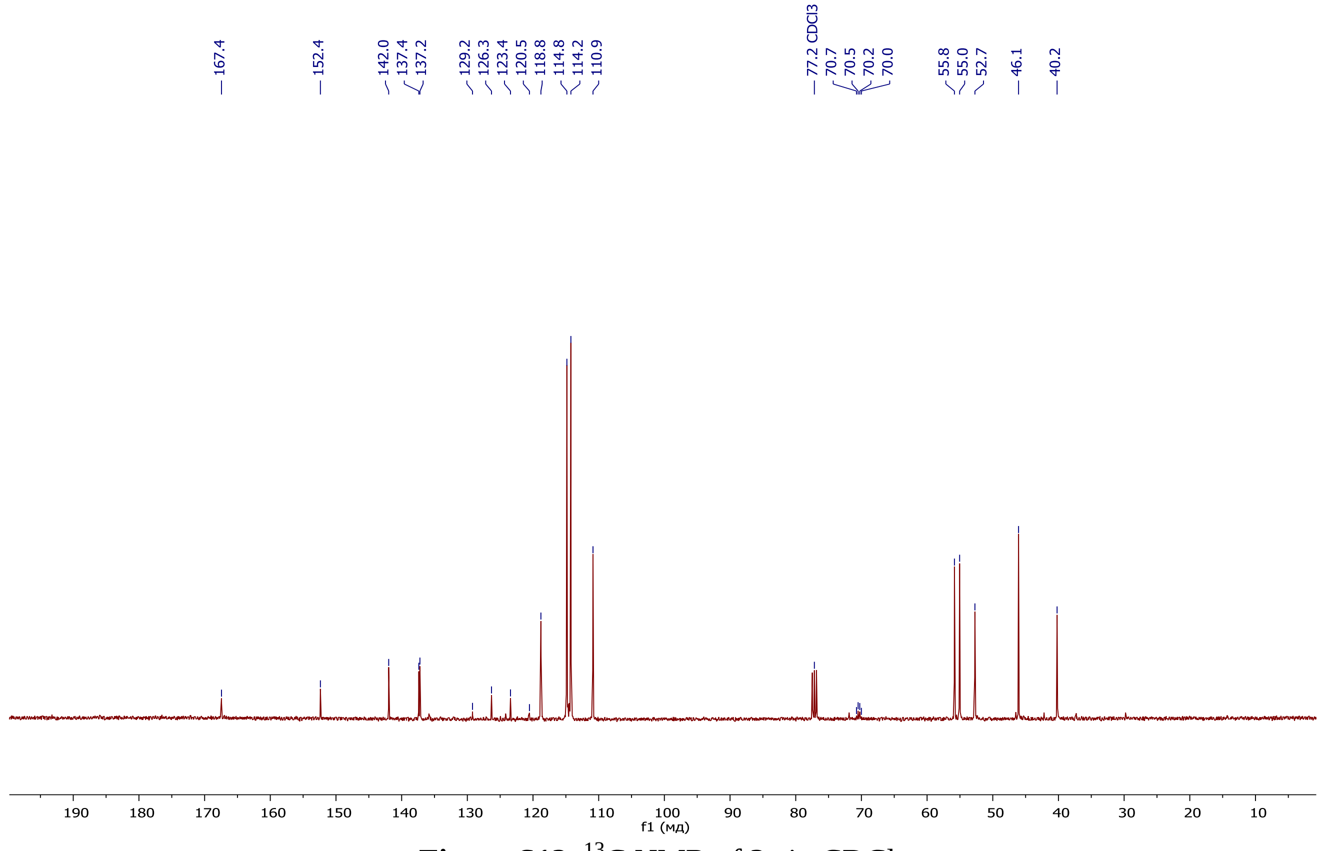

Figure S12. ^{13}C NMR of **3c** in $CDCl_3$

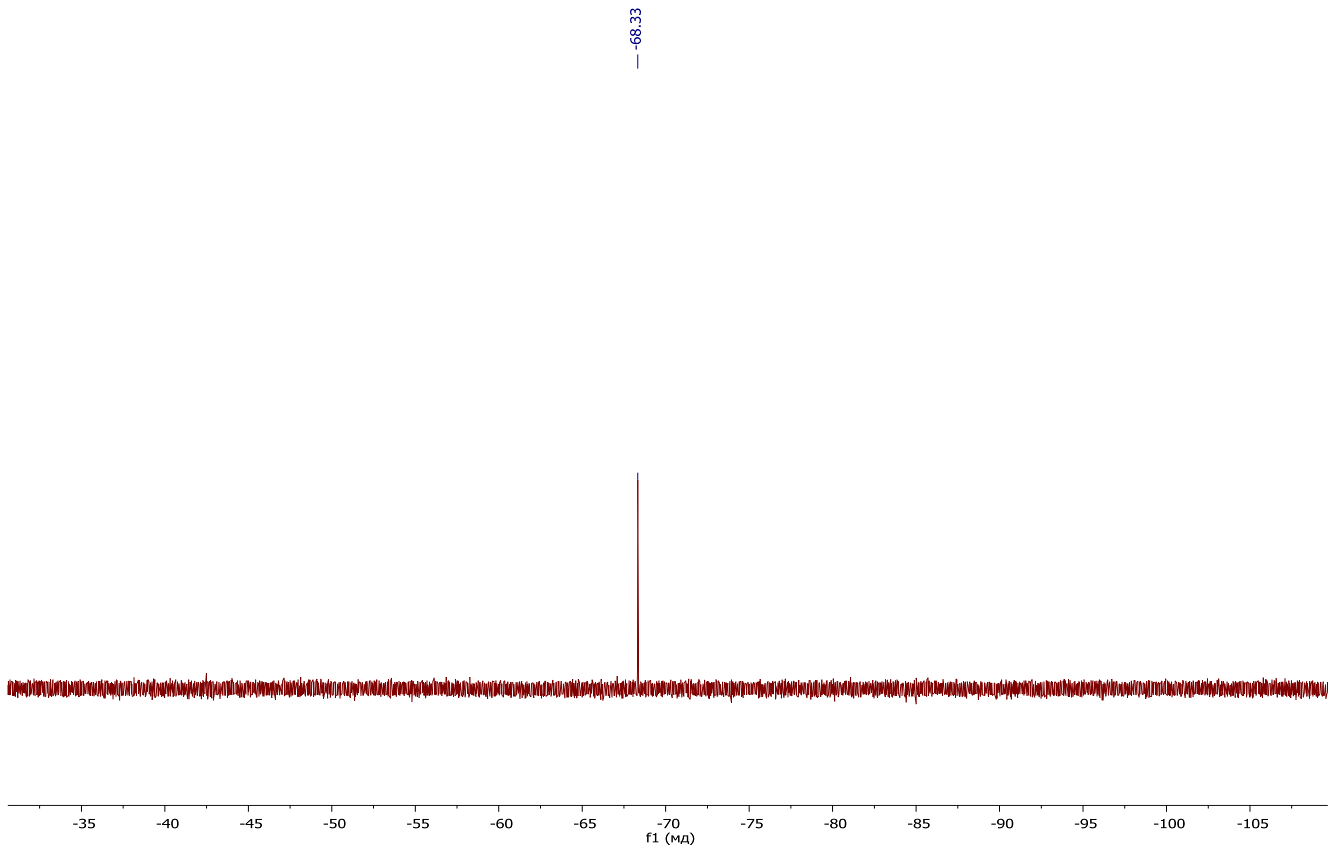

Figure S13. ^{19}F NMR of **3c** in $CDCl_3$

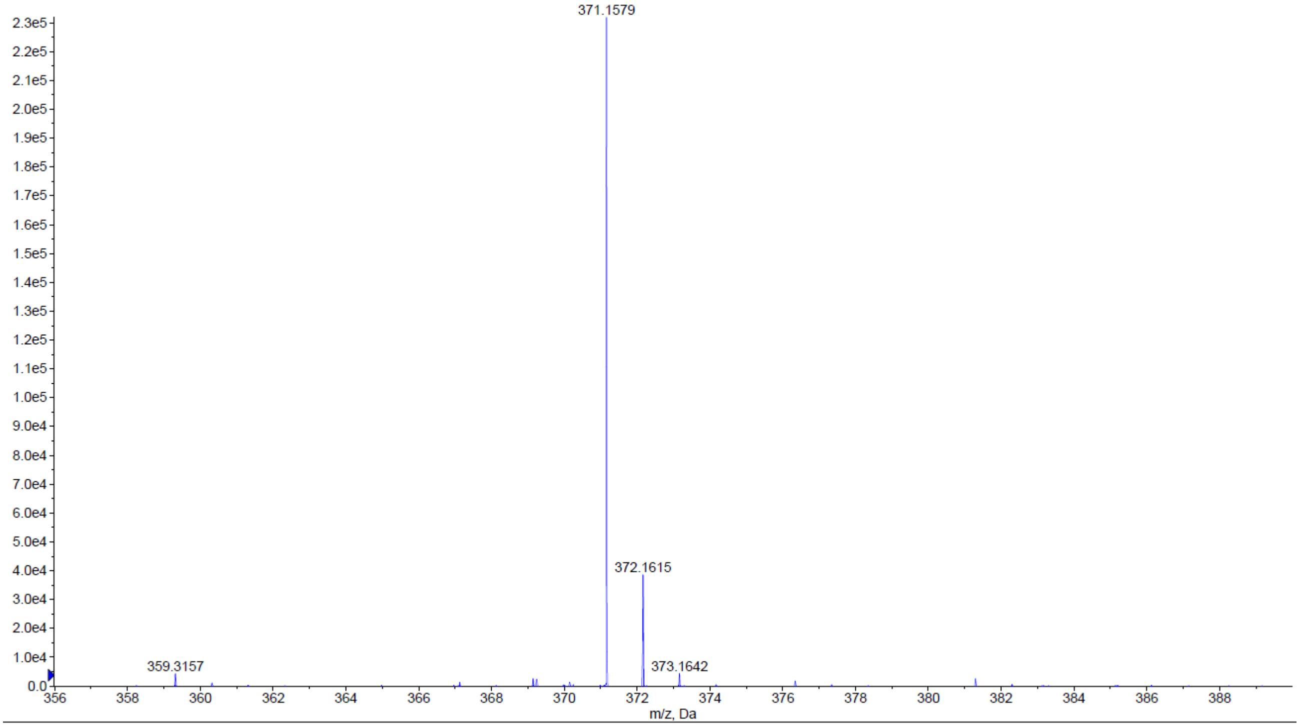

Figure S14. HRMS of **3c**

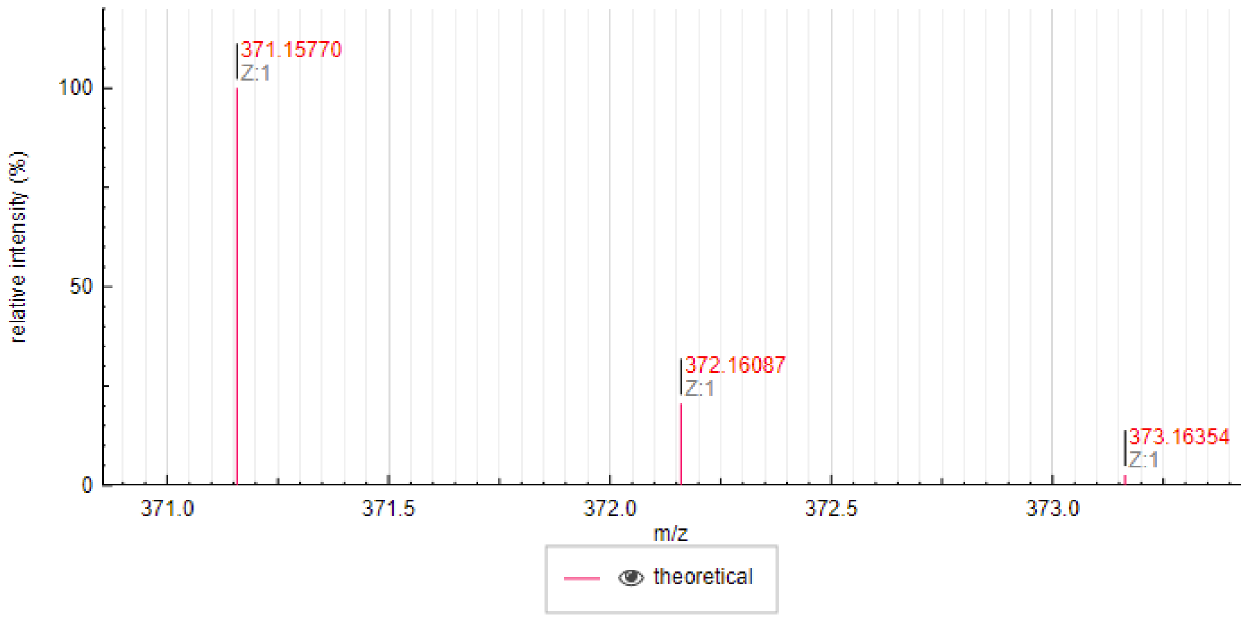

Figure S15. Theoretical HRMS $[M+H]^+$ of **3c**

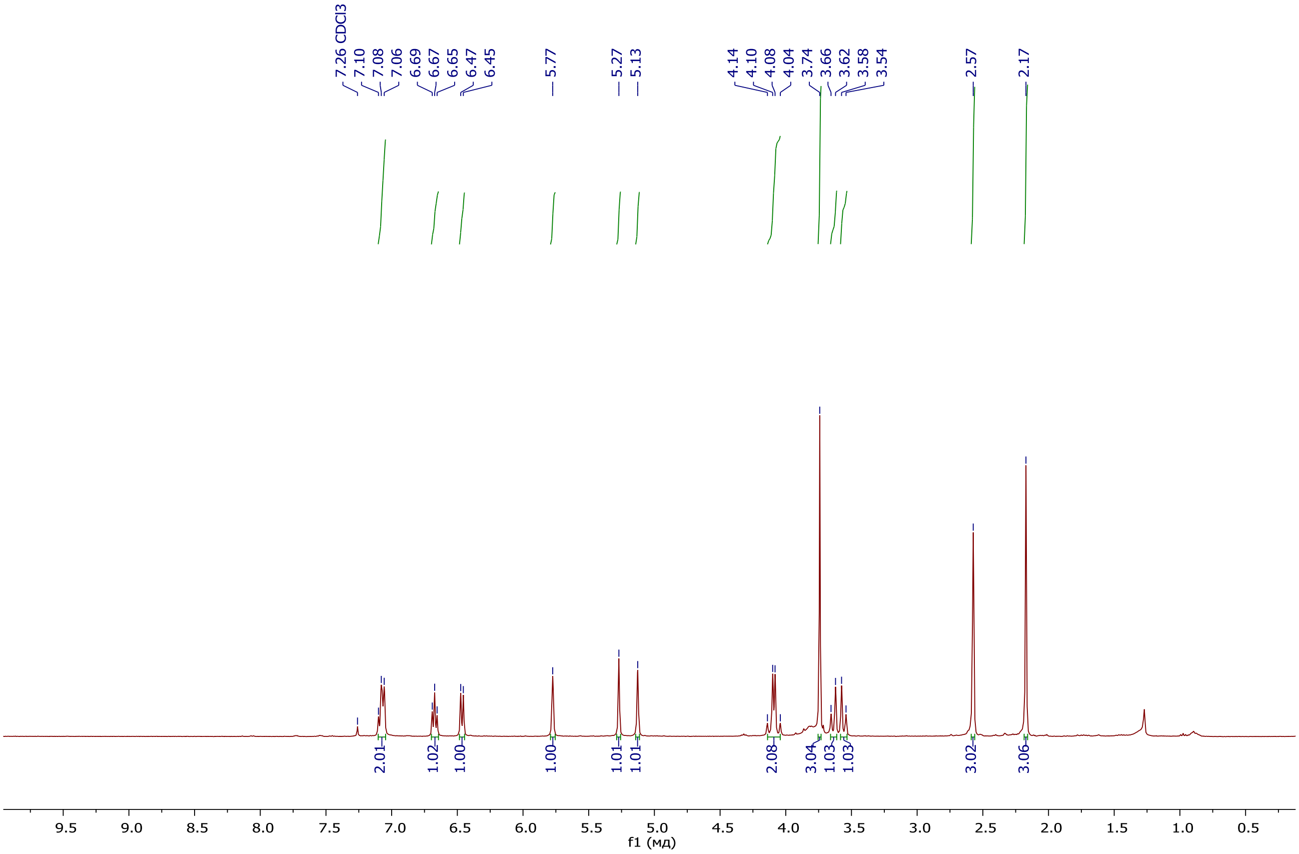

Figure S16. ^{1}H NMR of **3d** in $CDCl_3$

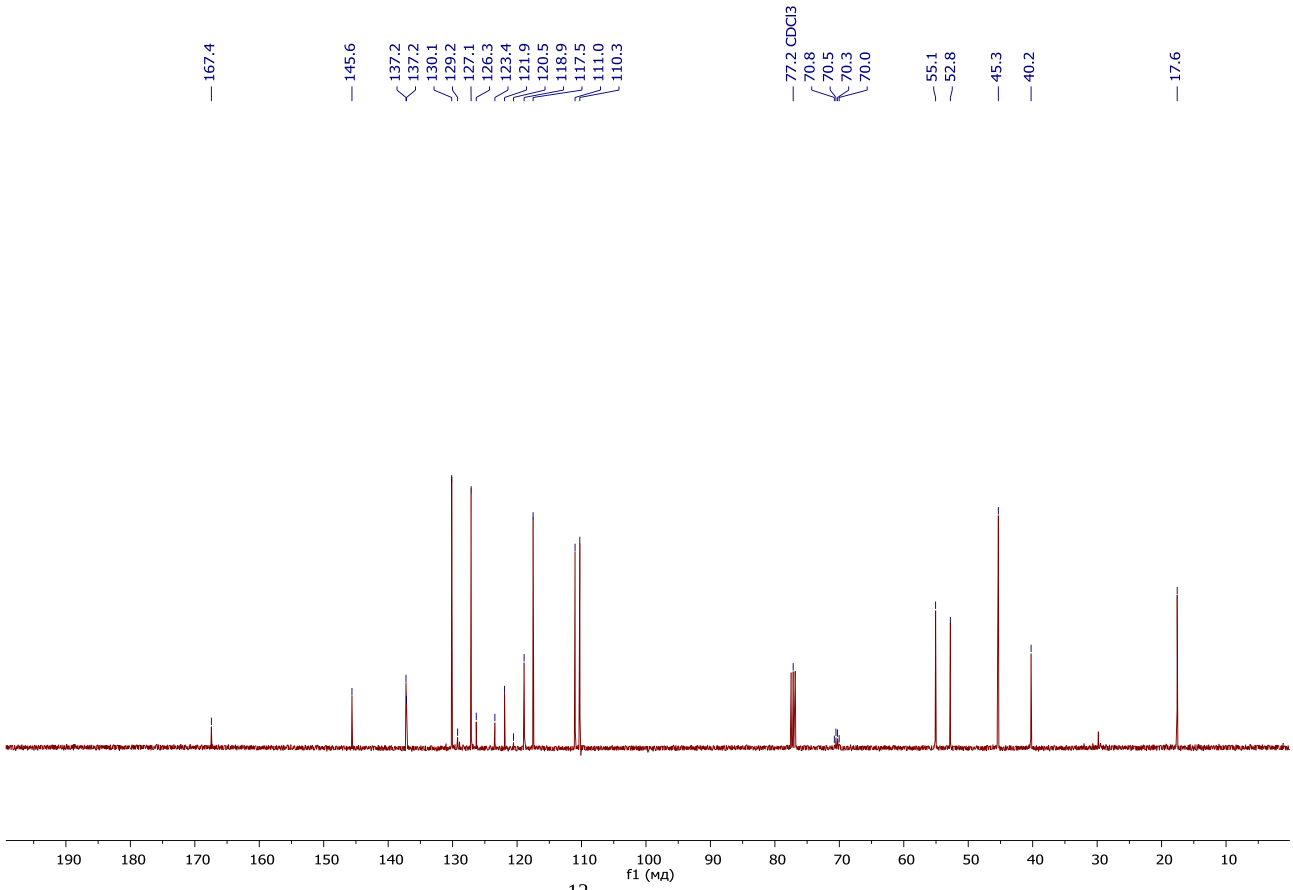

Figure S17. ^{13}C NMR of **3d** in $CDCl_3$

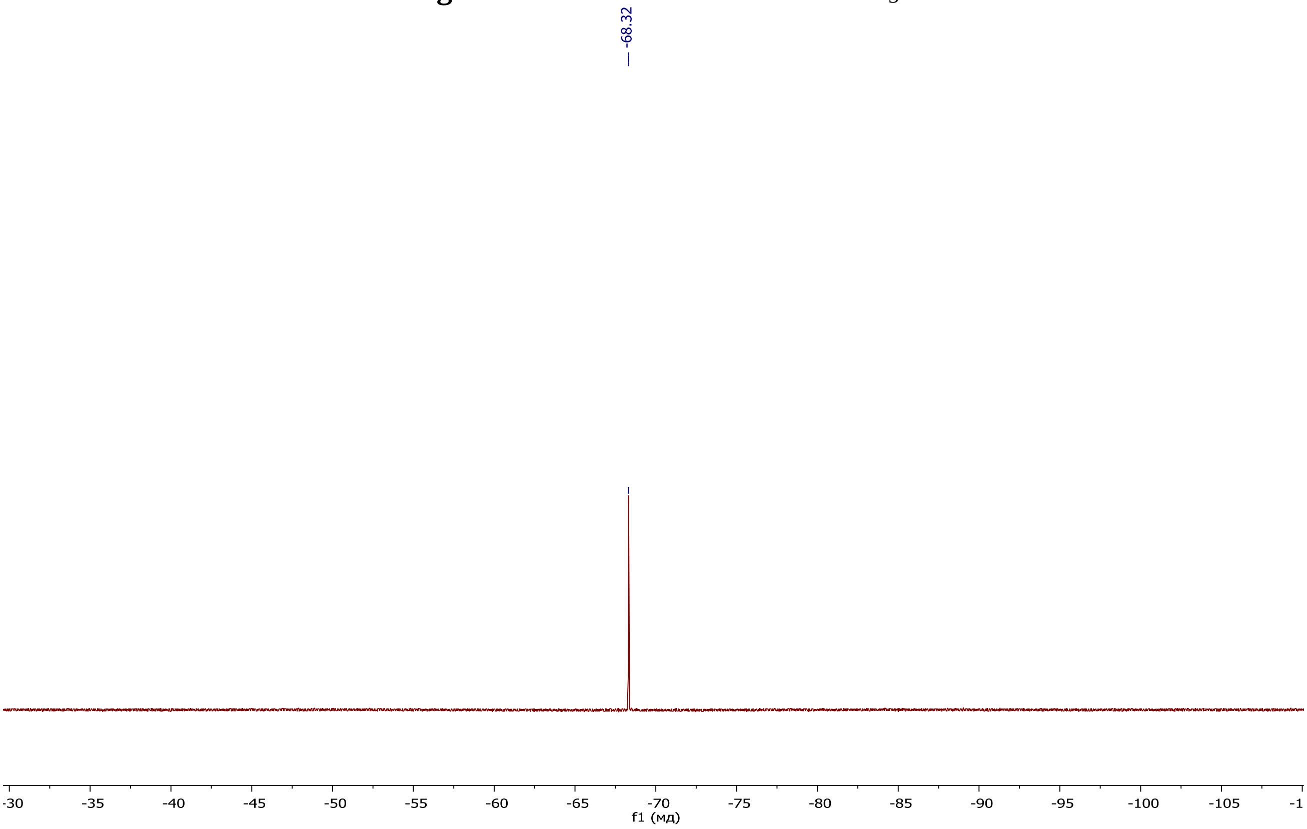

Figure S18. ^{19}F NMR of **3d** in $CDCl_3$

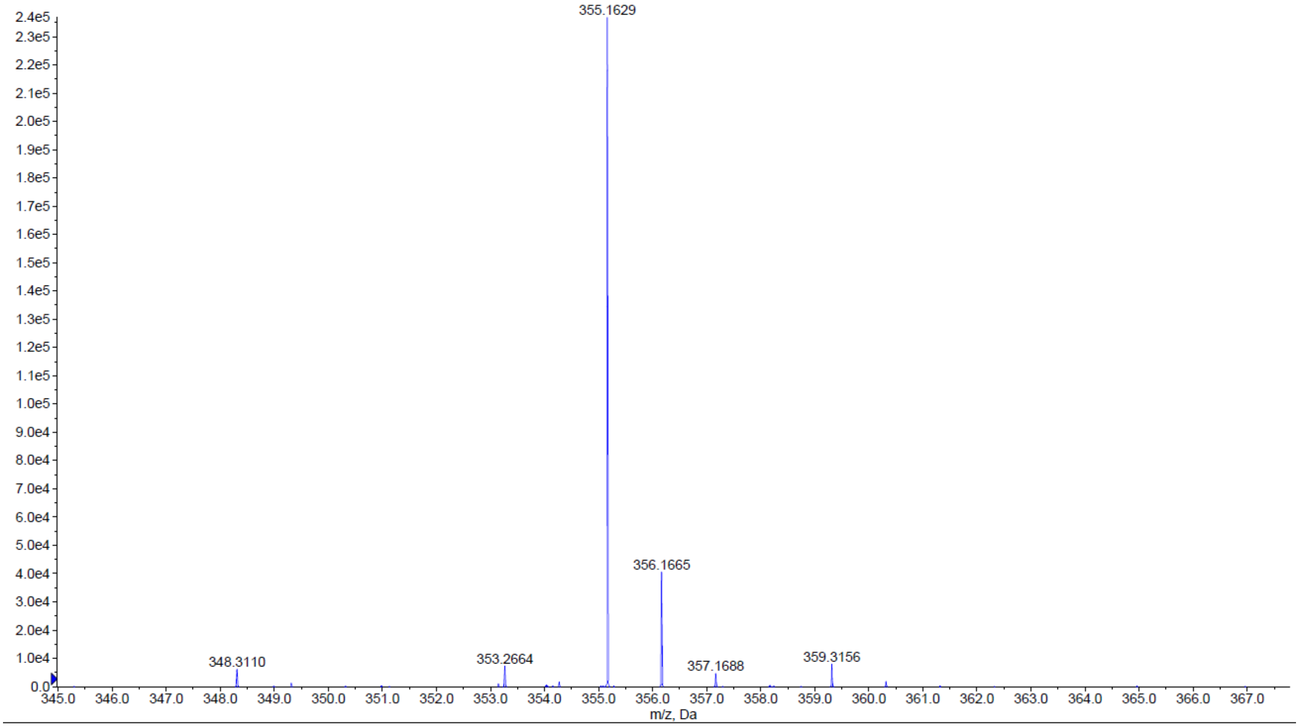

Figure S19. HRMS of **3d**

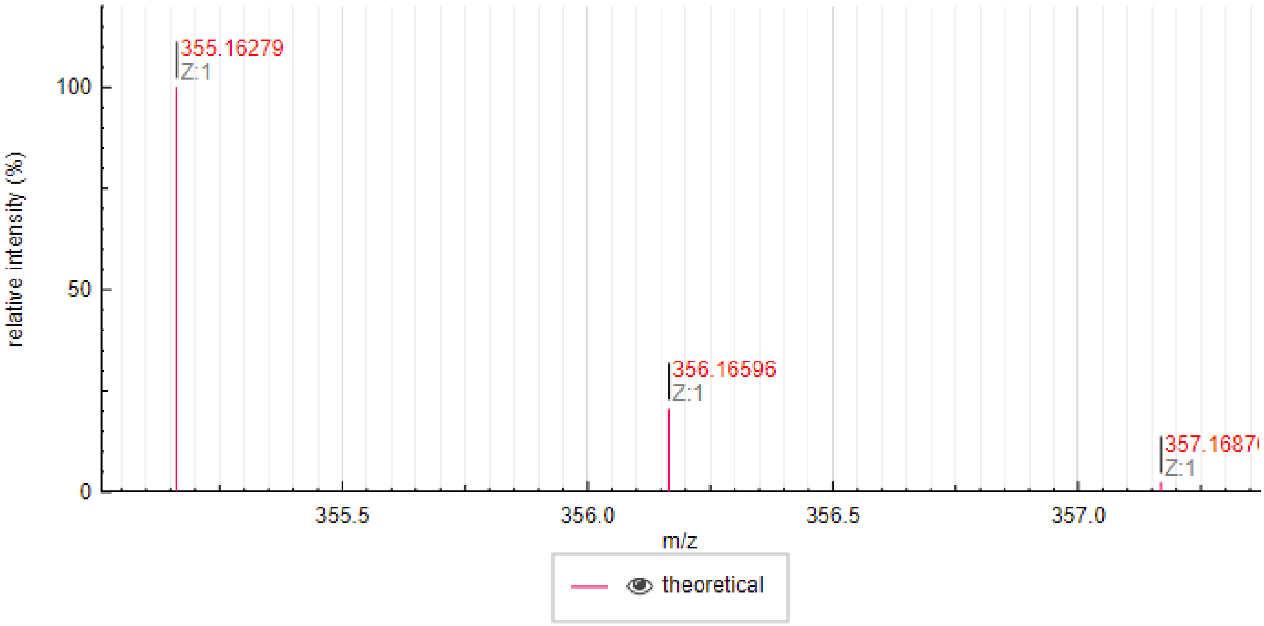

Figure S20. Theoretical HRMS $[M+H]^+$ of **3d**

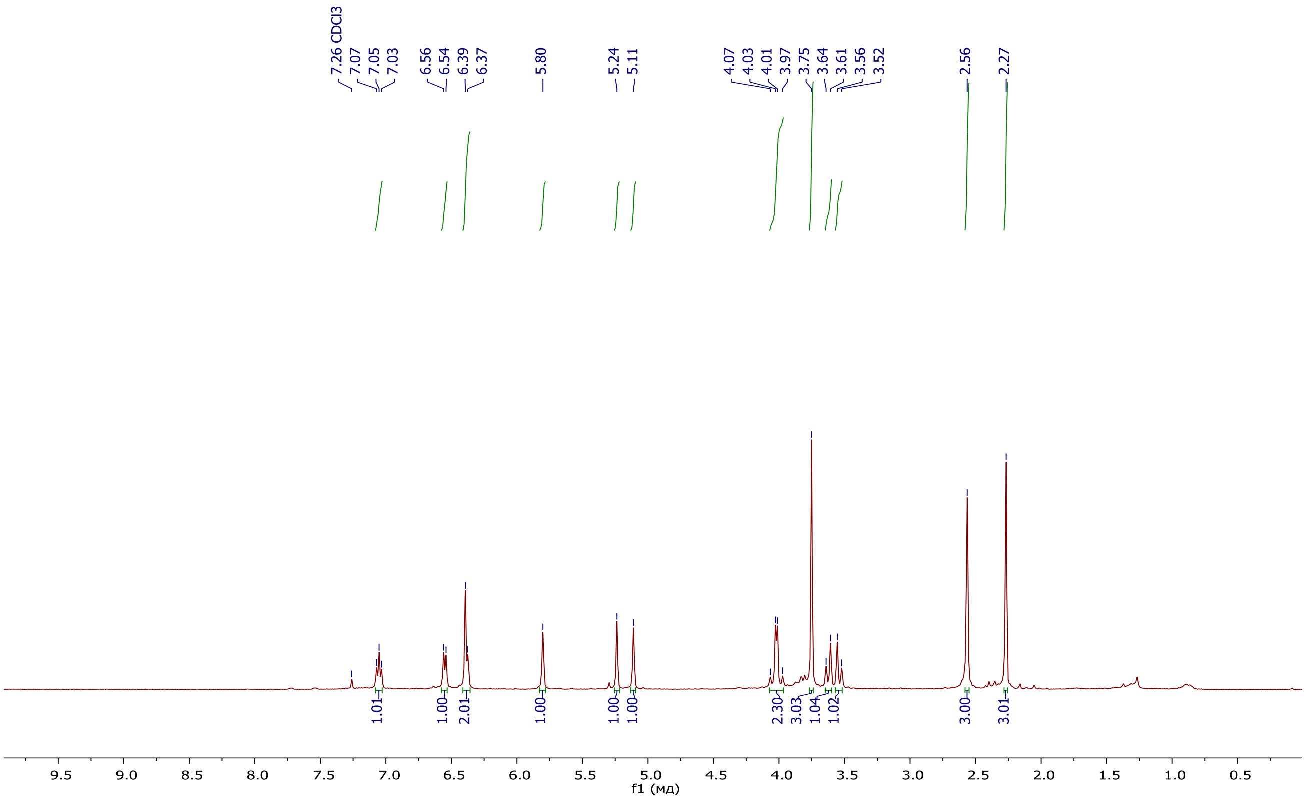

Figure S21. ^{1}H NMR of **3e** in $CDCl_3$

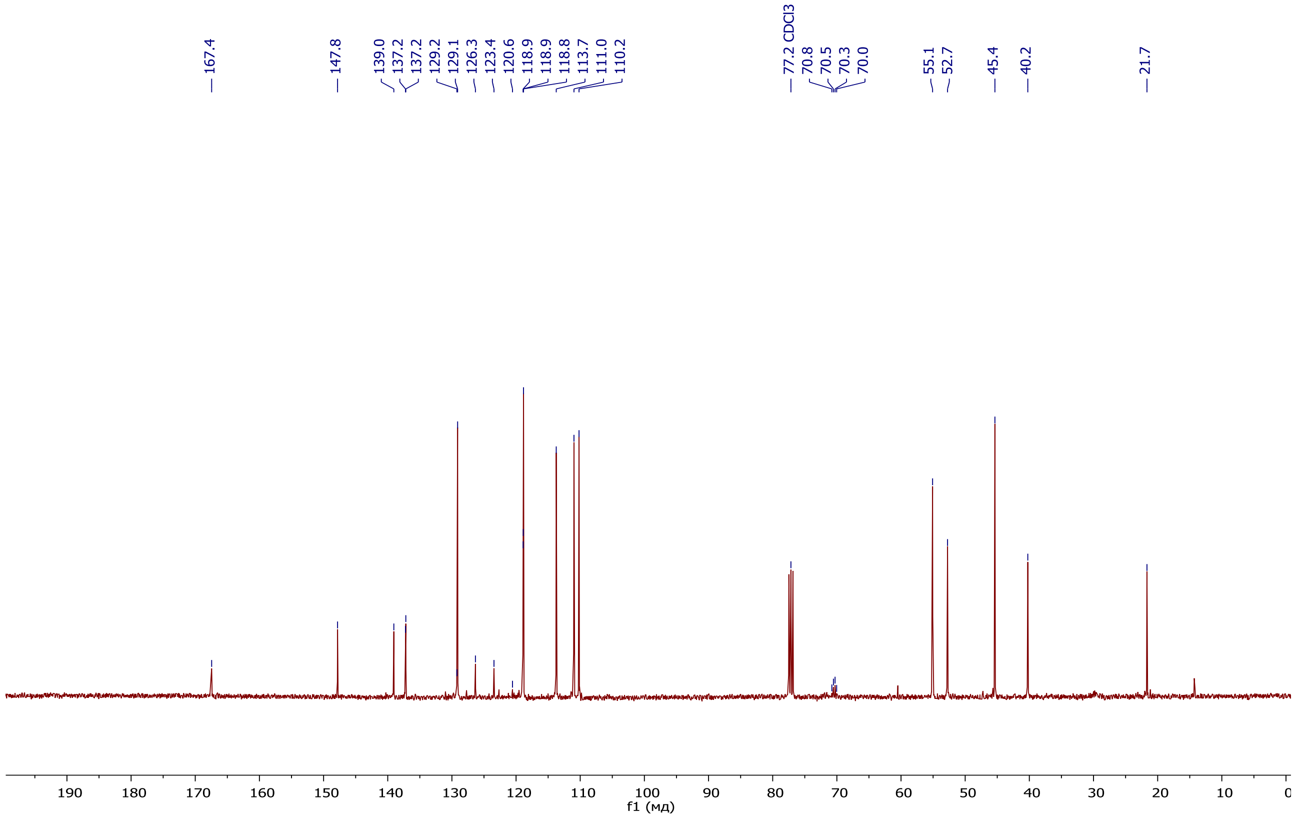

Figure S22. ^{13}C NMR of **3e** in $CDCl_3$

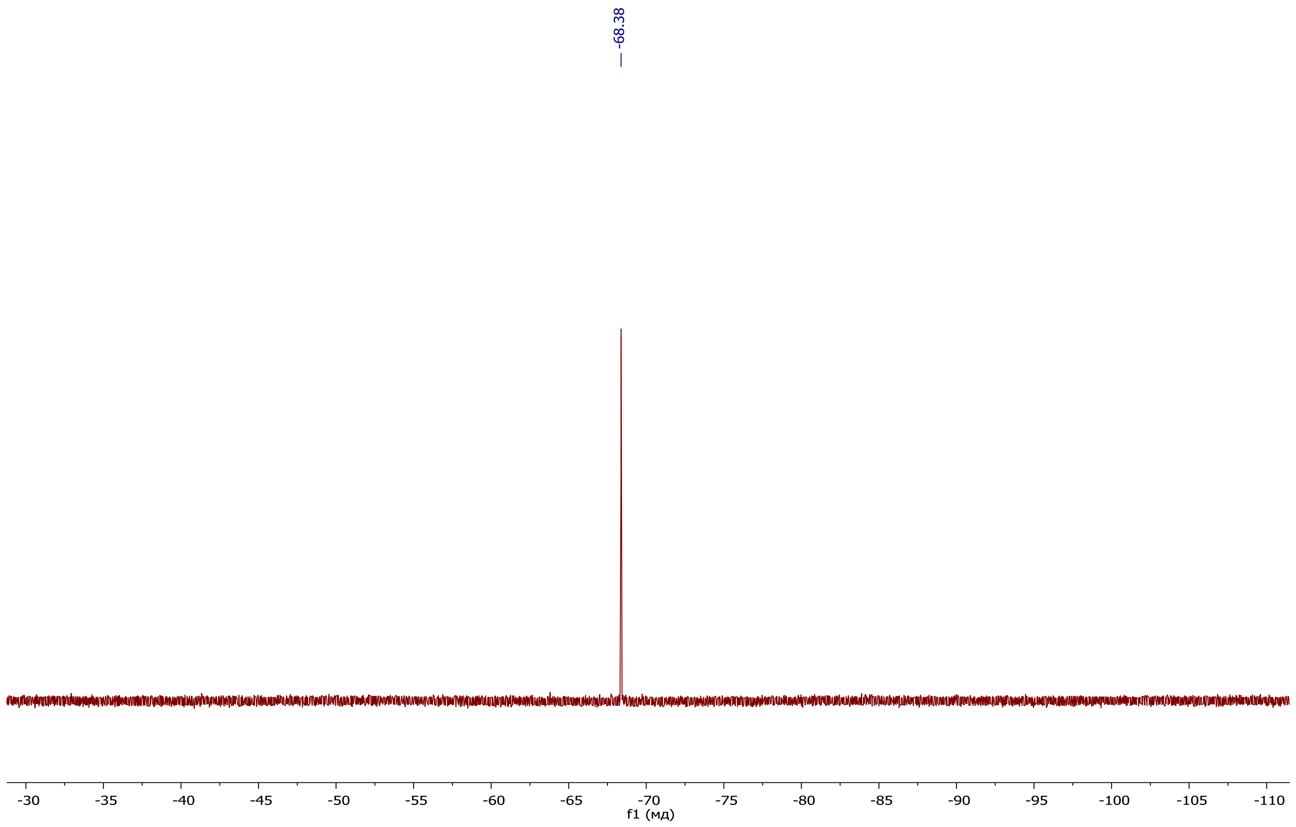

Figure S23. ^{19}F NMR of **3e** in $CDCl_3$

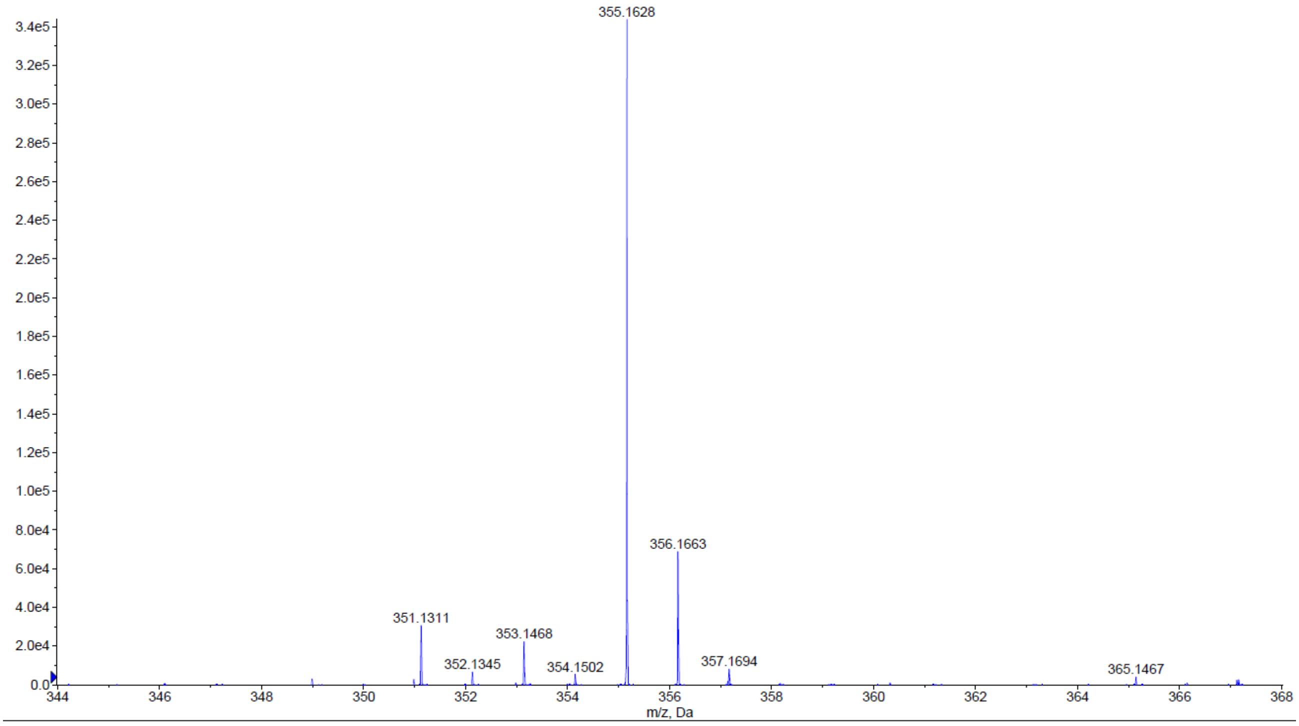

Figure S24. HRMS of **3e**

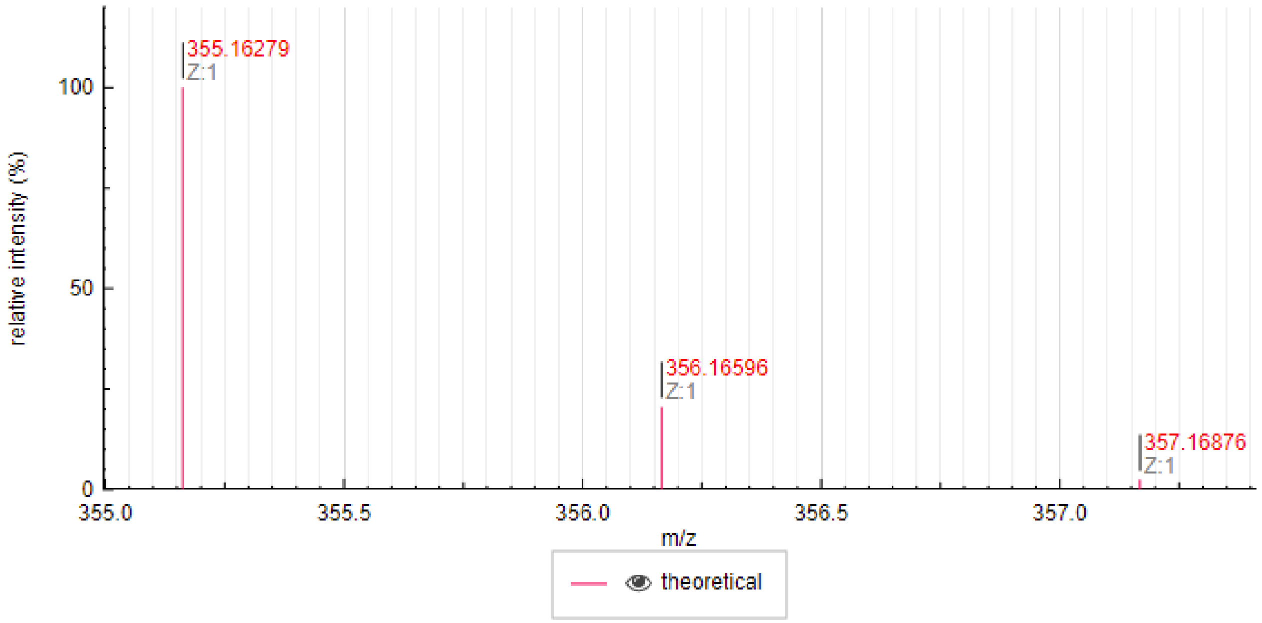

Figure S25. Theoretical HRMS [M+H]$^+$ of **3e**

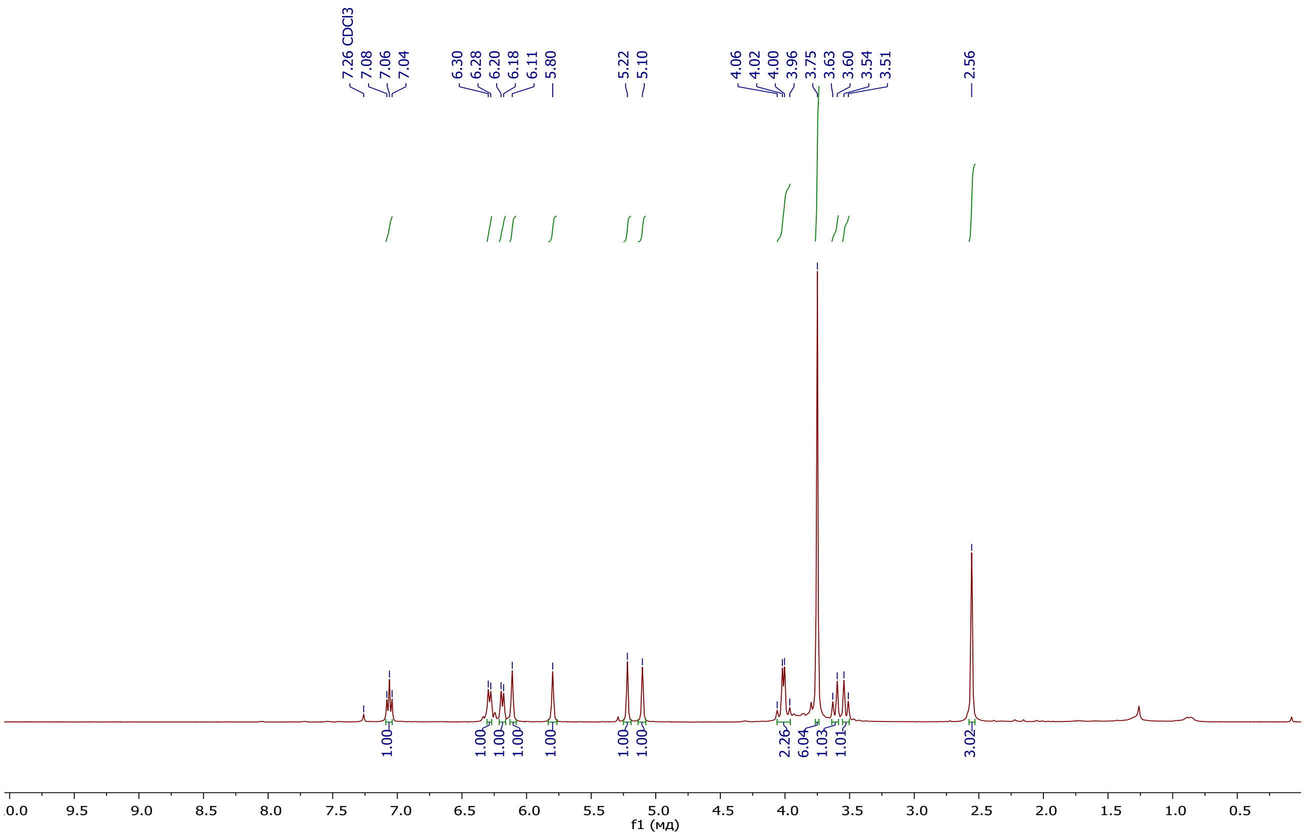

Figure S26. ^{1}H NMR of **3f** in $CDCl_3$

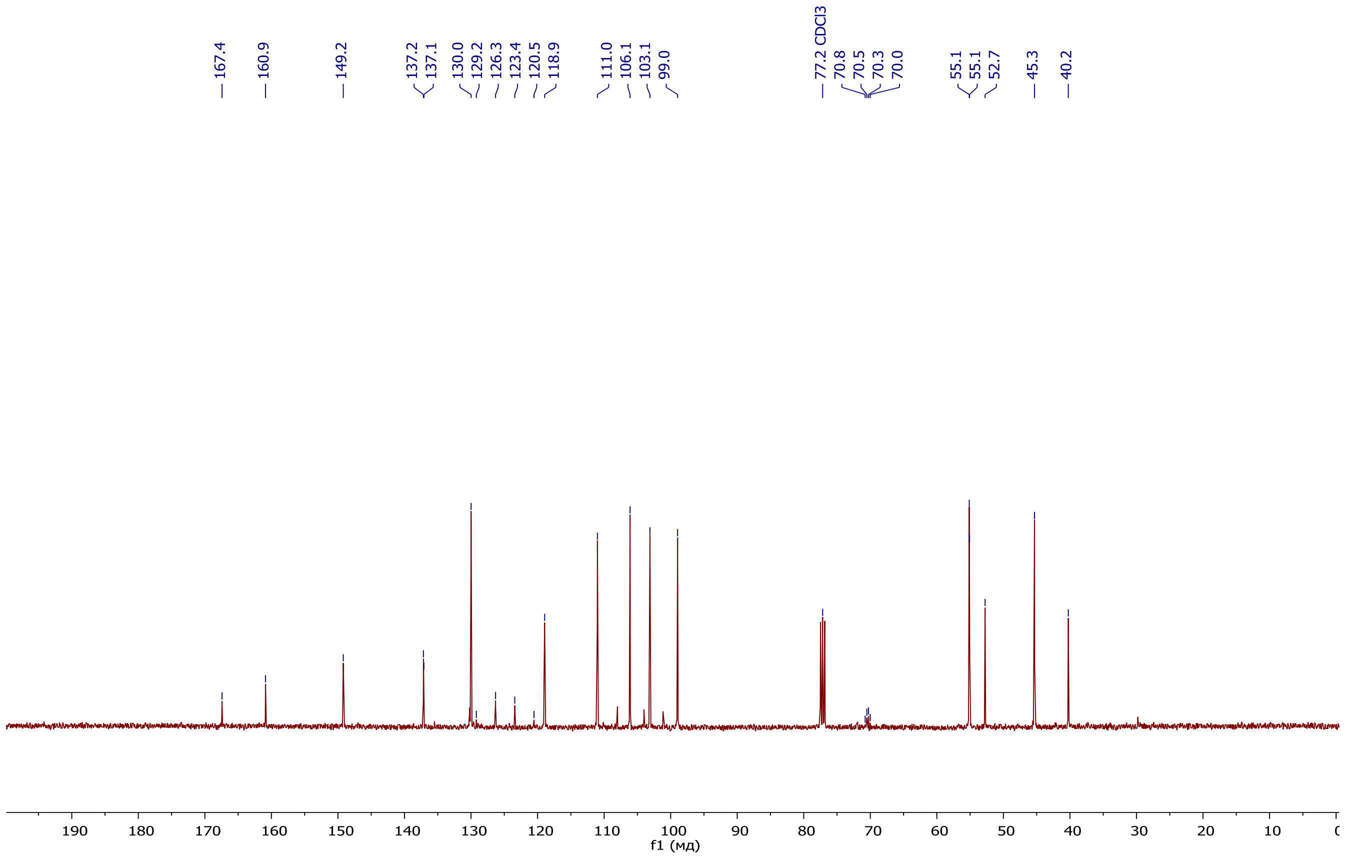

Figure S27. ^{13}C NMR of **3f** in $CDCl_3$

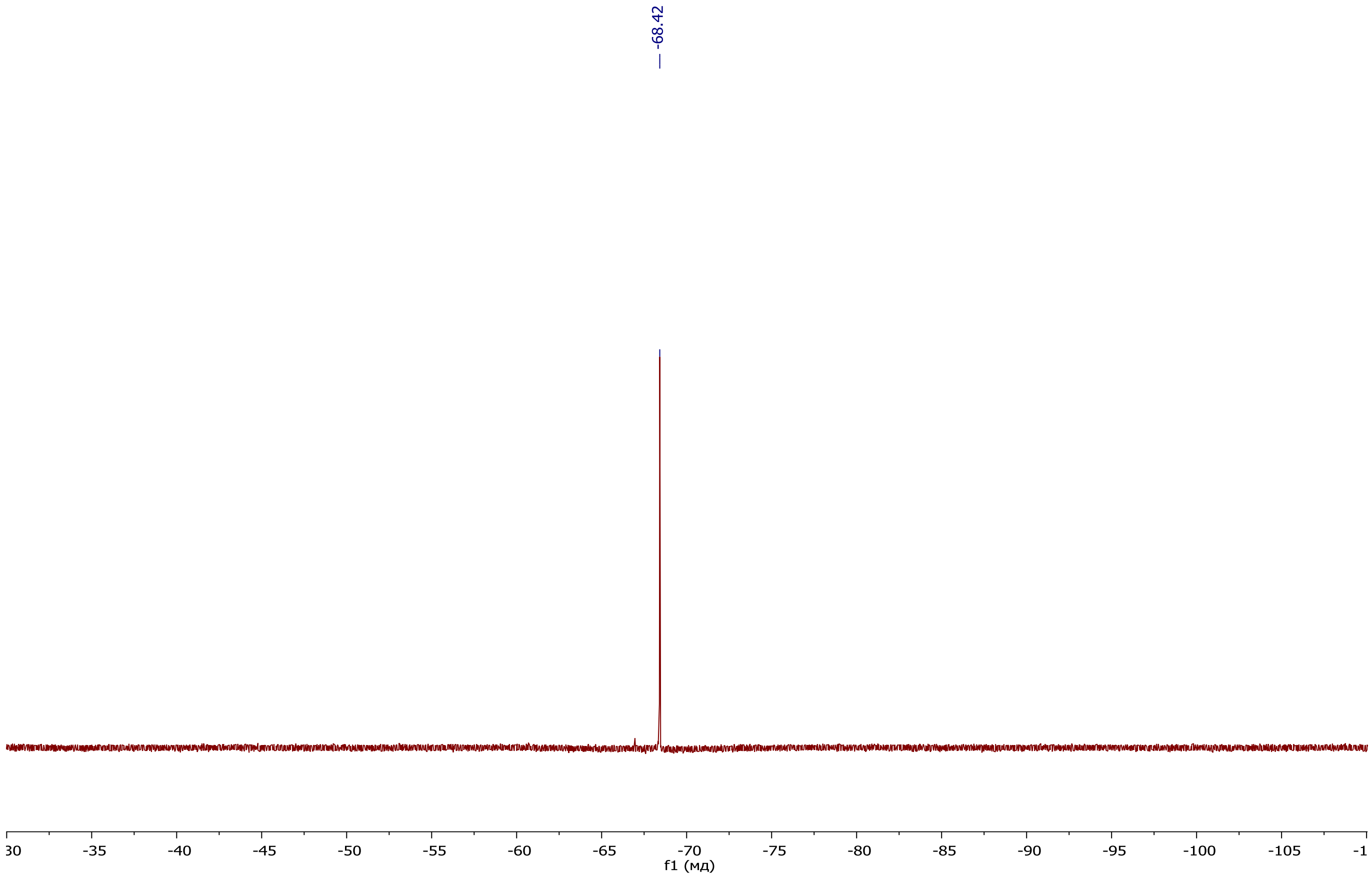

Figure S28. ^{19}F NMR of **3f** in $CDCl_3$

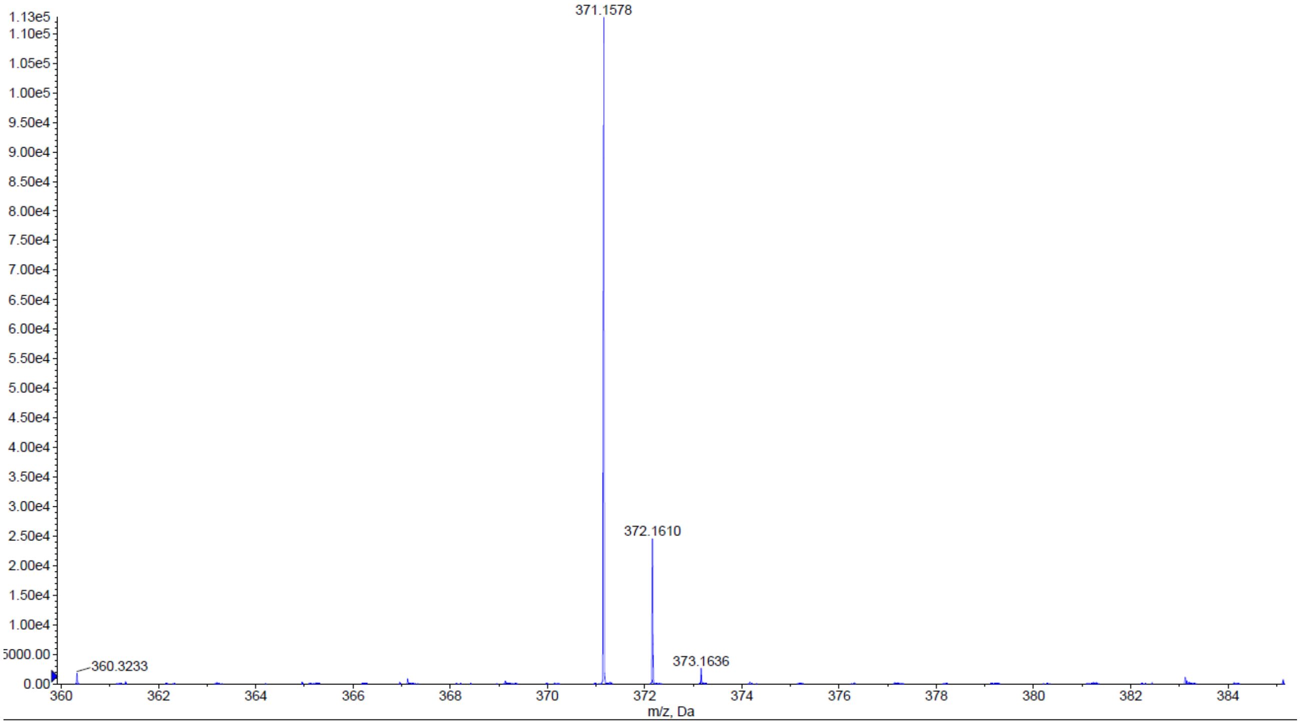

Figure S29. HRMS of **3f**

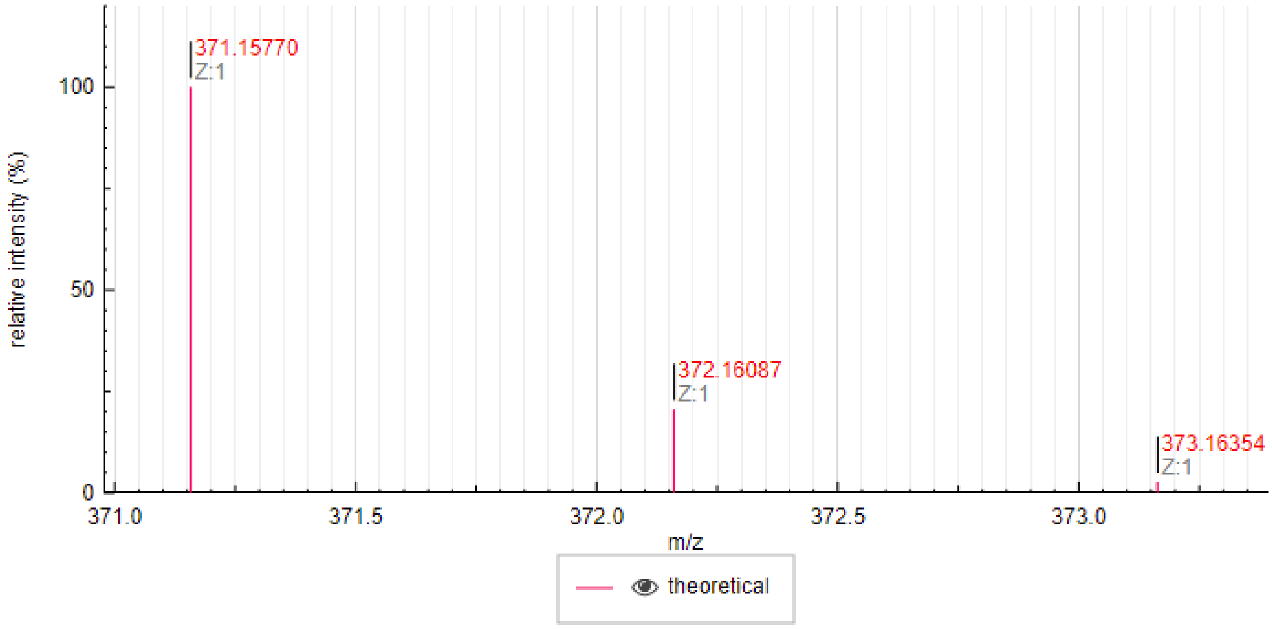

Figure S30. Theoretical HRMS $[M+H]^+$ of **3f**

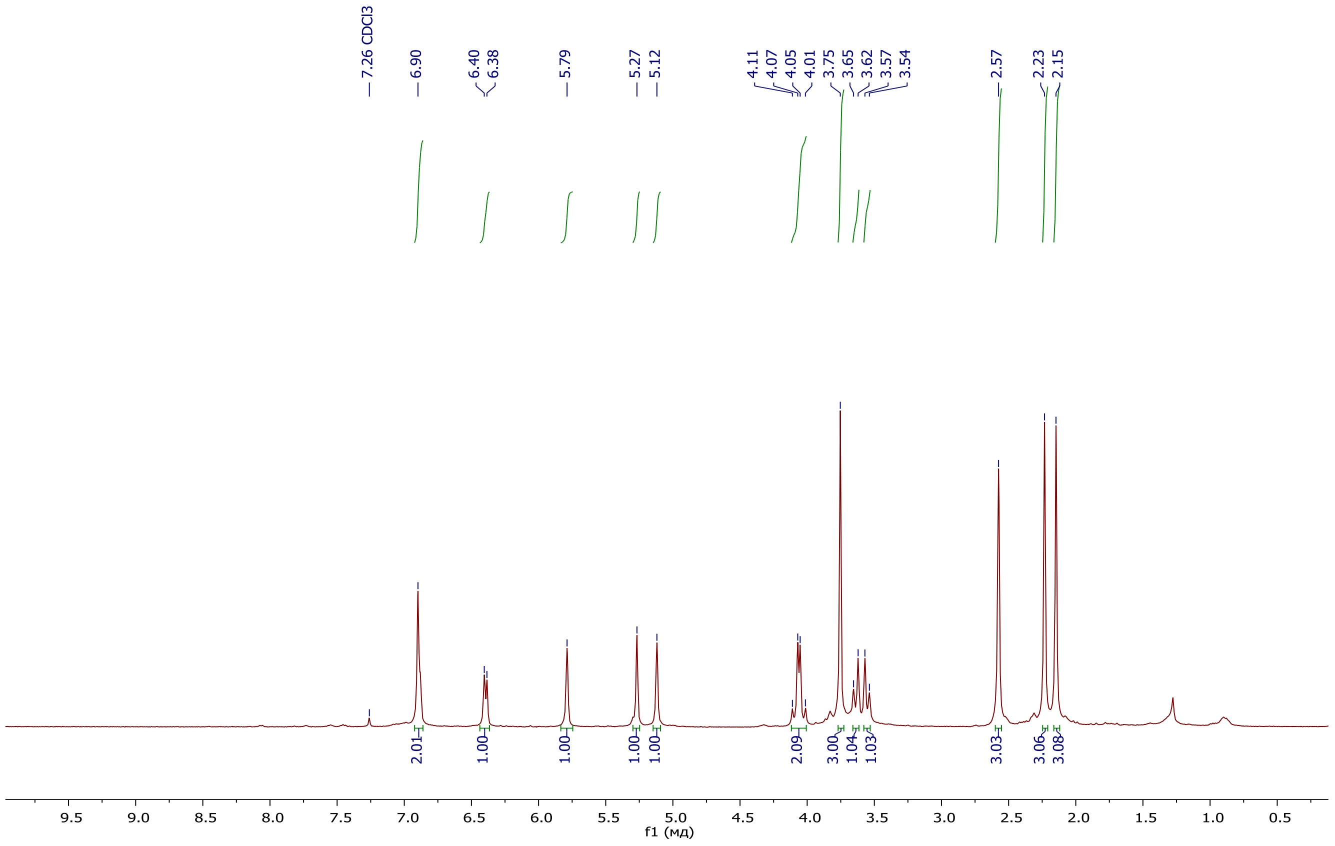

Figure S31. ^{1}H NMR of **3g** in $CDCl_3$

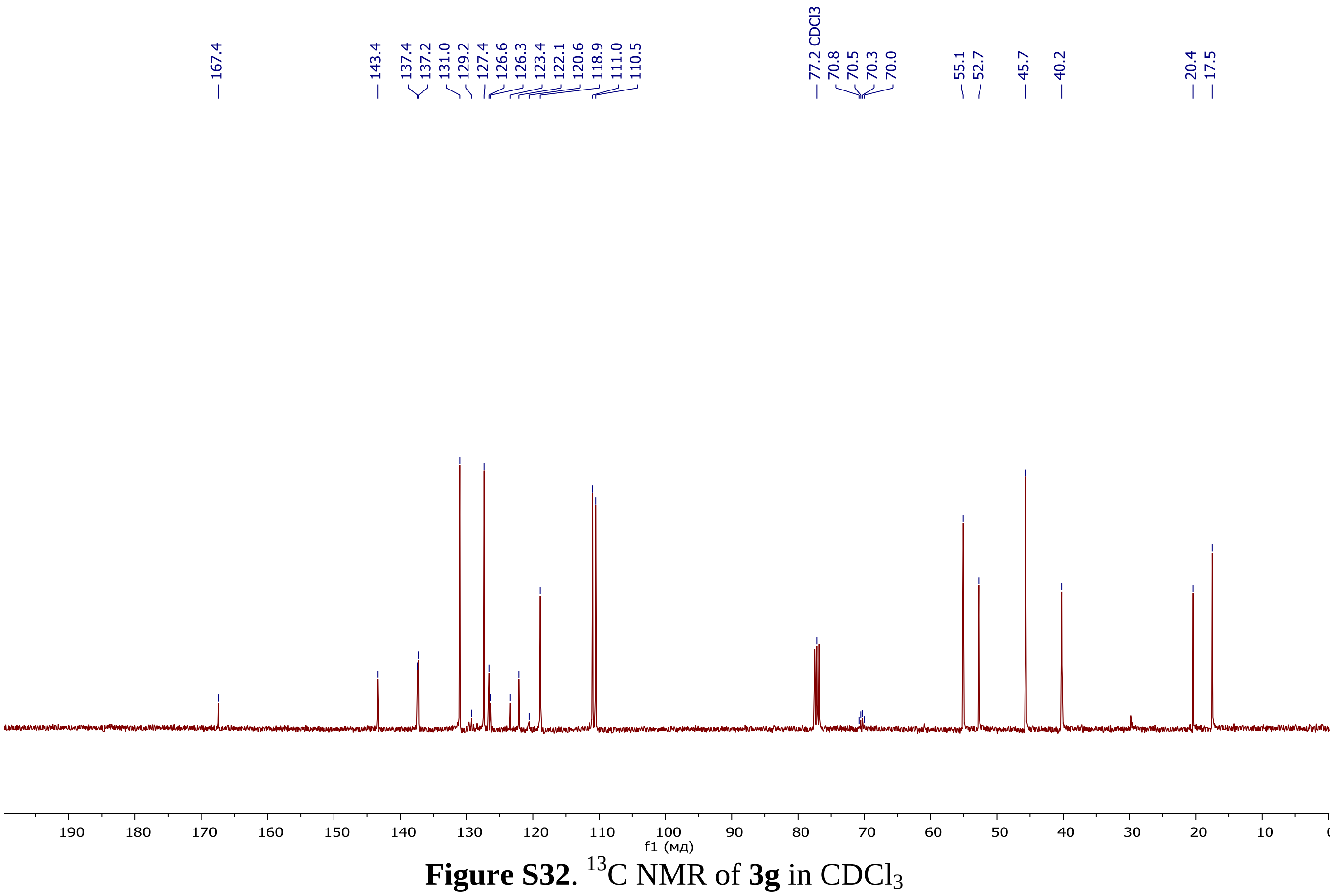

Figure S32. ^{13}C NMR of **3g** in $CDCl_3$

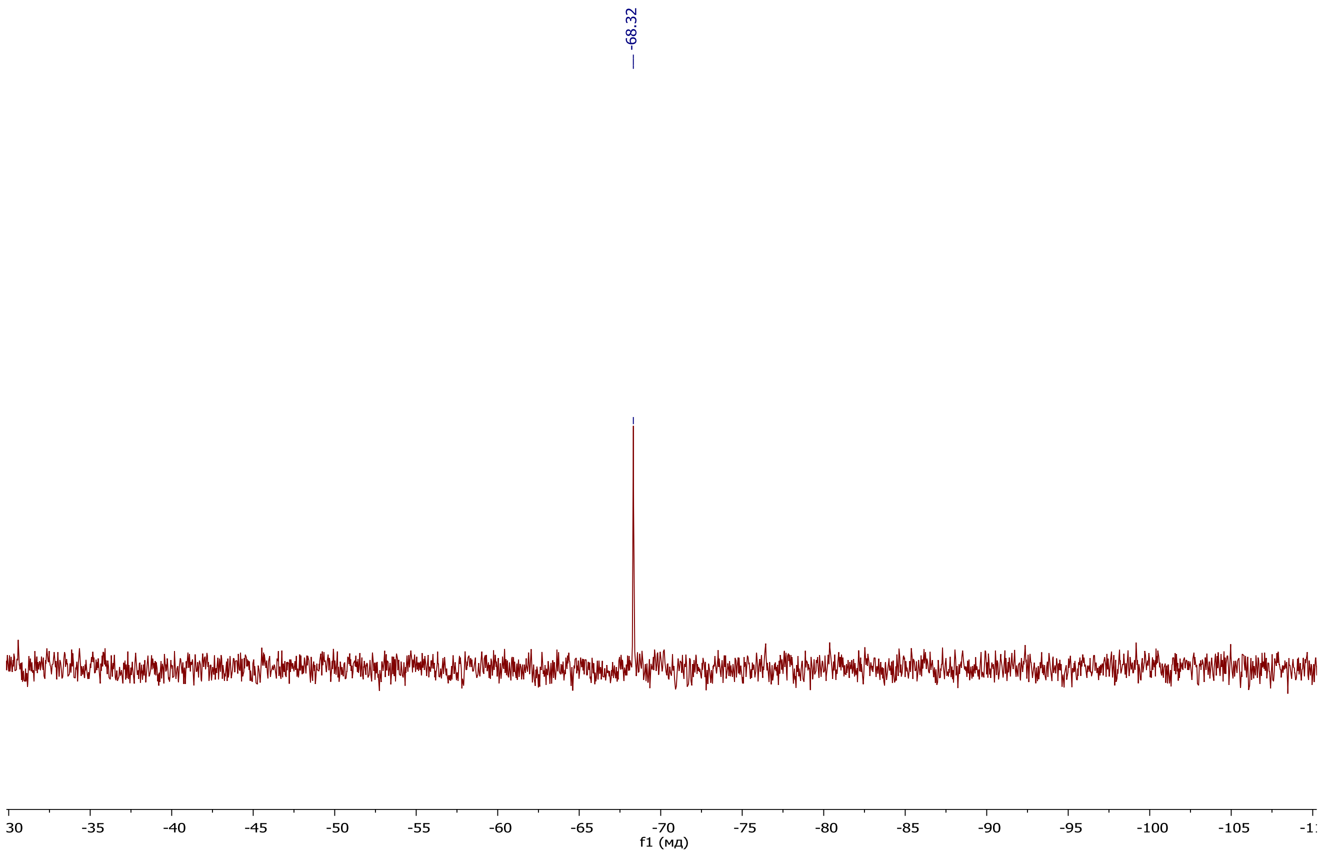

Figure S33. ^{19}F NMR of **3g** in $CDCl_3$

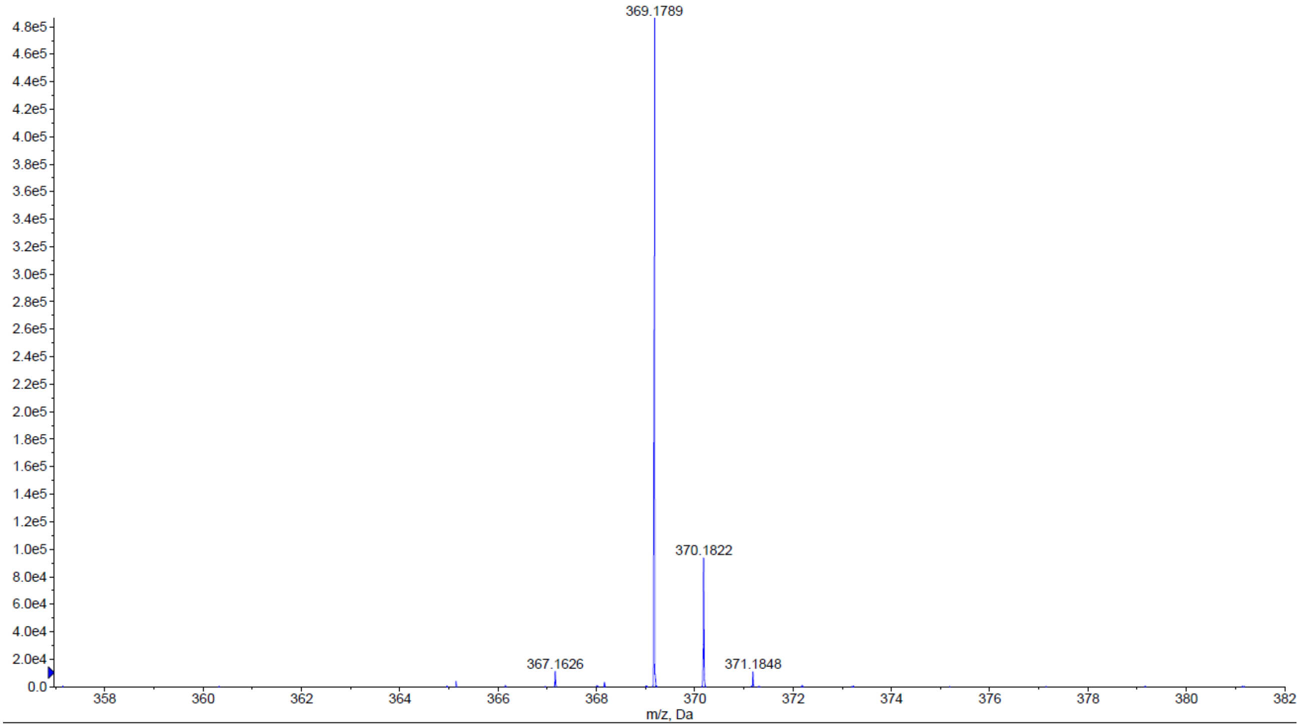

Figure S34. HRMS of **3g**

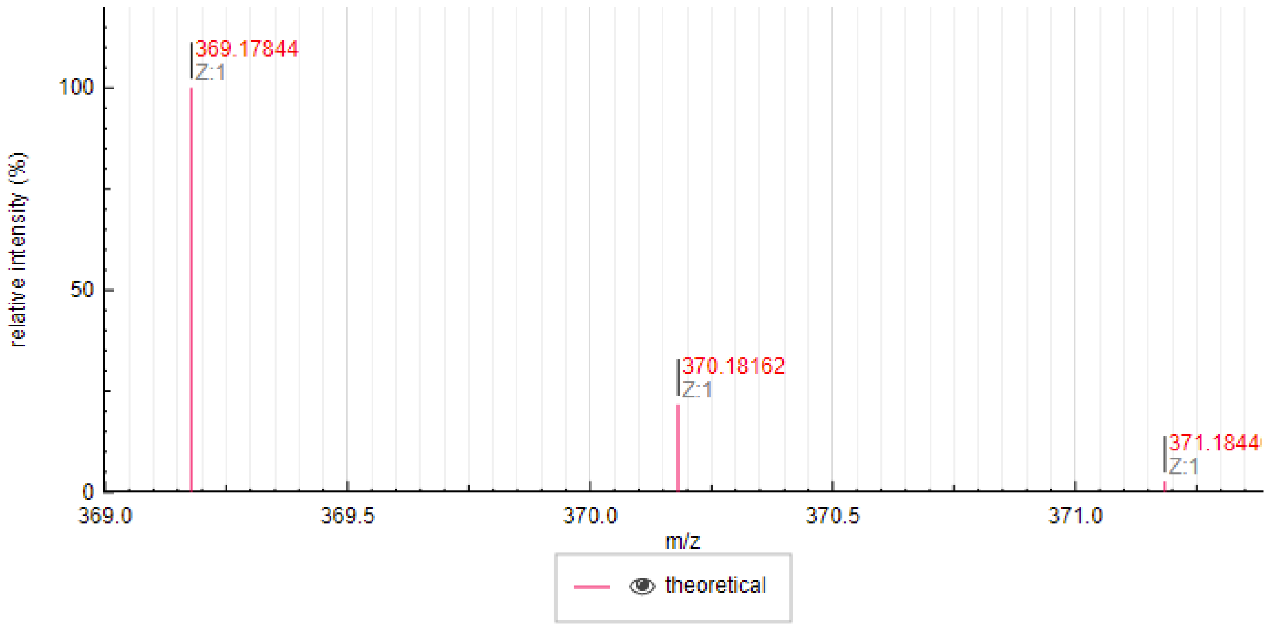

Figure S35. Theoretical HRMS $[M+H]^+$ of **3g**

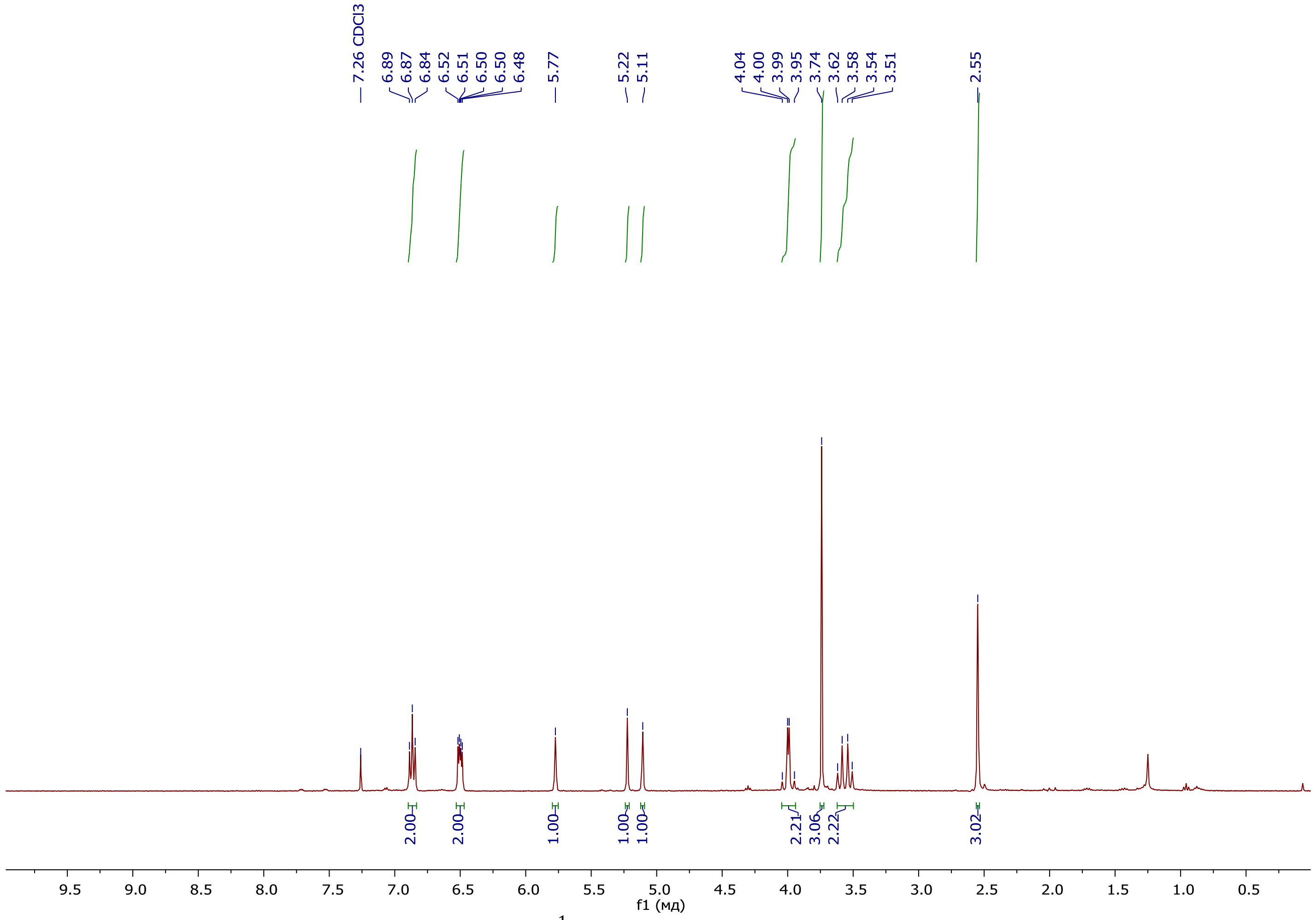

Figure S36. ^{1}H NMR of **3h** in $CDCl_3$

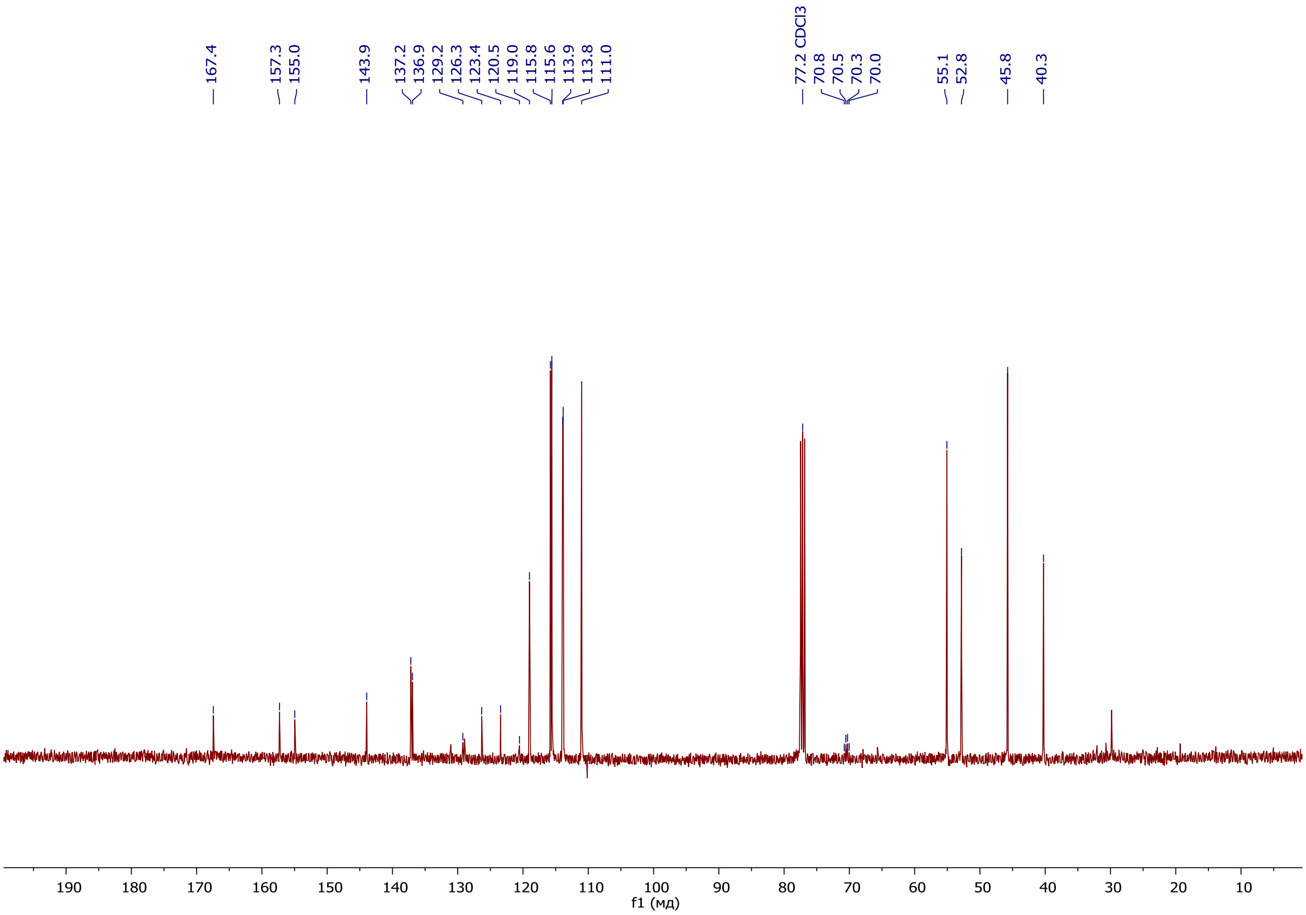

Figure S37. ^{13}C NMR of **3h** in $CDCl_3$

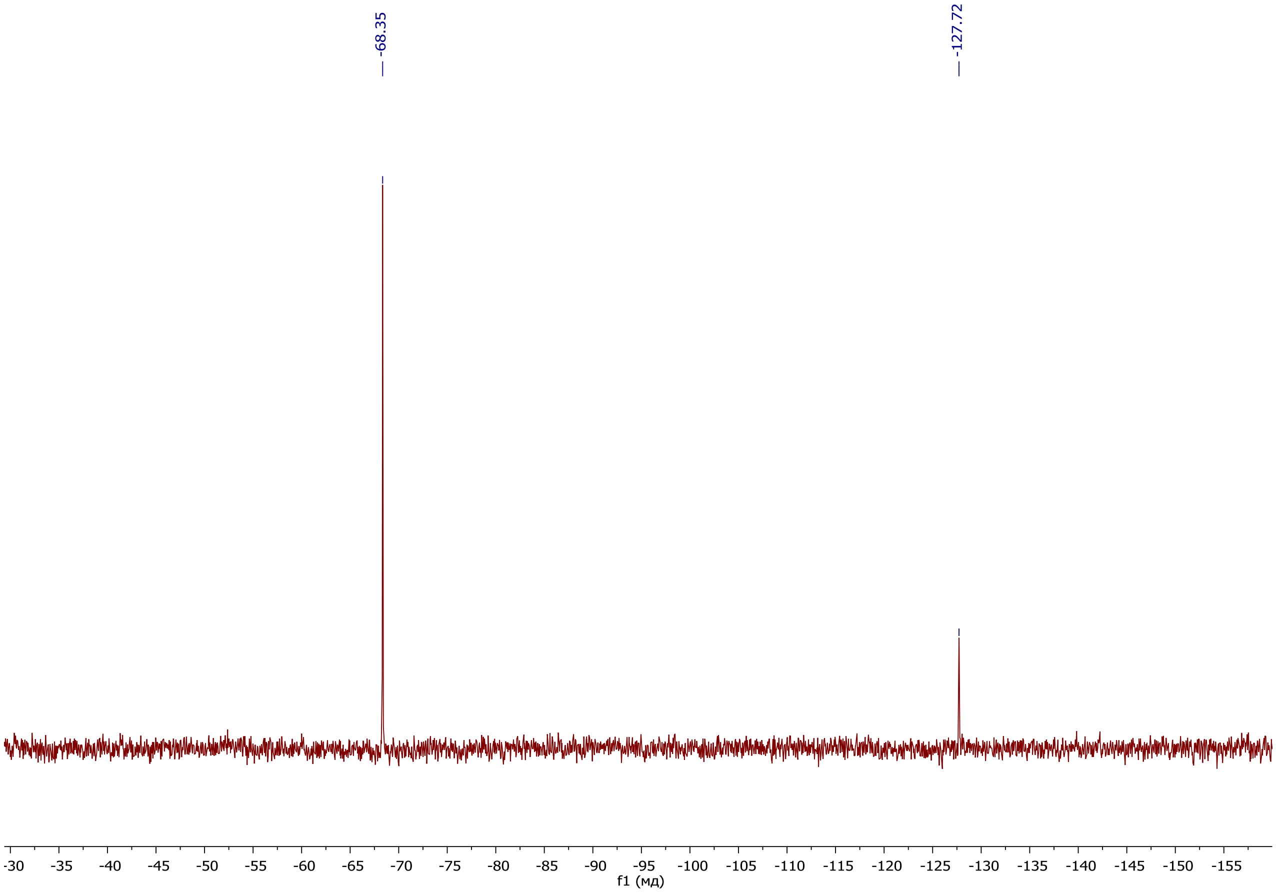

Figure S38. ^{19}F NMR of **3h** in $CDCl_3$

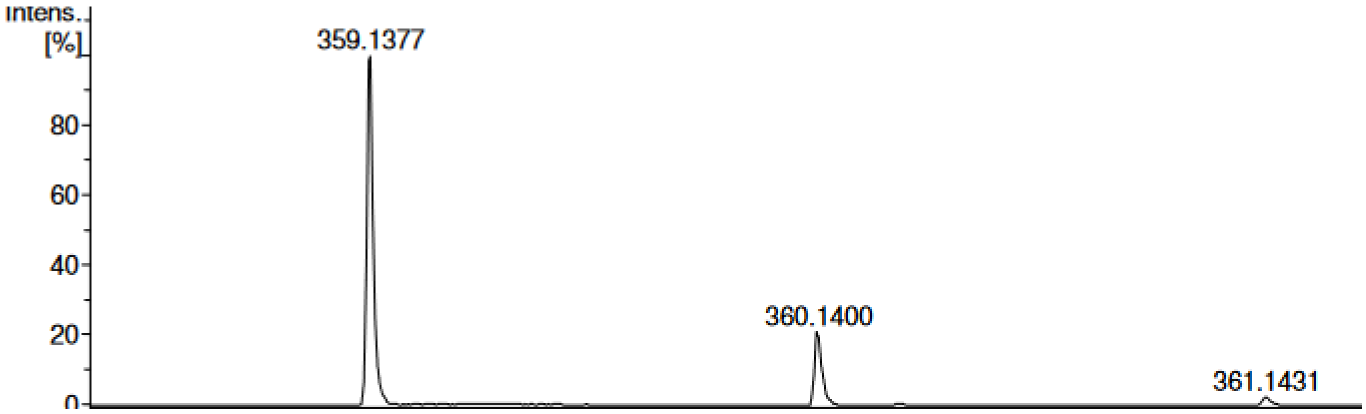

Figure S39. HRMS of **3h**

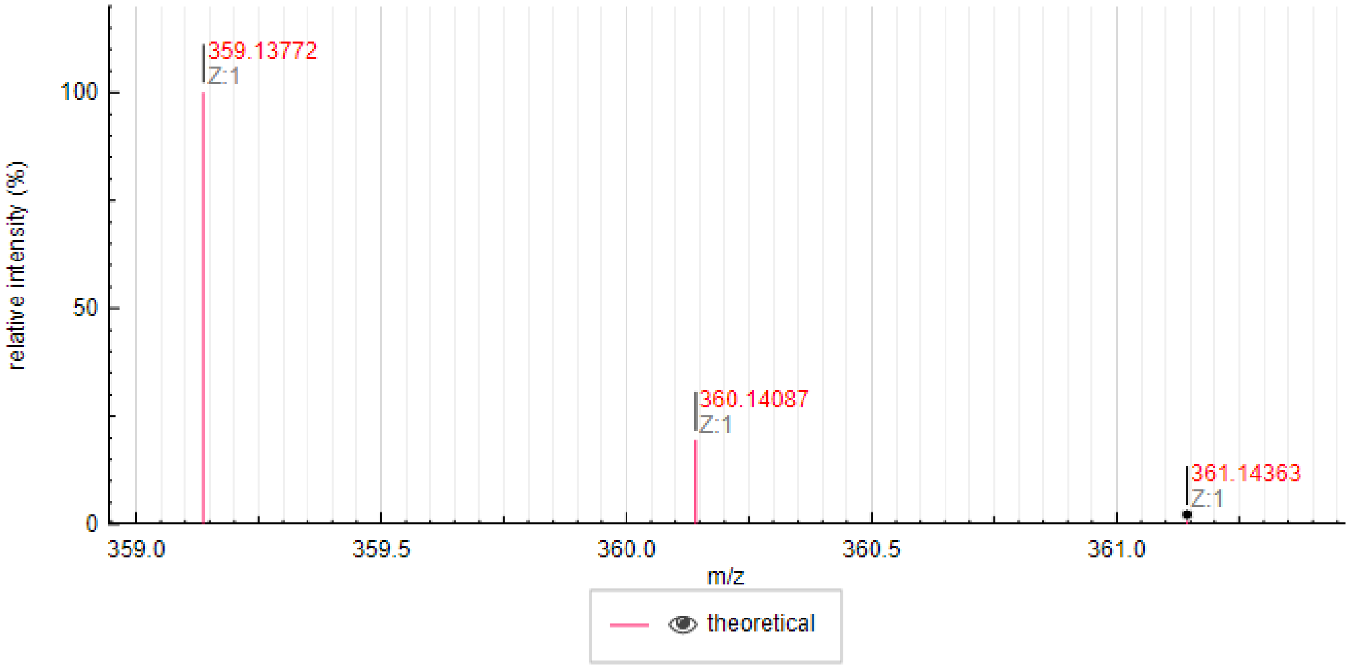

Figure S40. Theoretical HRMS $[M+H]^+$ of **3h**

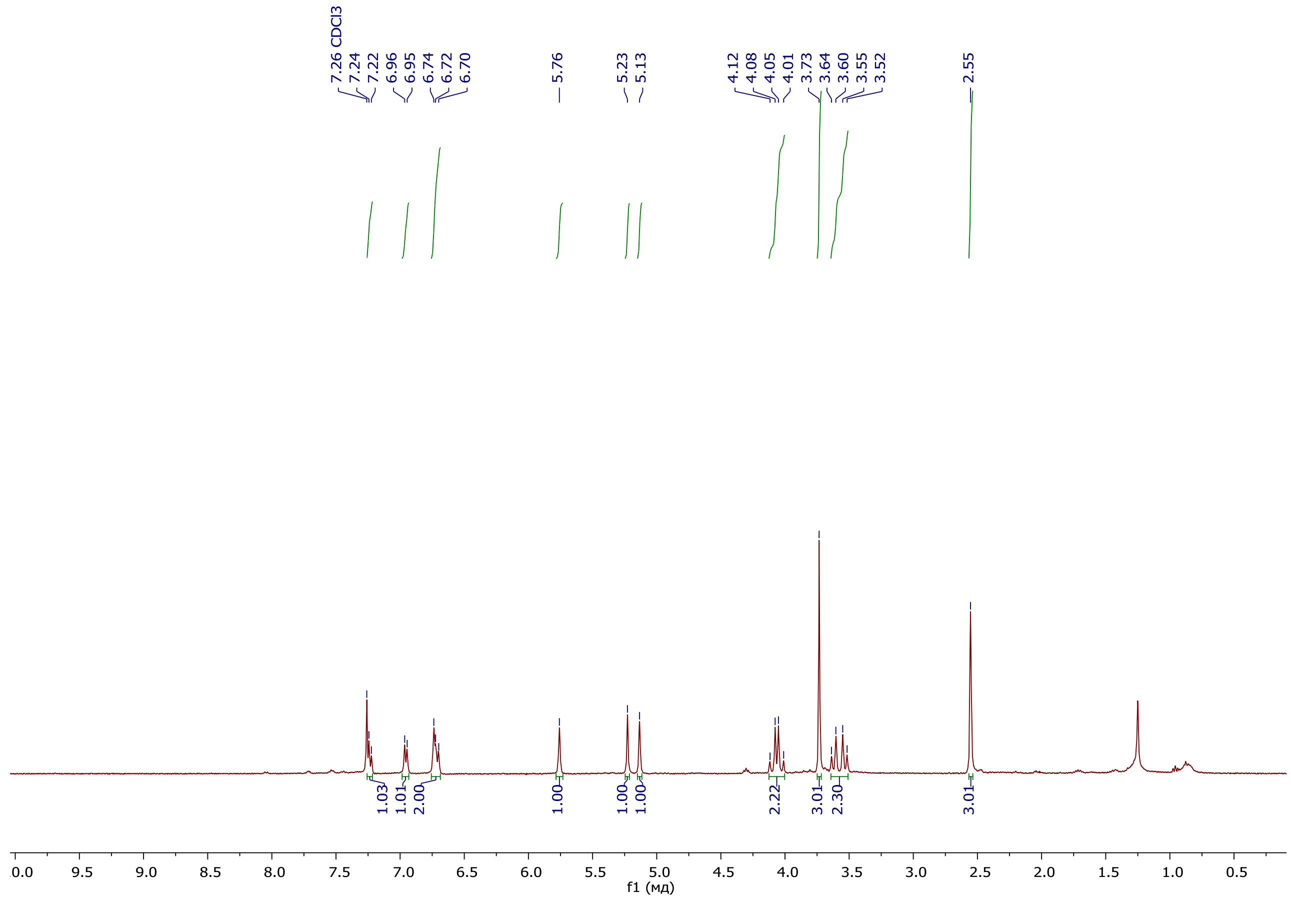

Figure S41. ^{1}H NMR of **3i** in $CDCl_3$

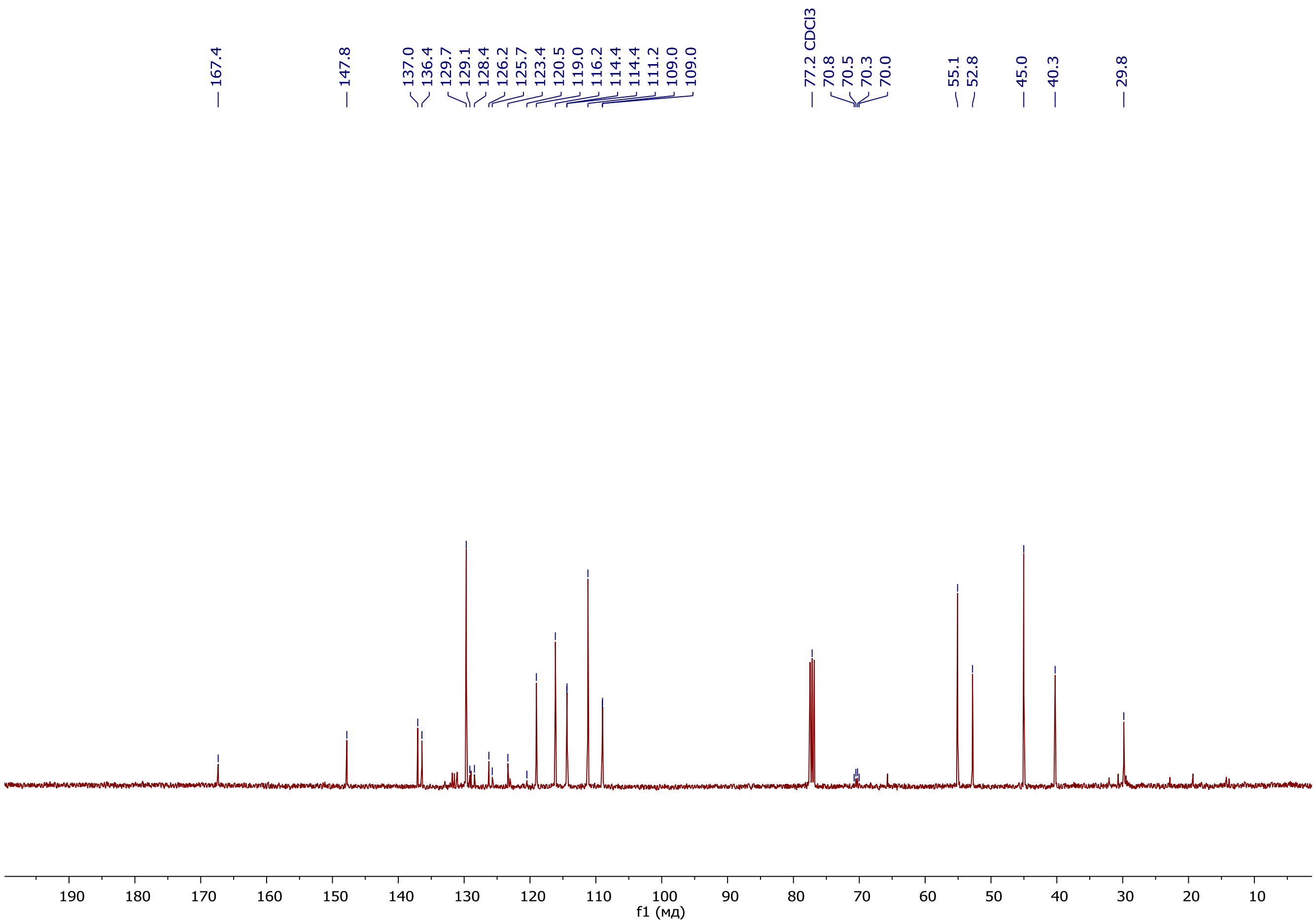

Figure S42. ^{13}C NMR of **3i** in $CDCl_3$

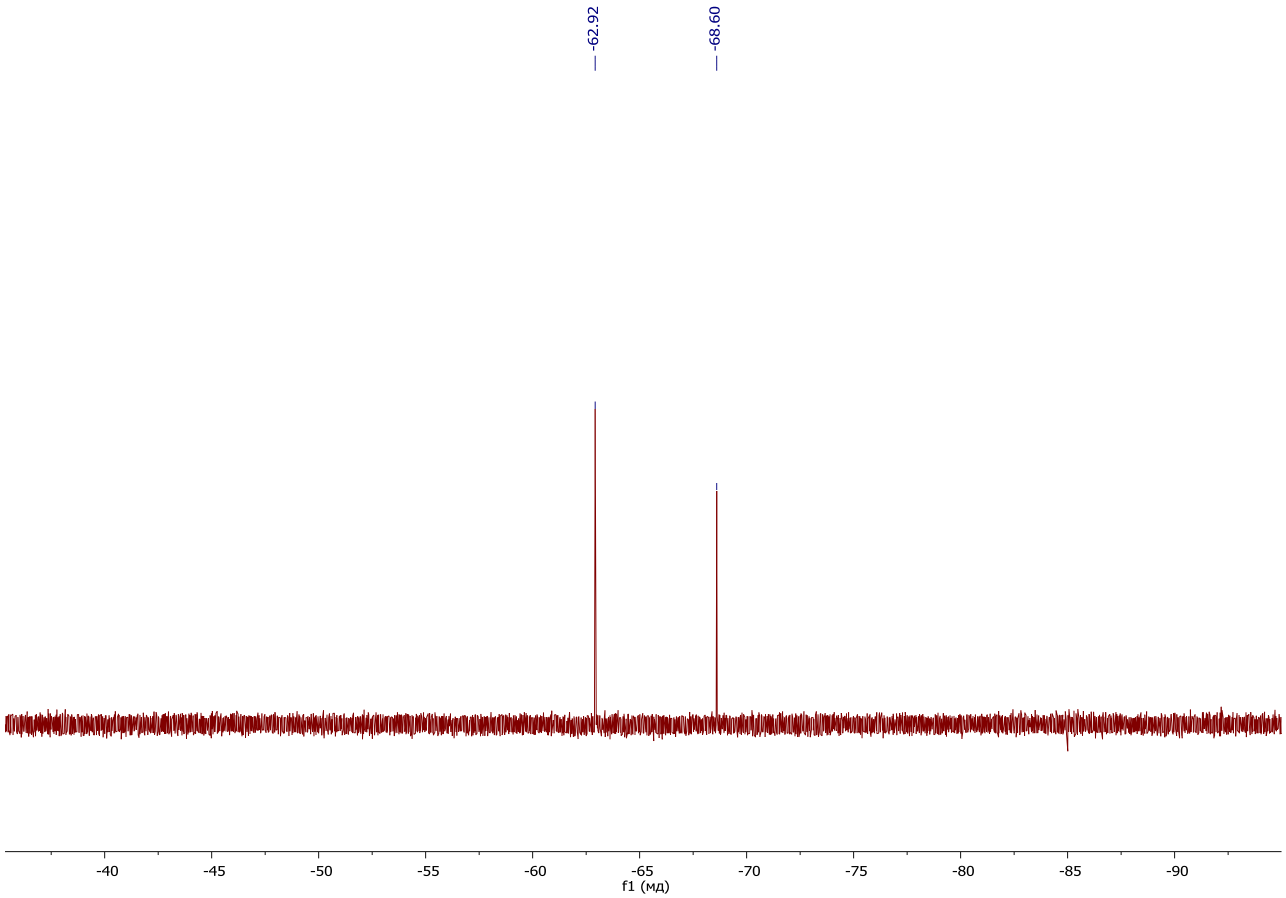

Figure S43. ^{19}F NMR of **3i** in $CDCl_3$

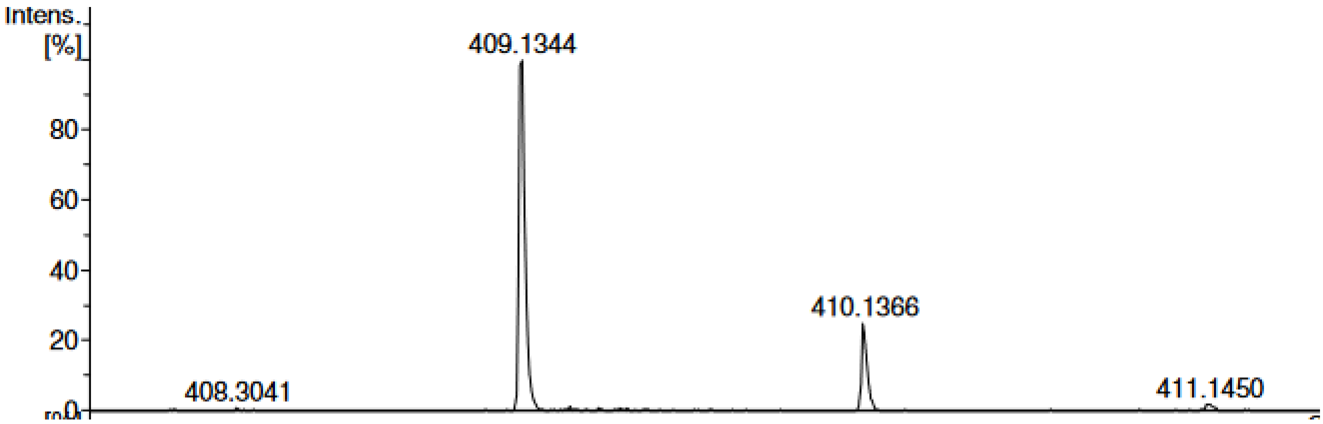

Figure S44. HRMS of **3i**

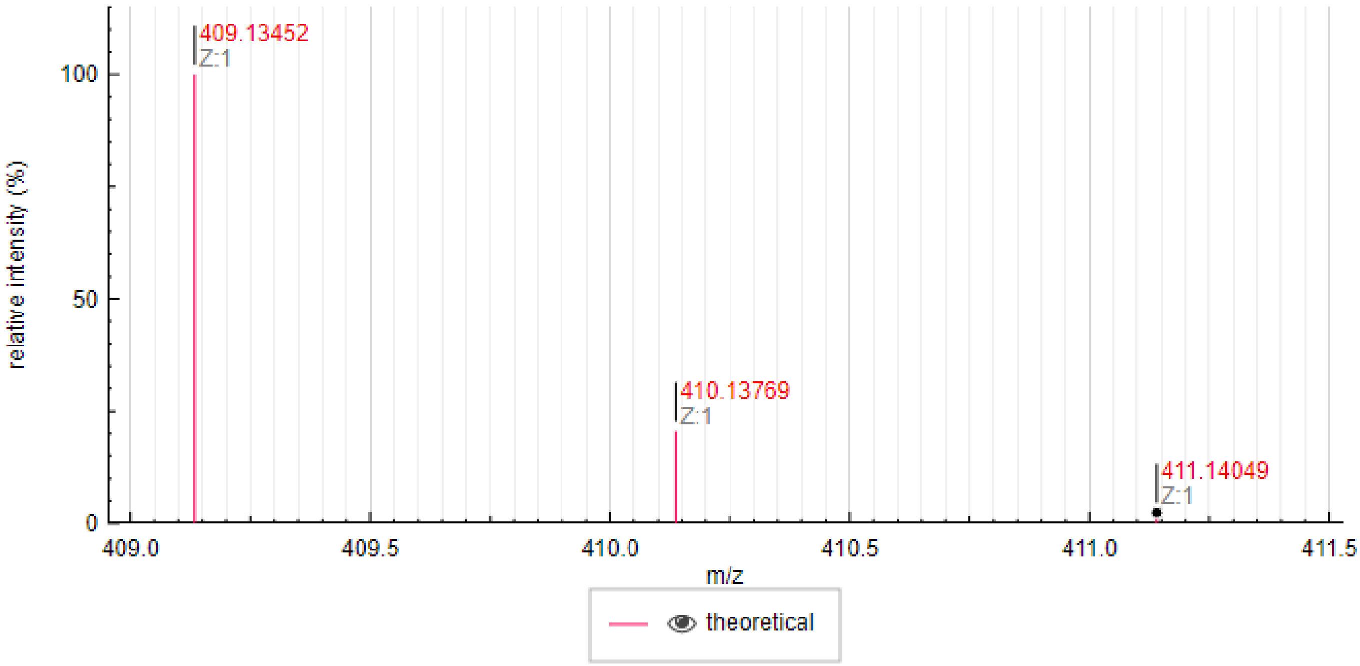

Figure S45. Theoretical HRMS $[M+H]^+$ of **3i**

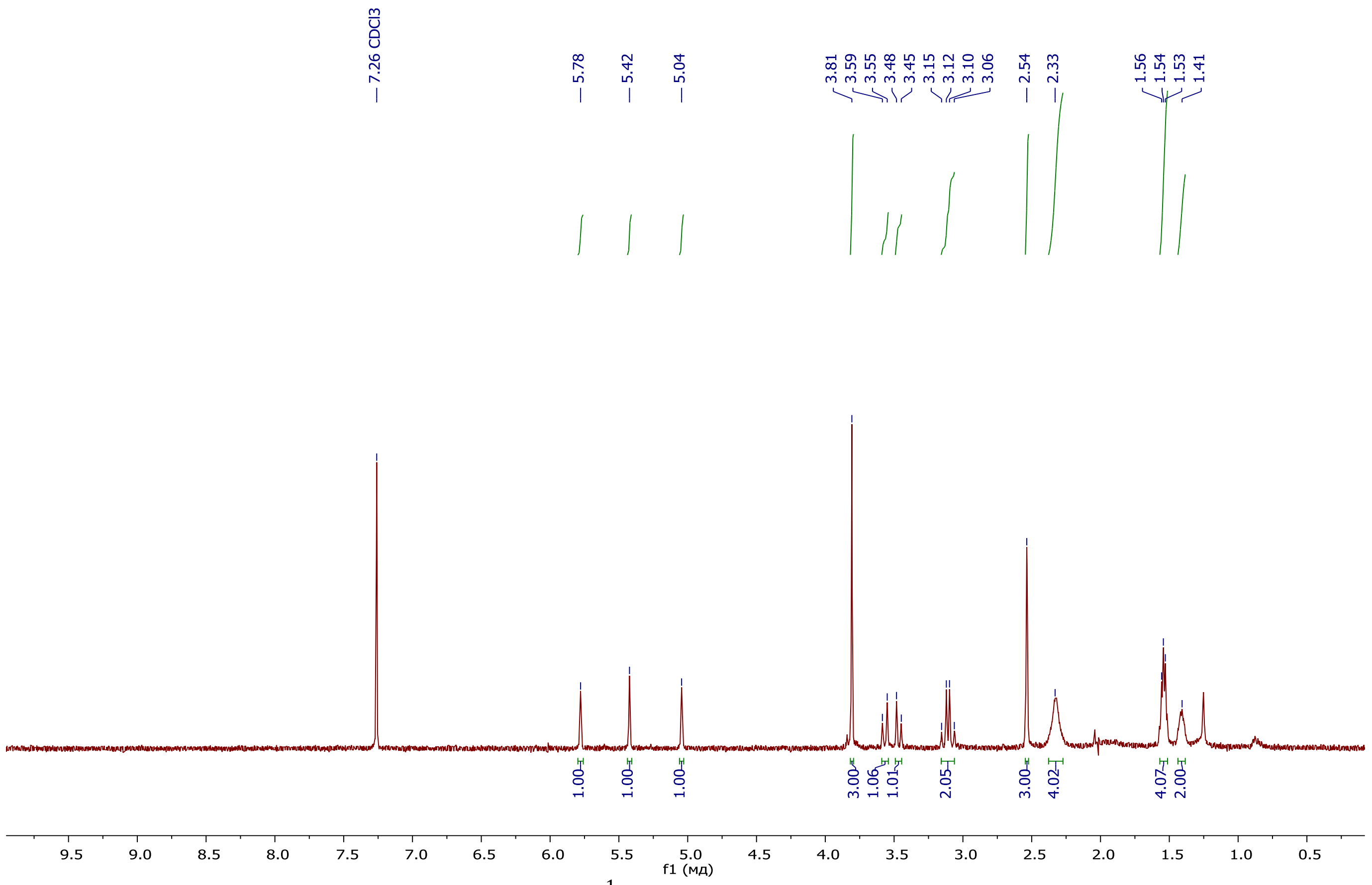

Figure S46. ^{1}H NMR of **3j** in $CDCl_3$

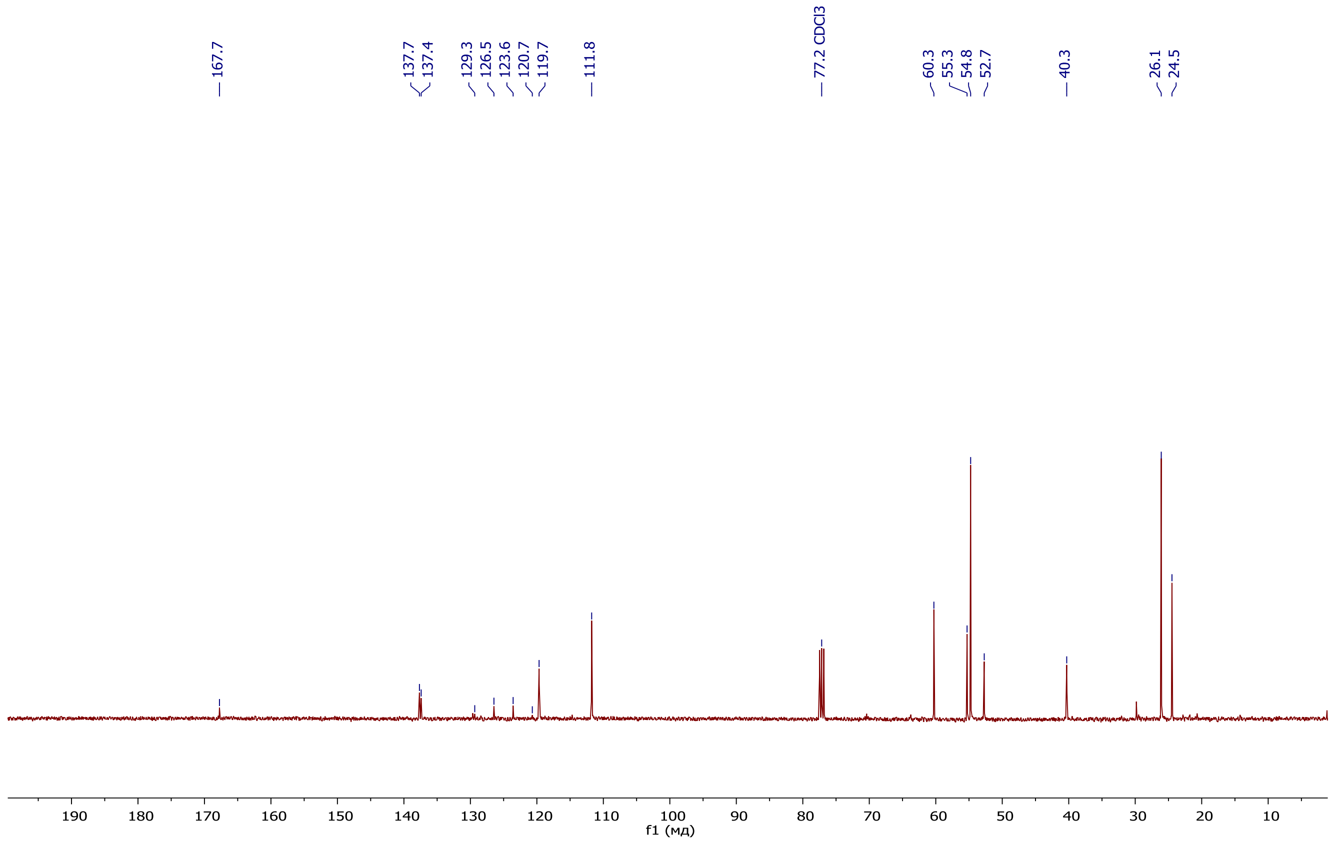

Figure S47. ^{13}C NMR of **3j** in $CDCl_3$

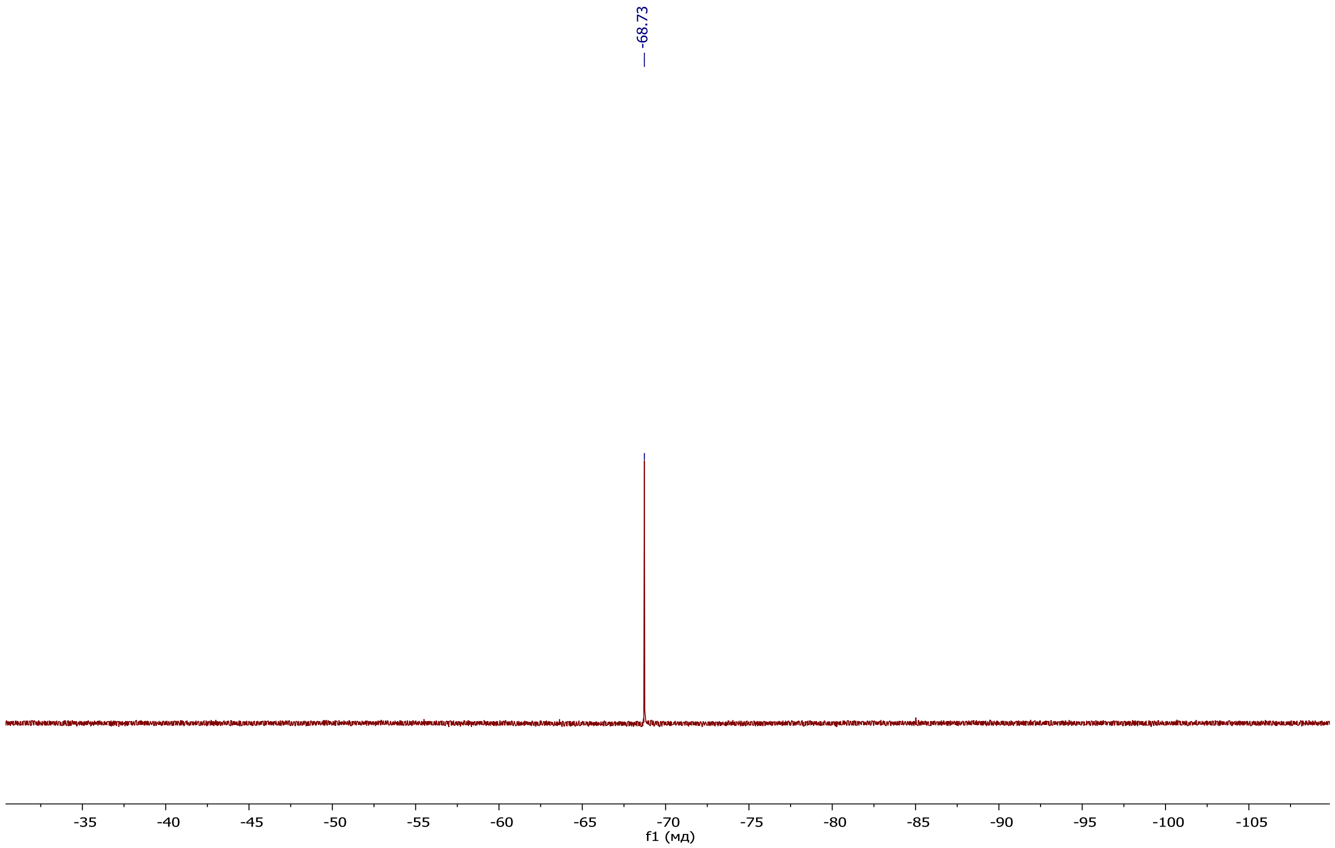

Figure S48. ^{19}F NMR of **3j** in $CDCl_3$

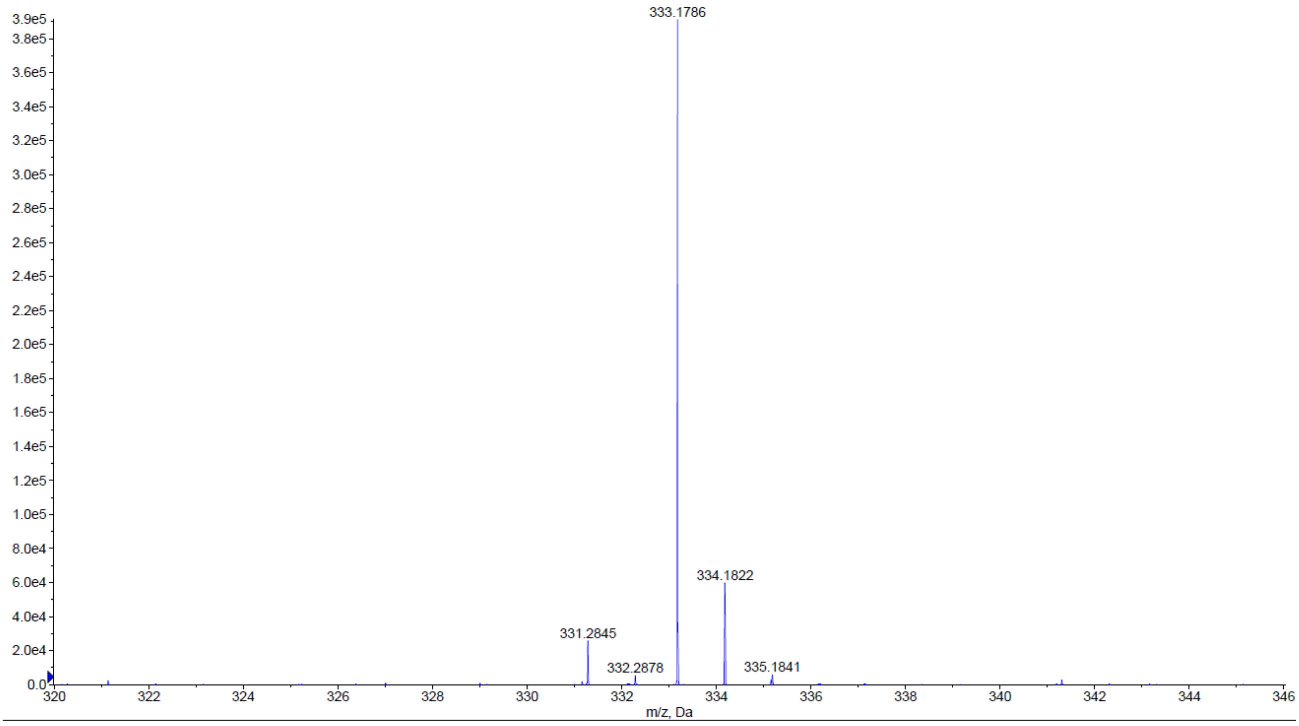

Figure S49. HRMS of **3j**

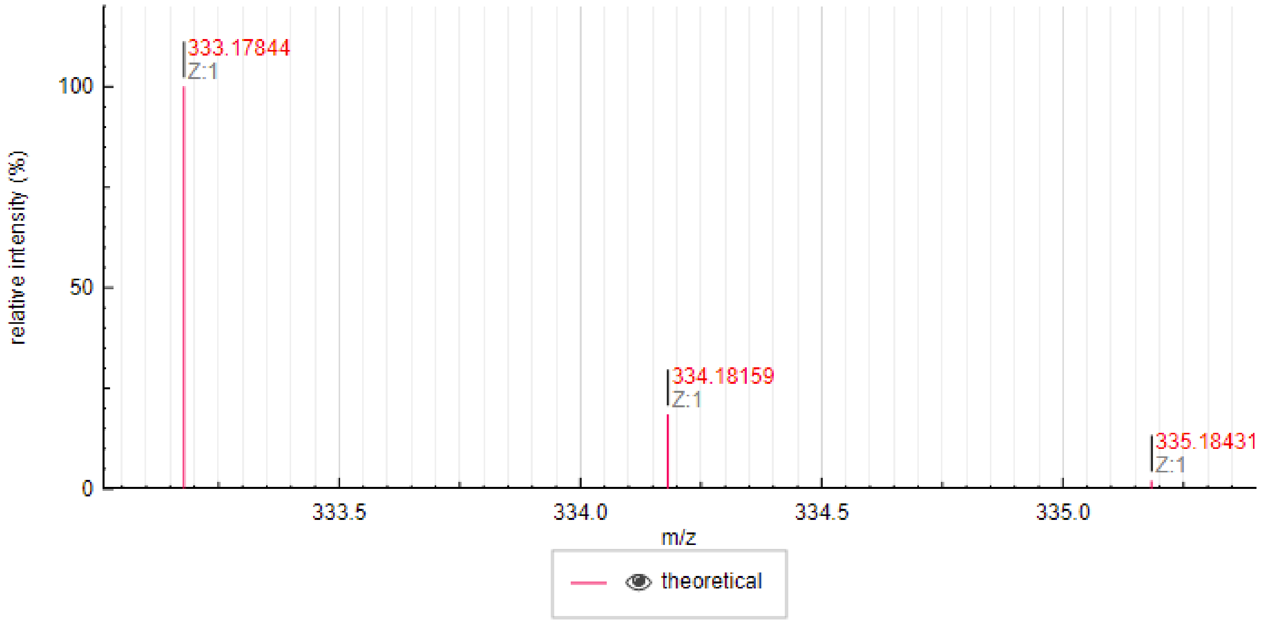

Figure S50. Theoretical HRMS $[M+H]^+$ of **3j**

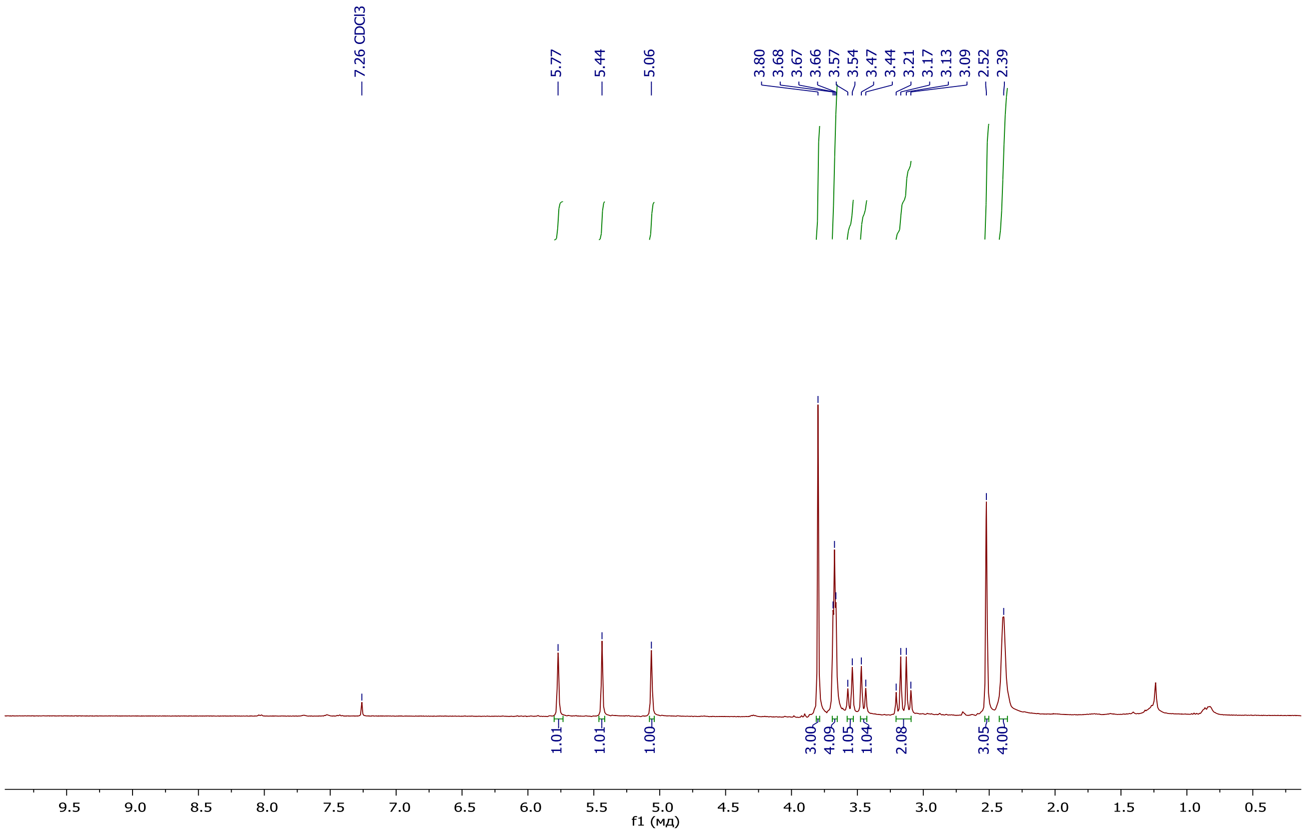

Figure S51. ^{1}H NMR of **3k** in $CDCl_3$

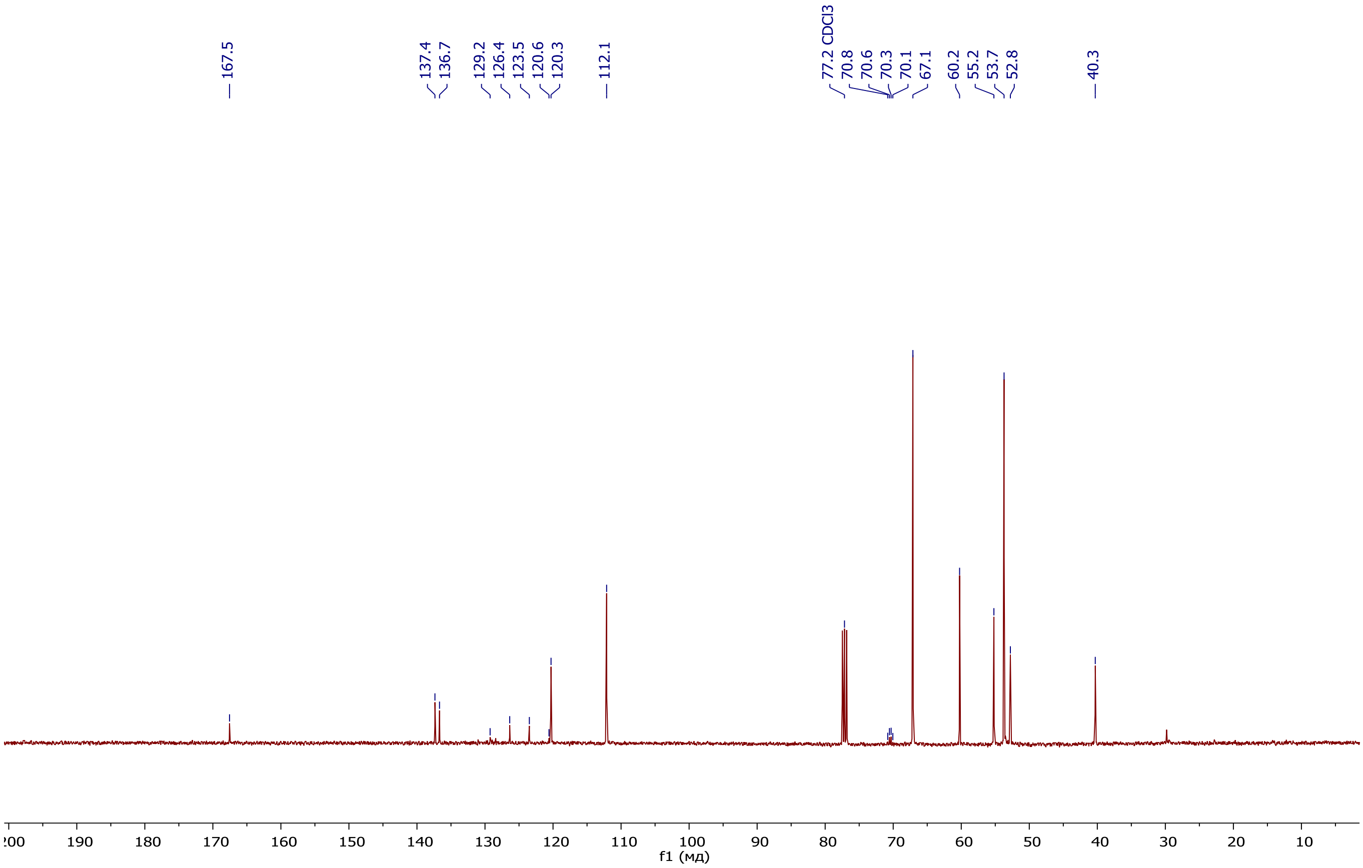

Figure S52. ^{13}C NMR of **3k** in $CDCl_3$

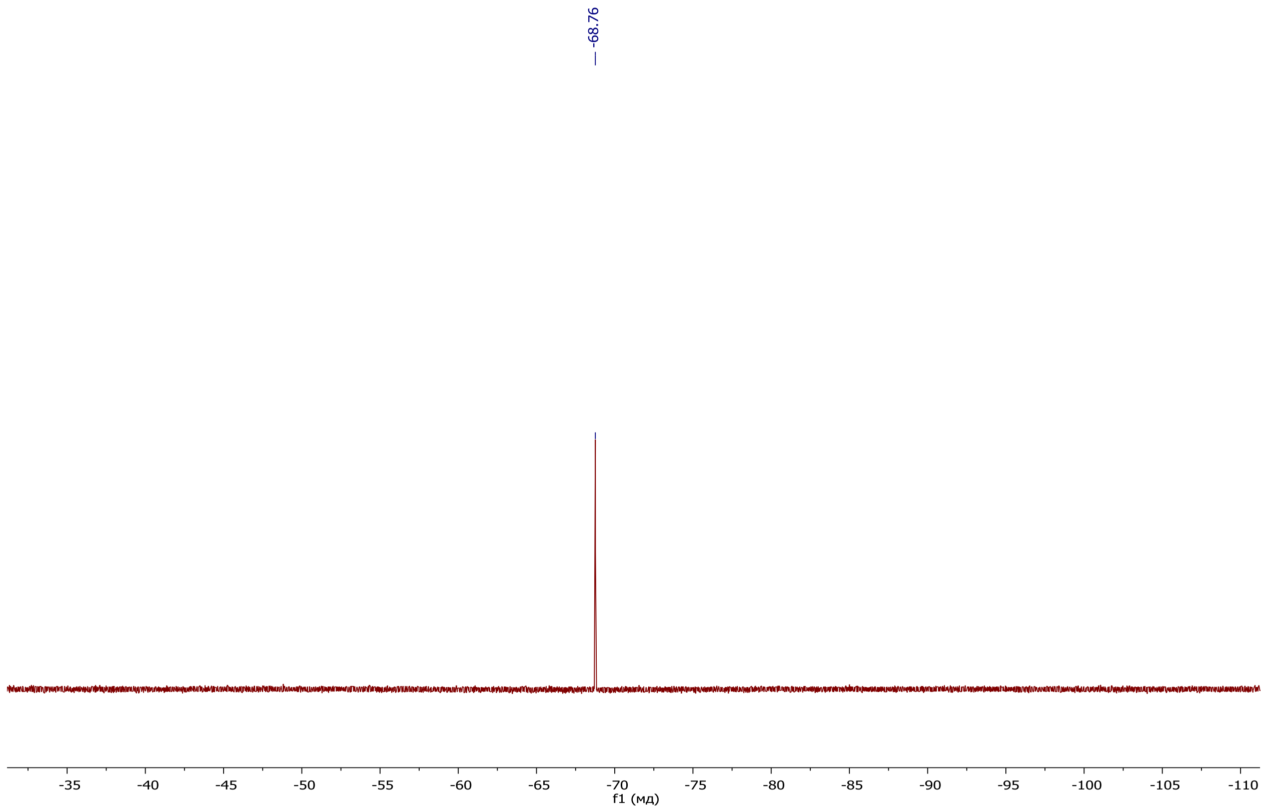

Figure S53. ^{19}F NMR of **3k** in $CDCl_3$

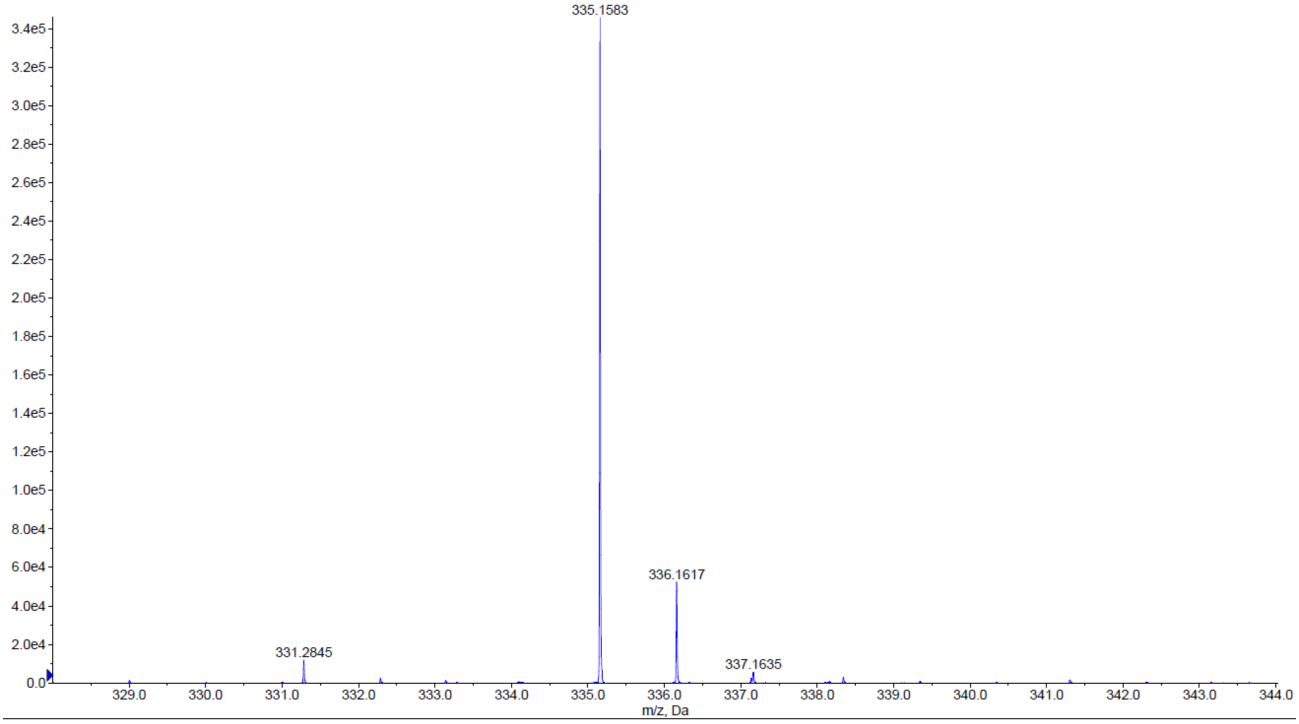

Figure S54. HRMS of **3k**

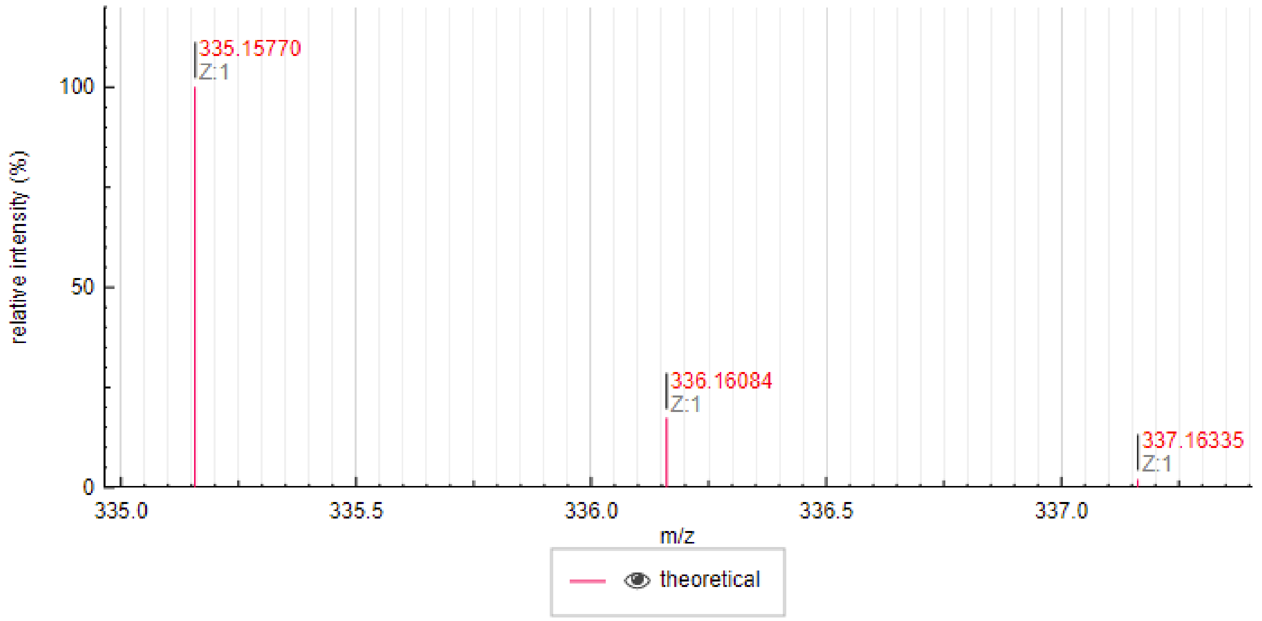

Figure S55. Theoretical HRMS $[M+H]^+$ of **3k**

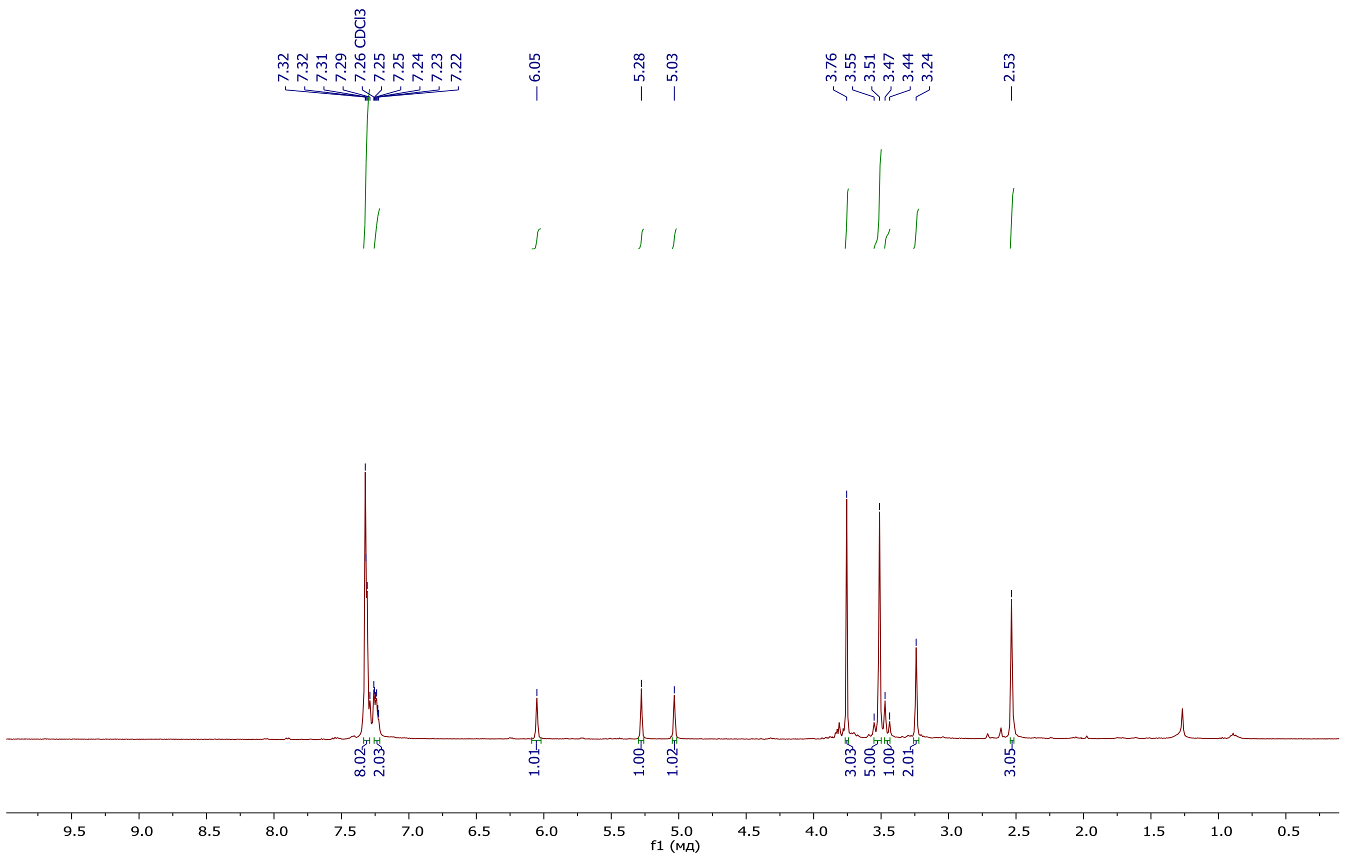

Figure S56. ^{1}H NMR of **3l** in $CDCl_3$

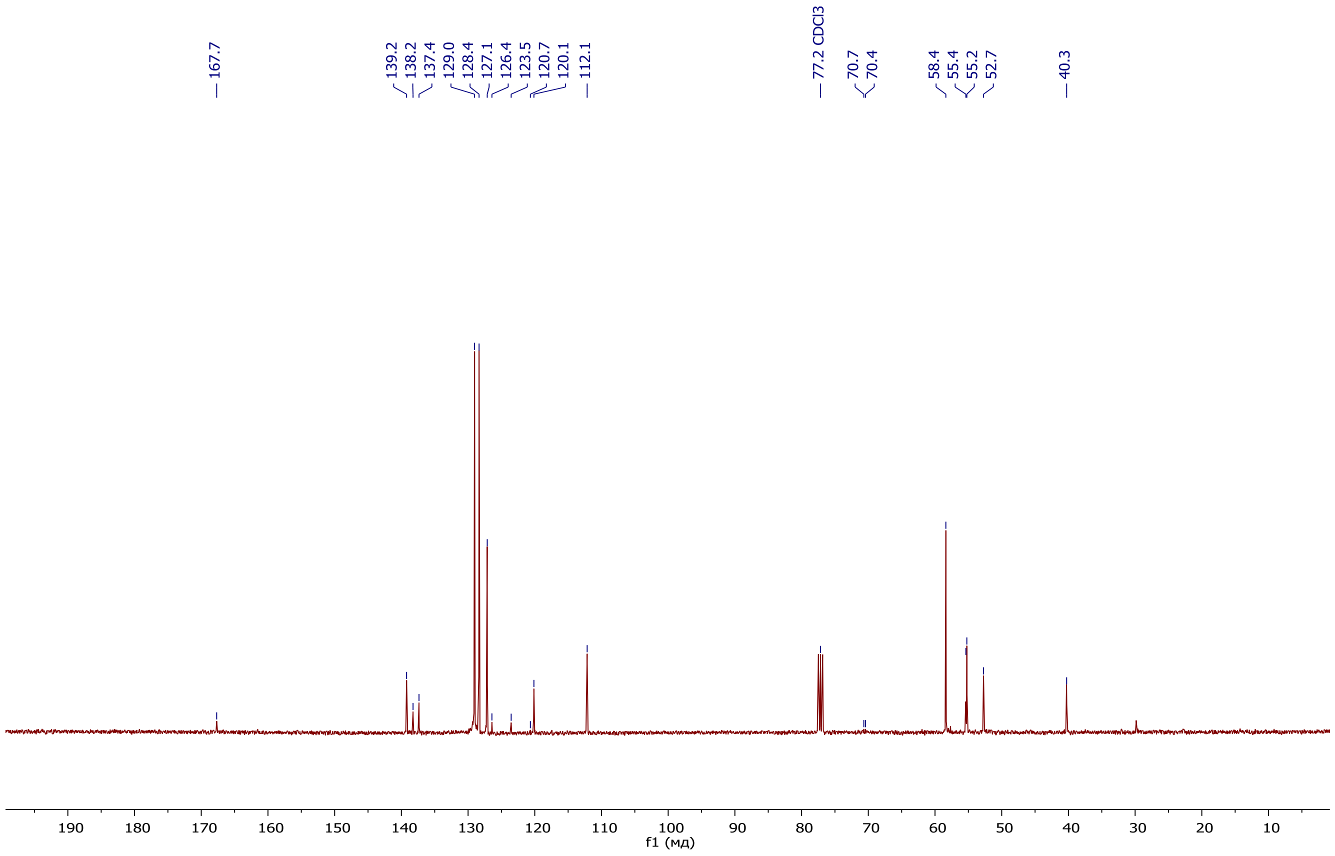

Figure S57. ^{13}C NMR of **3l** in $CDCl_3$

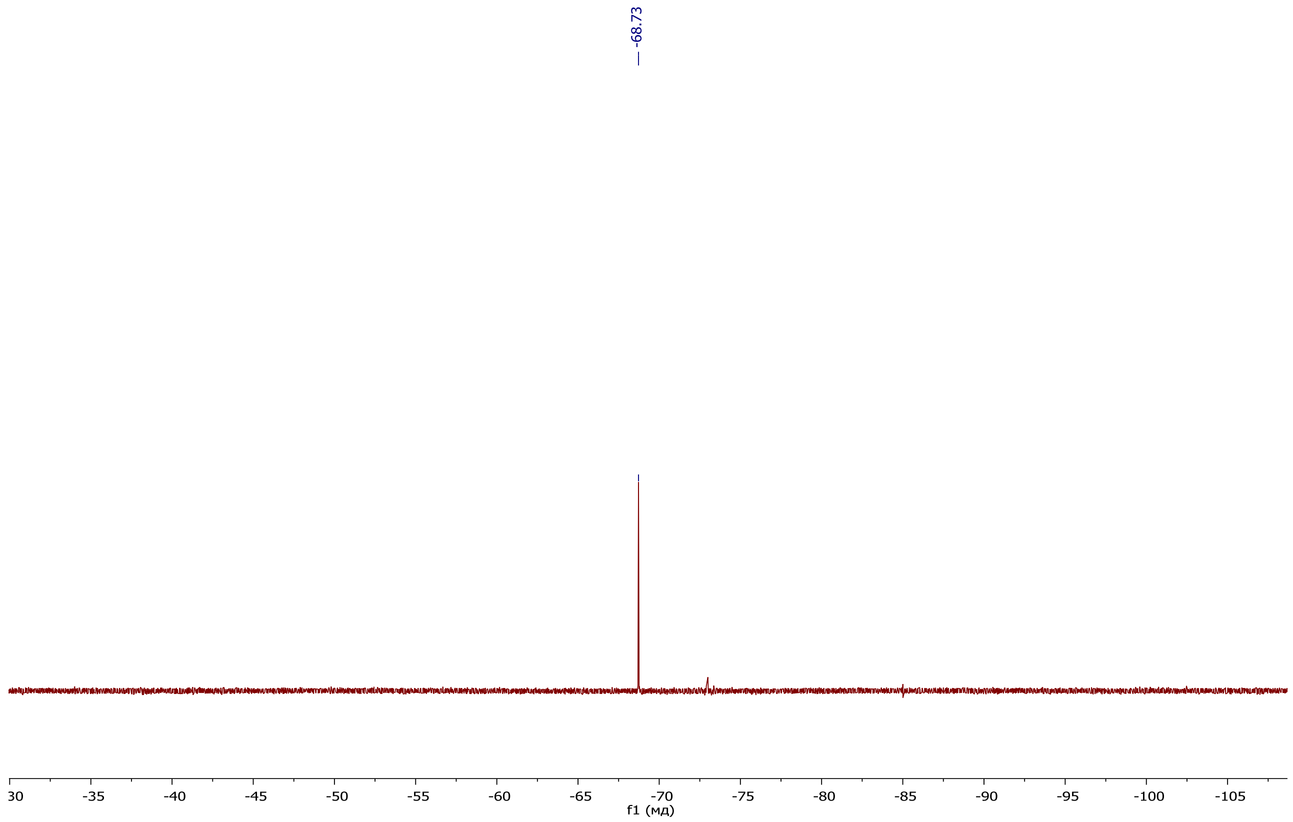

Figure S58. ^{19}F NMR of **3l** in $CDCl_3$

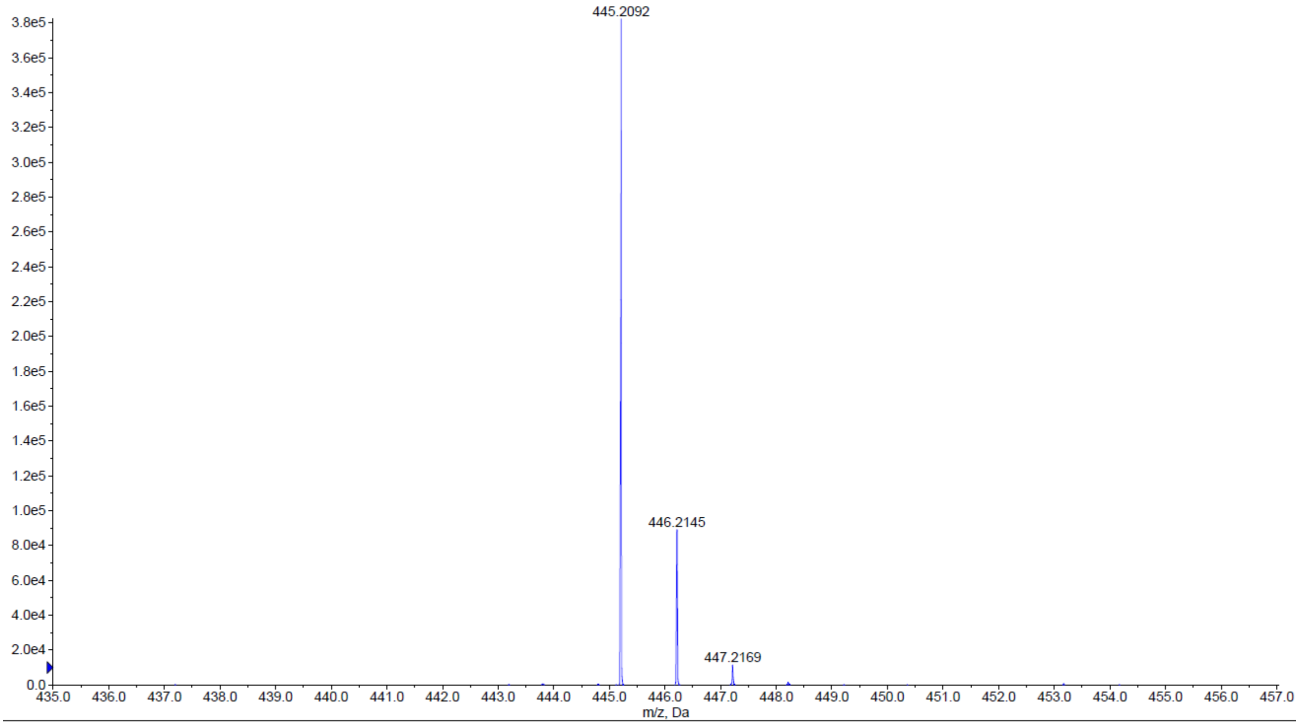

Figure S59. HRMS of **3l**

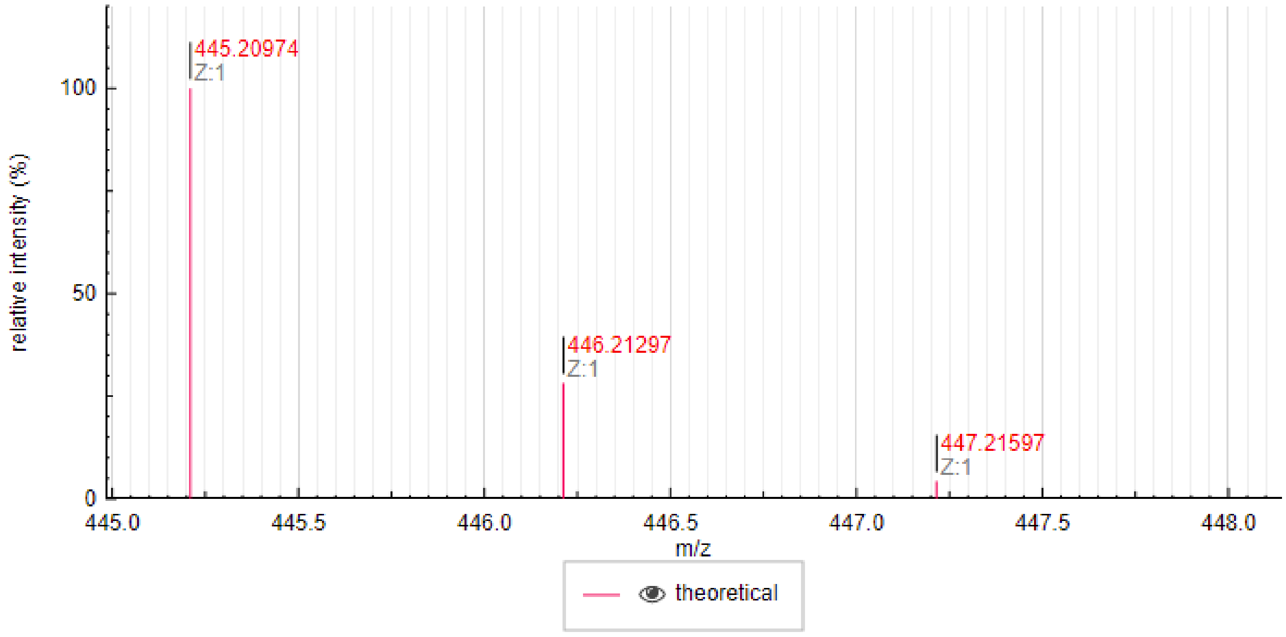

Figure S60. Theoretical HRMS $[M+H]^+$ of **3l**

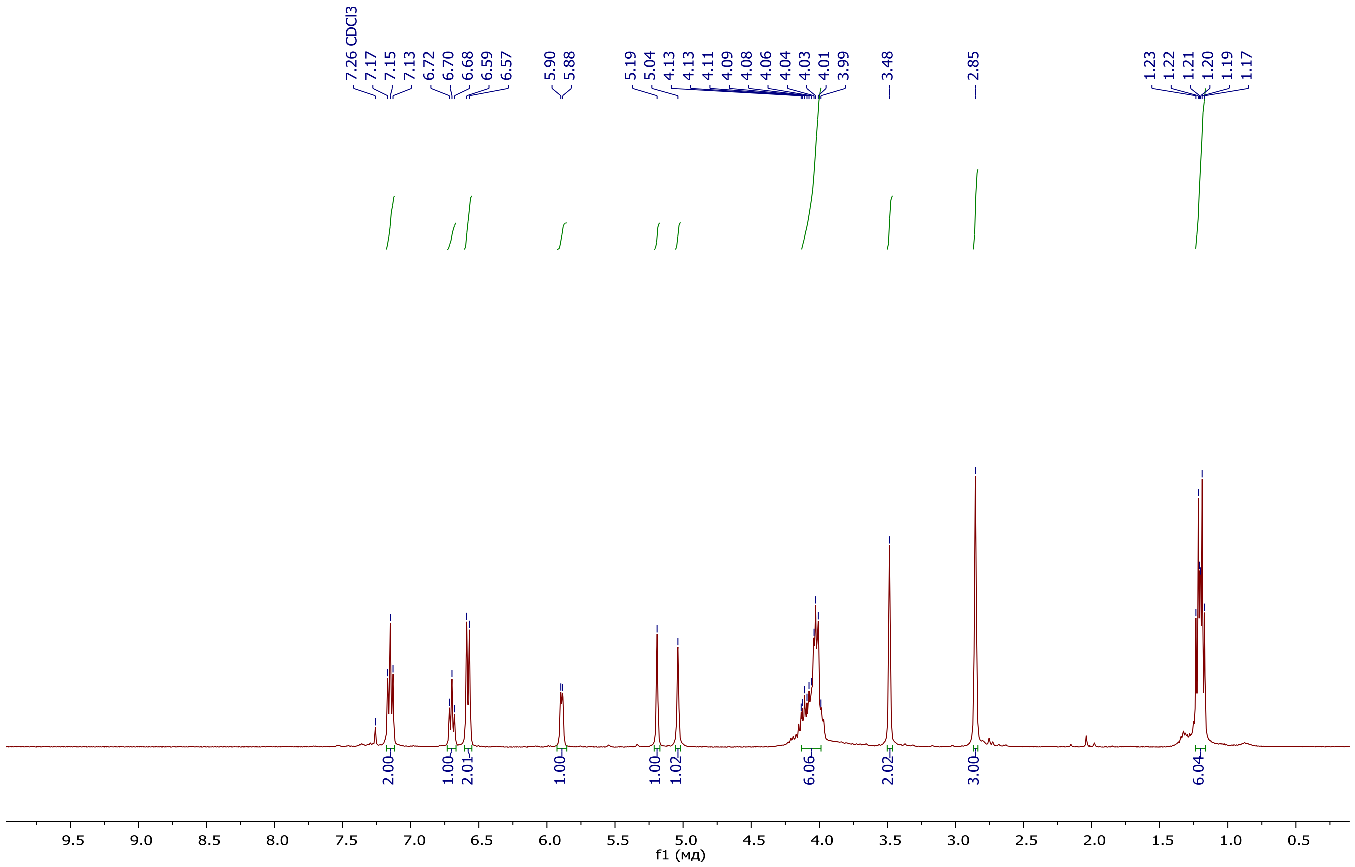

Figure S61. ^{1}H NMR of **4a** in $CDCl_3$

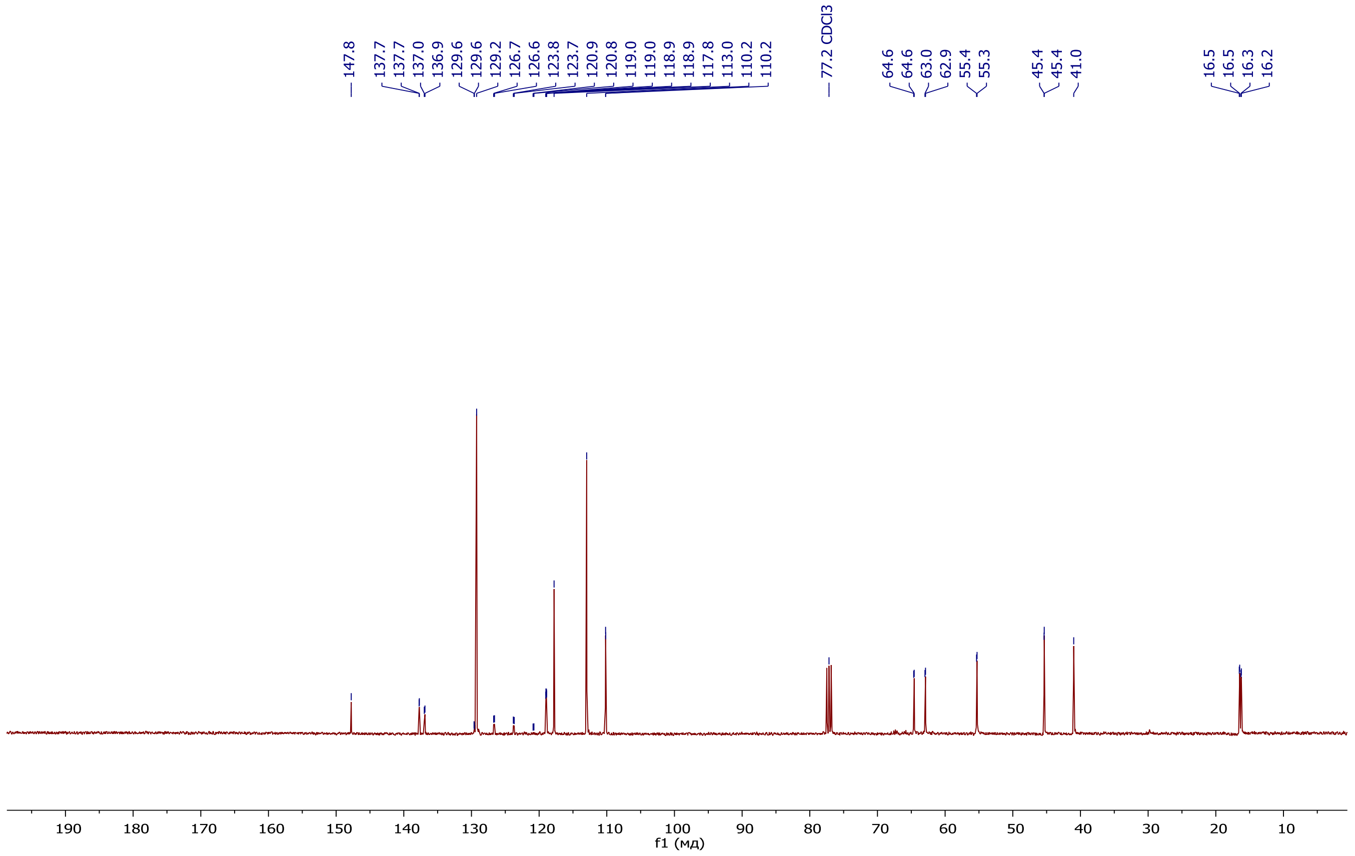

Figure S62. ^{13}C NMR of **4a** in $CDCl_3$

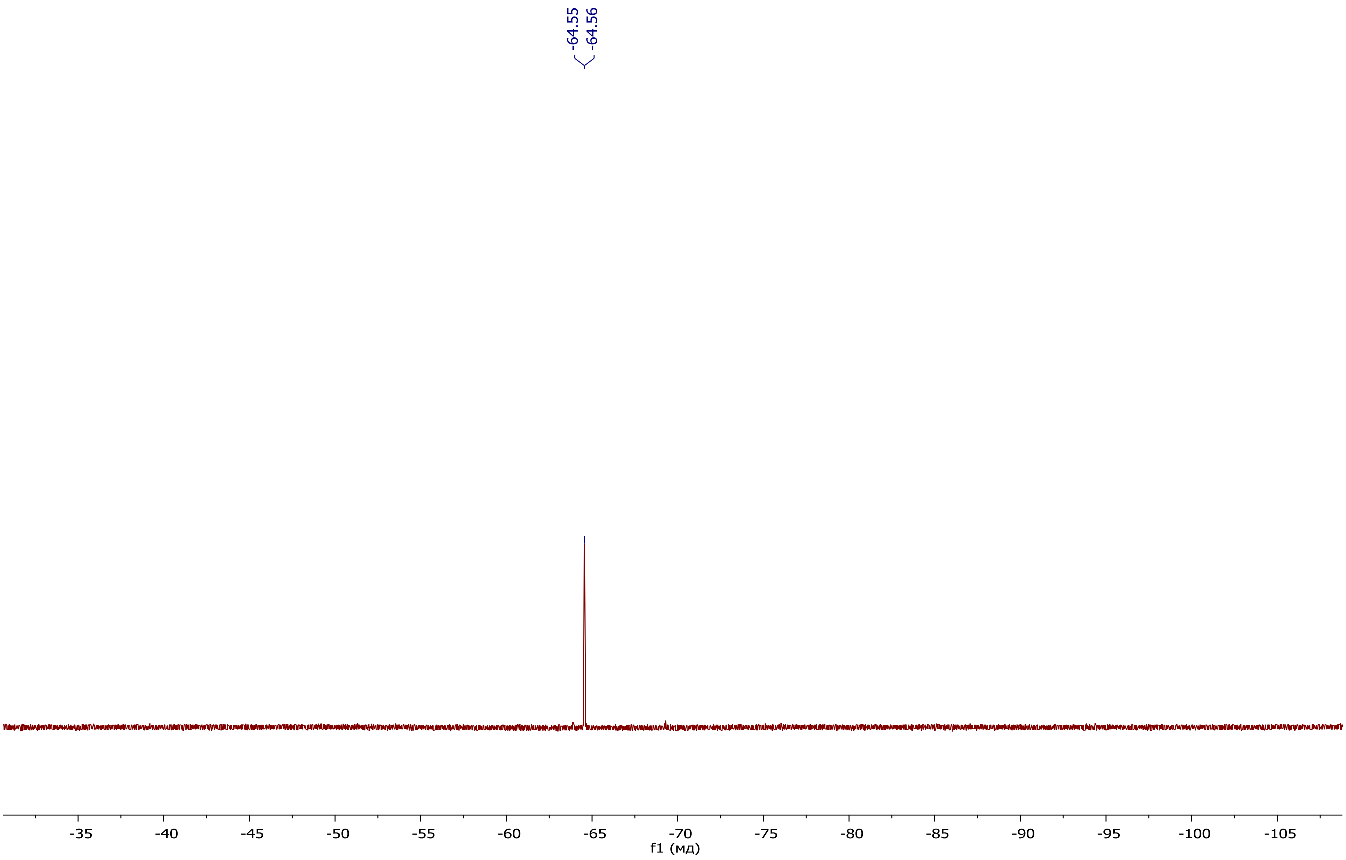

Figure S63. ^{19}F NMR of **4a** in $CDCl_3$

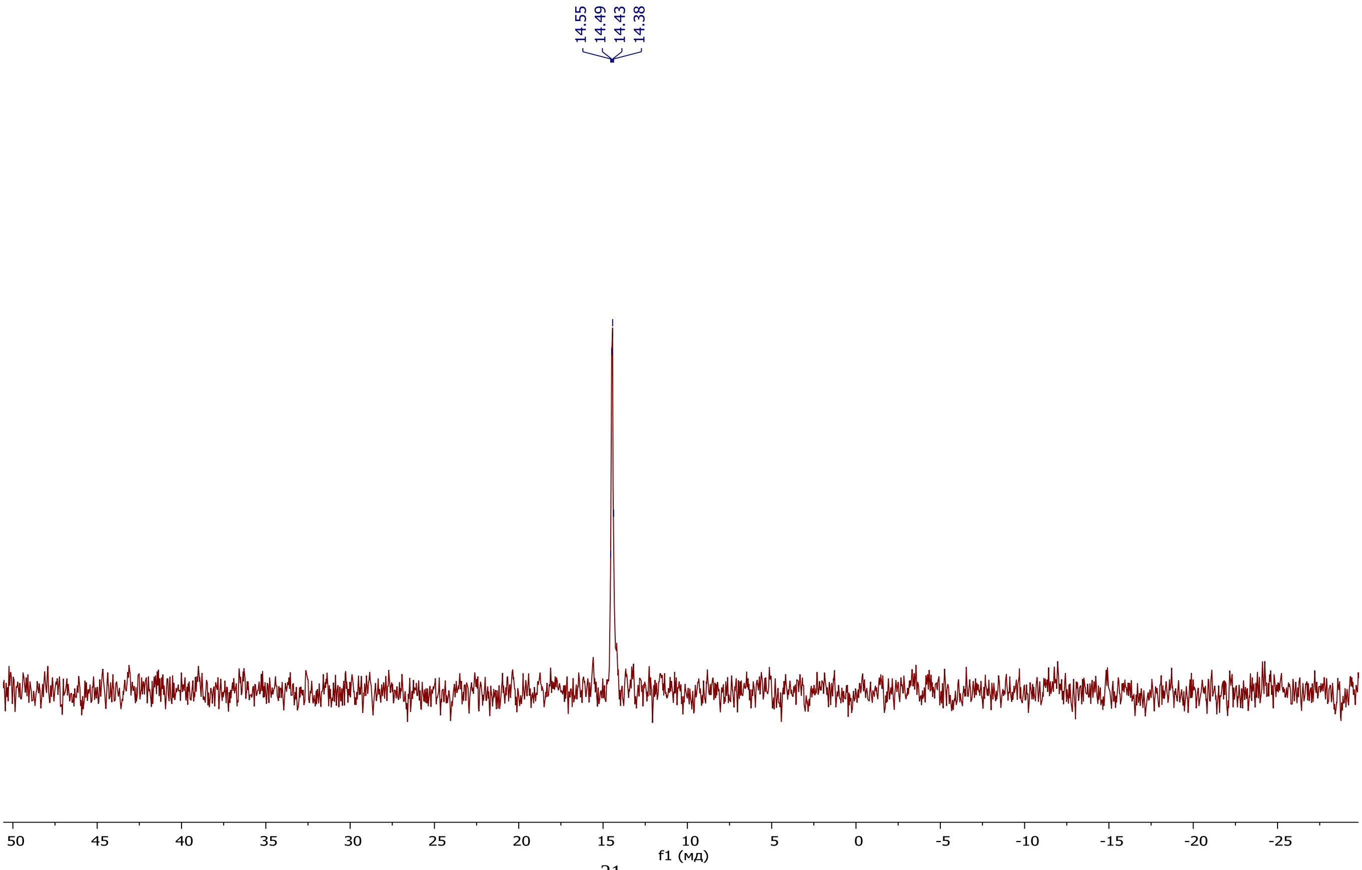

Figure S64. ^{31}P NMR of **4a** in $CDCl_3$

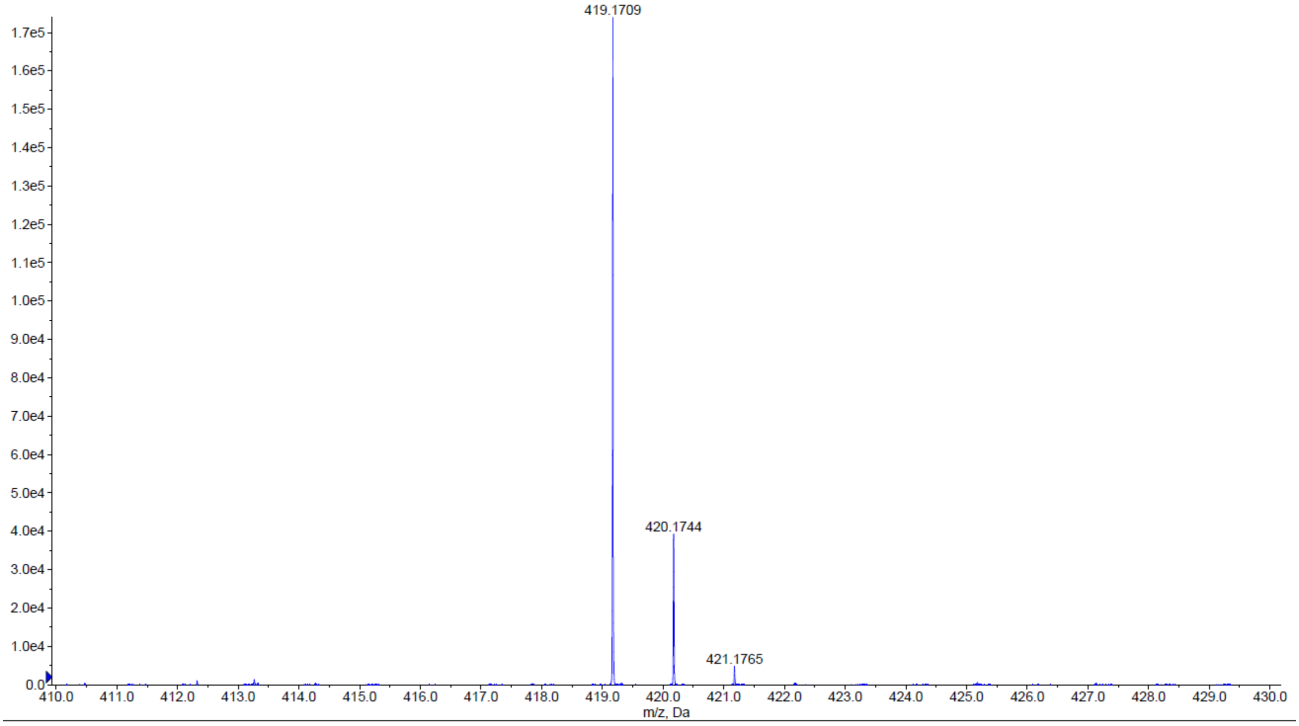

Figure S65. HRMS of **4a**

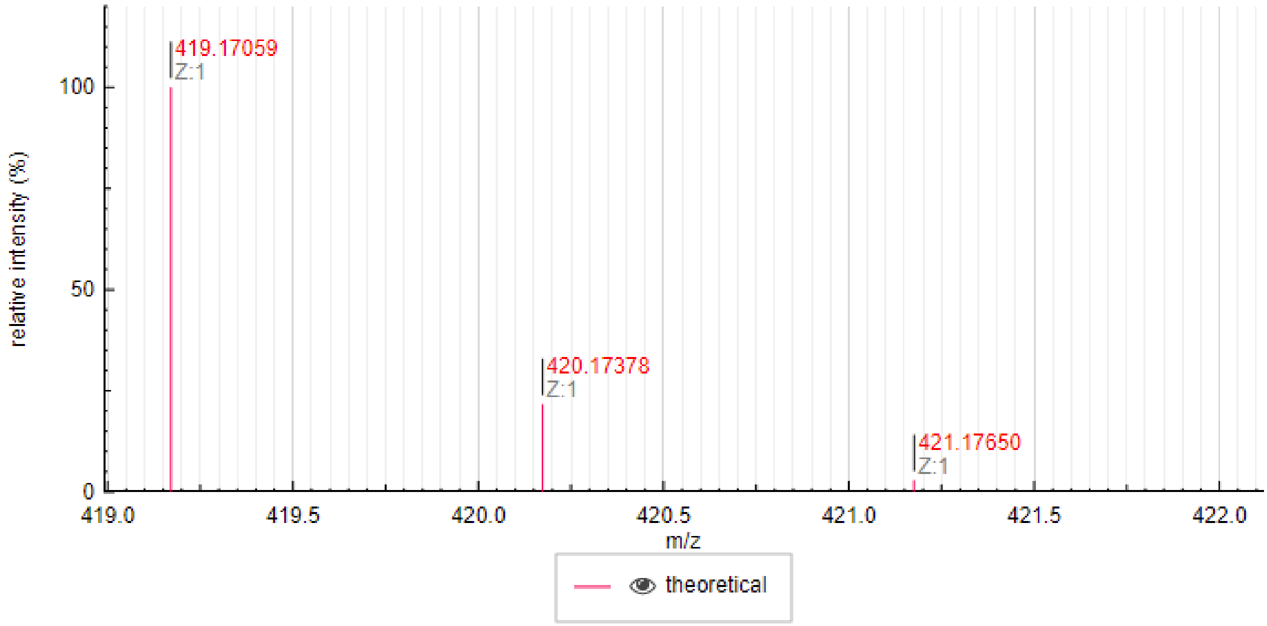

Figure S66. Theoretical HRMS $[M+H]^+$ of **4a**

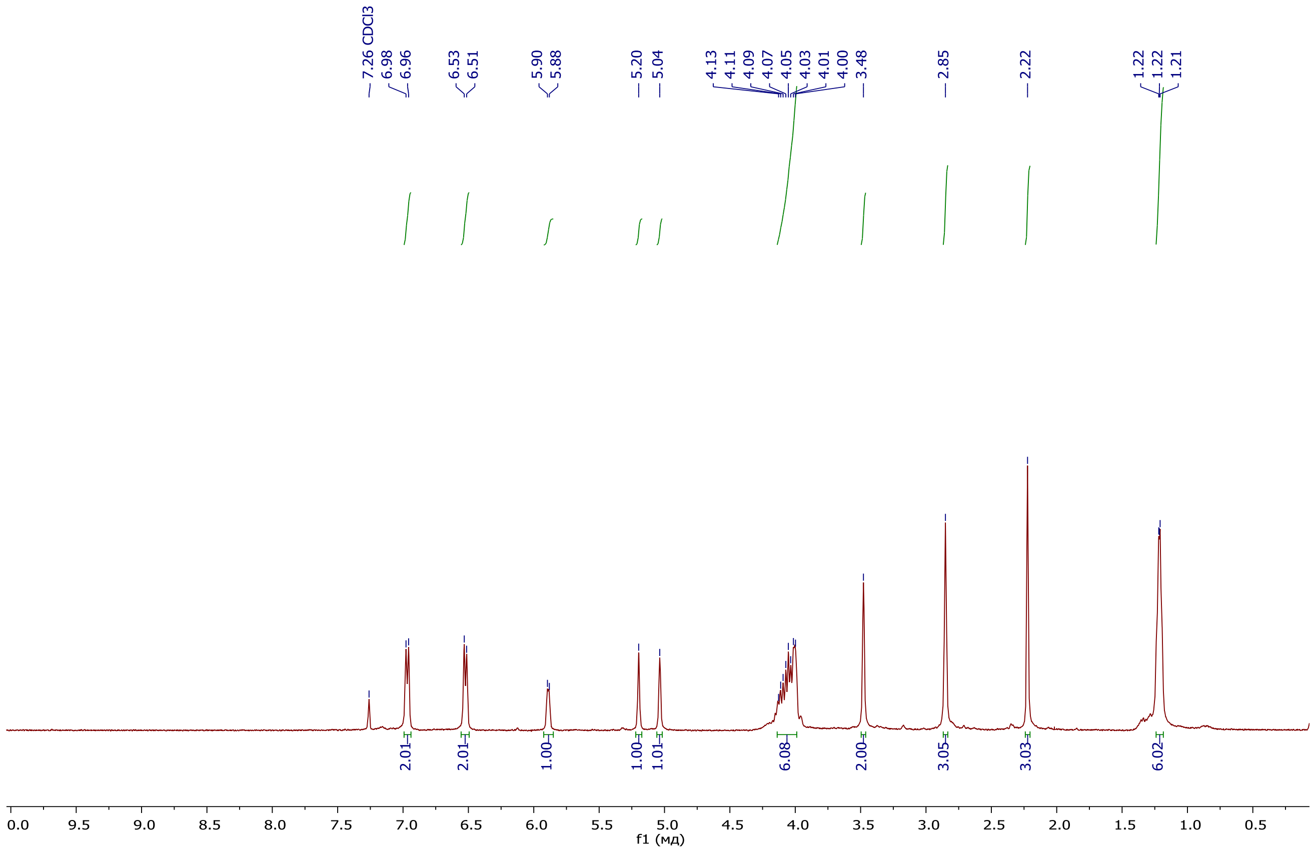

Figure S67. ^{1}H NMR of **4b** in $CDCl_3$

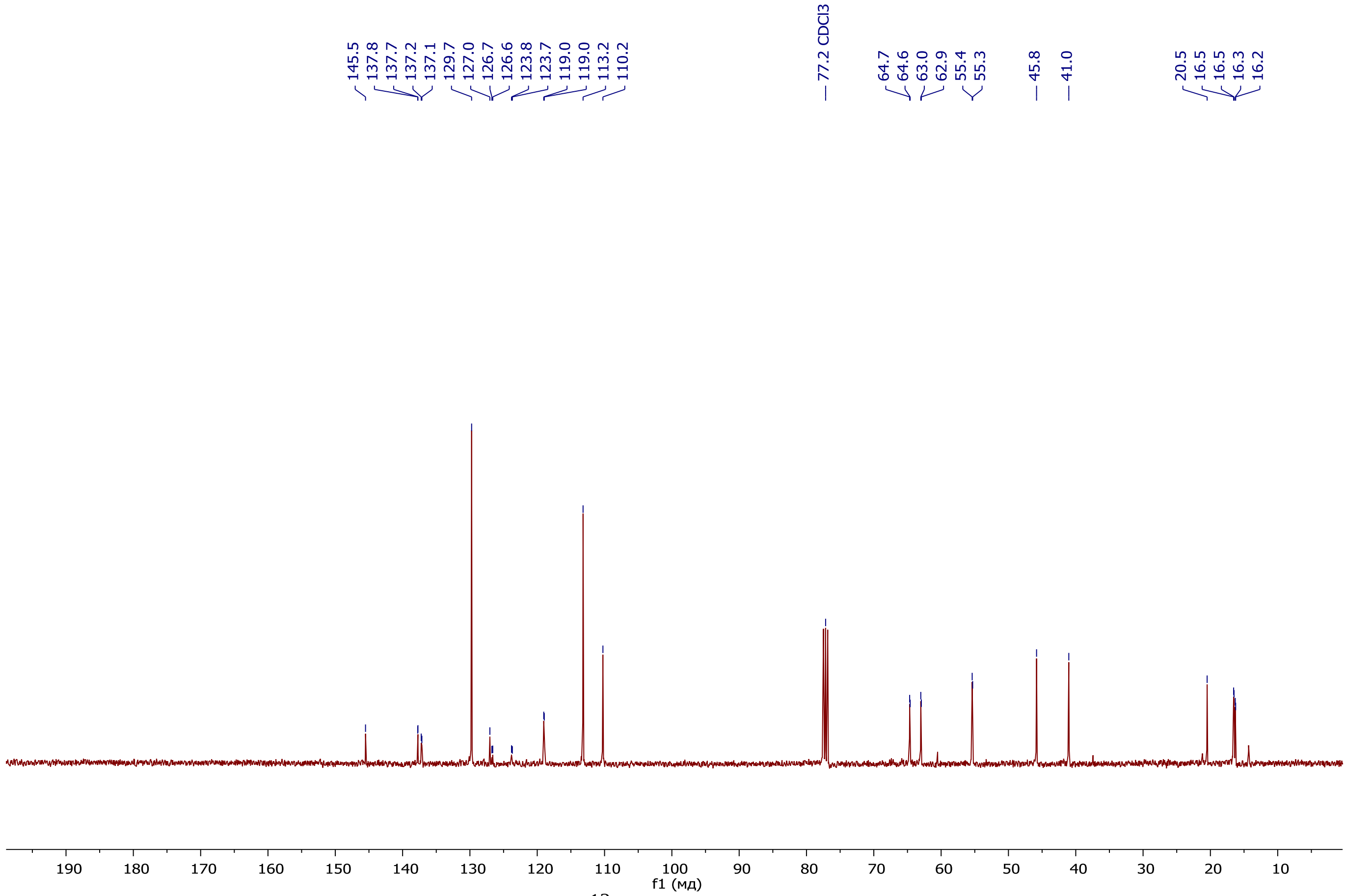

Figure S68. ^{13}C NMR of **4b** in $CDCl_3$

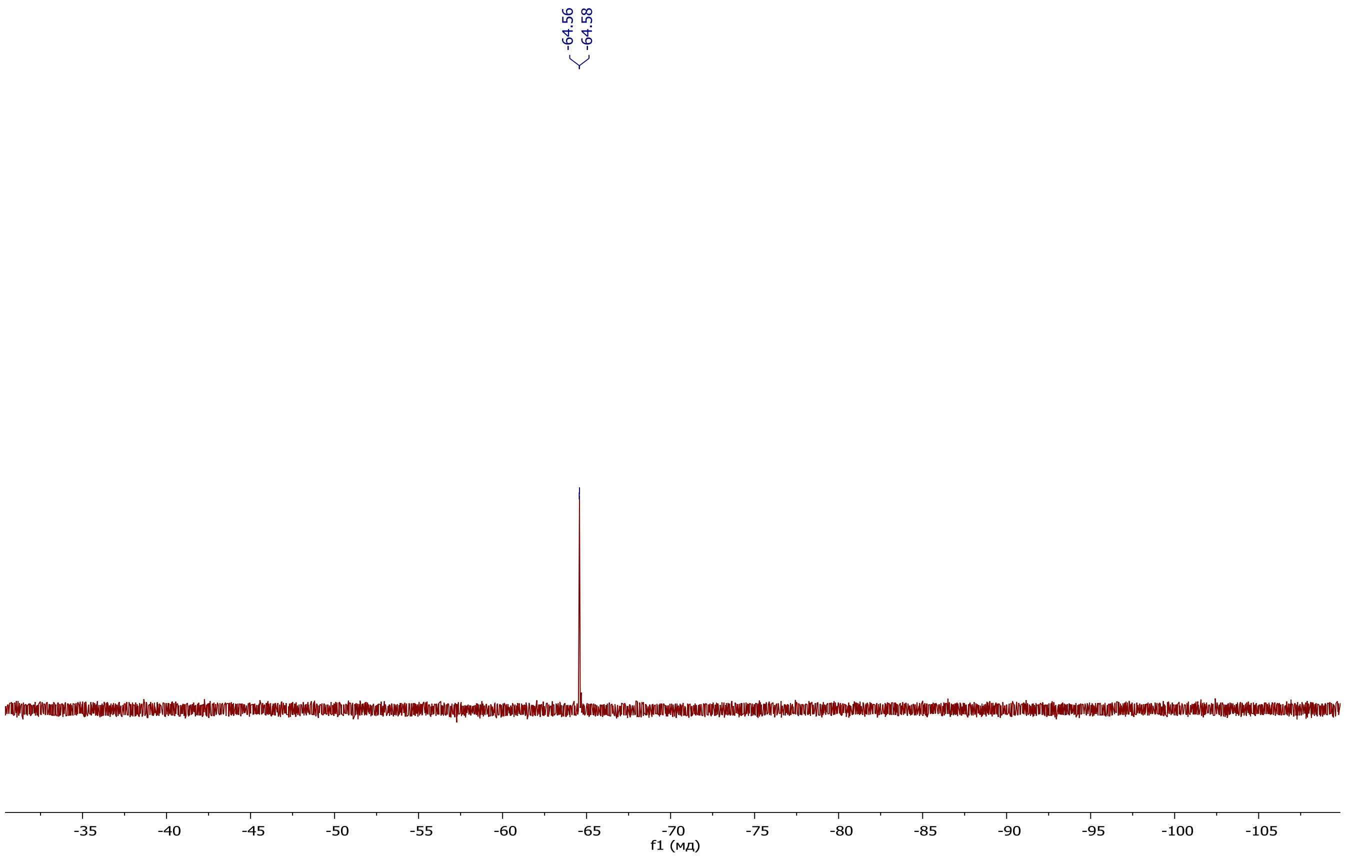

Figure S69. ^{19}F NMR of **4b** in $CDCl_3$

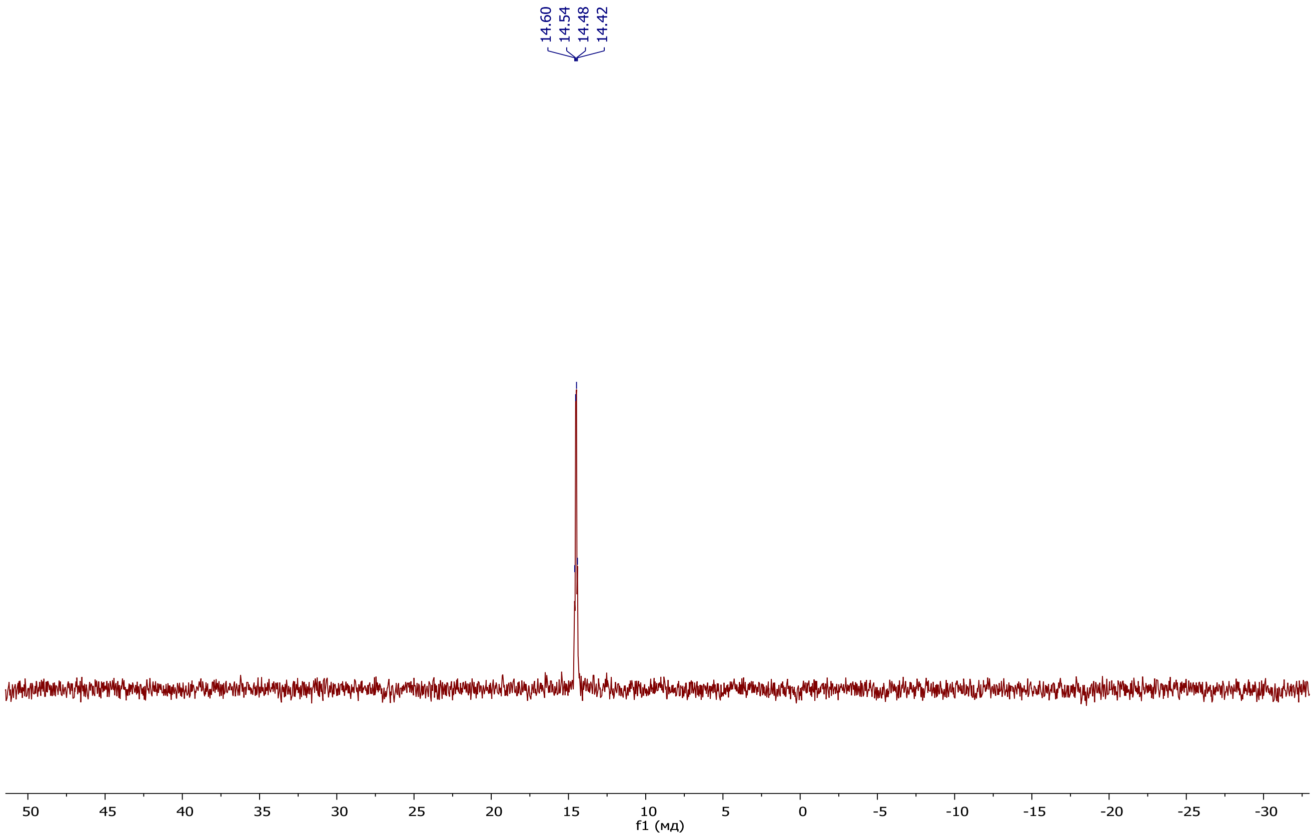

Figure S70. ^{31}P NMR of **4b** in $CDCl_3$

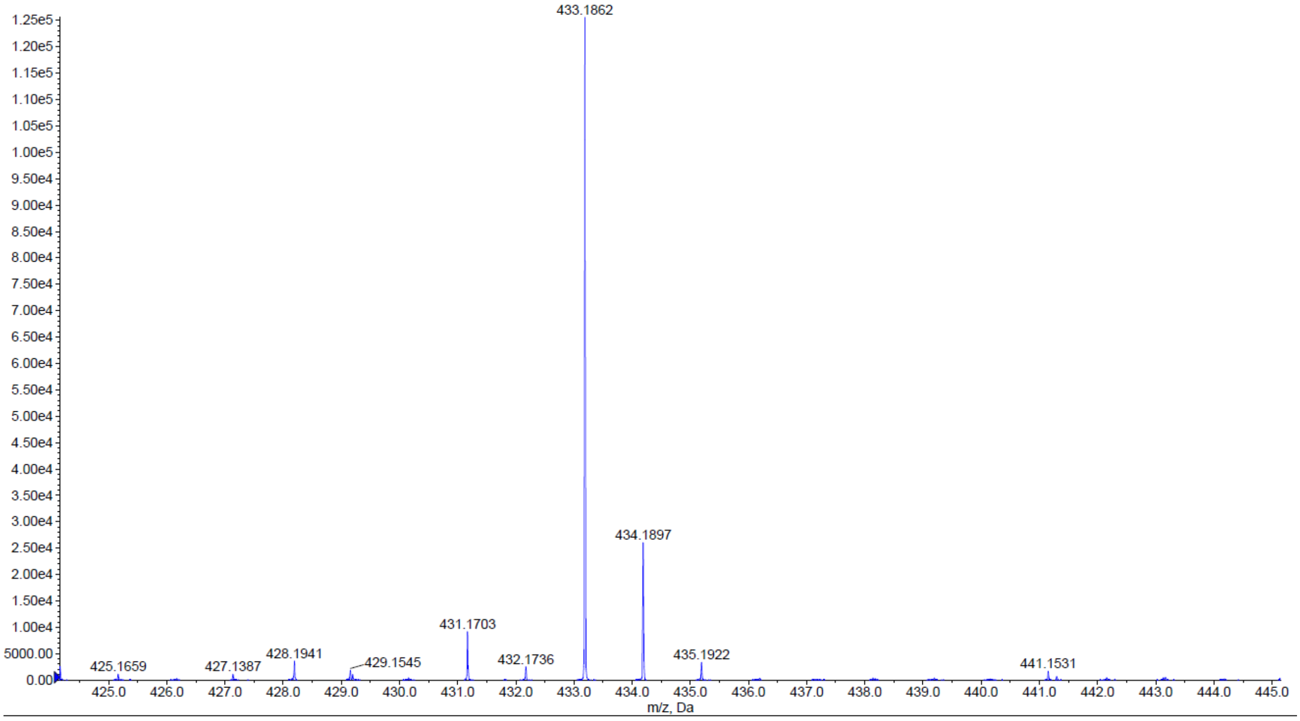

Figure S71. HRMS of **4b**

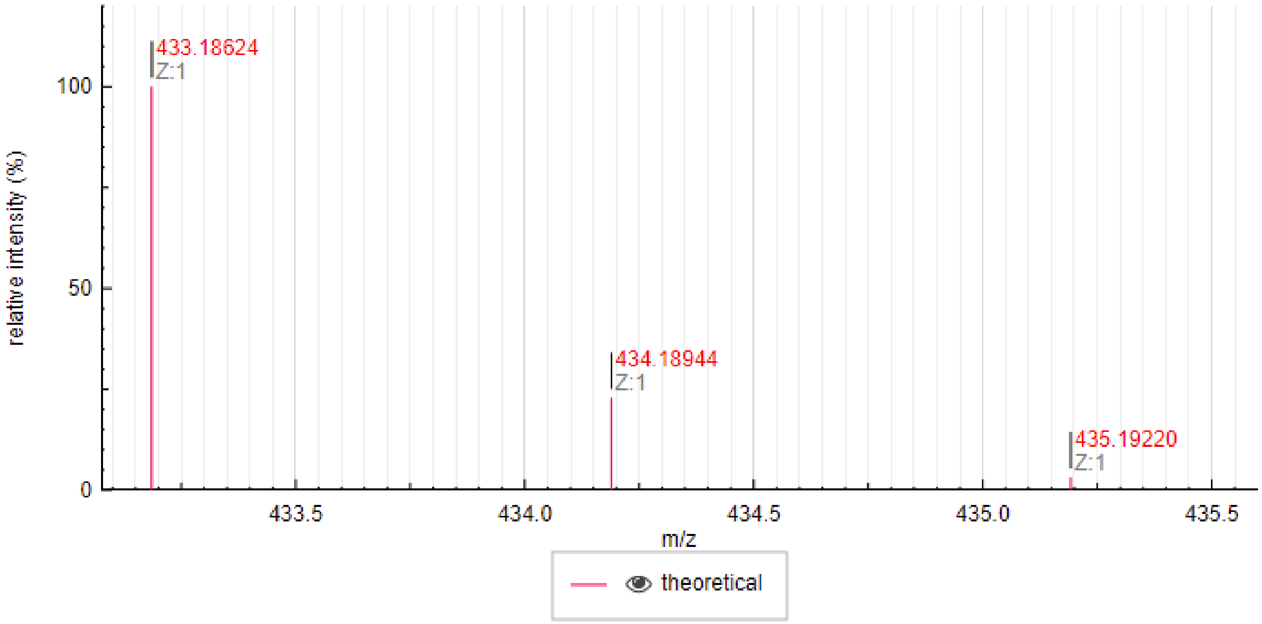

Figure S72. Theoretical HRMS $[M+H]^+$ of **4b**

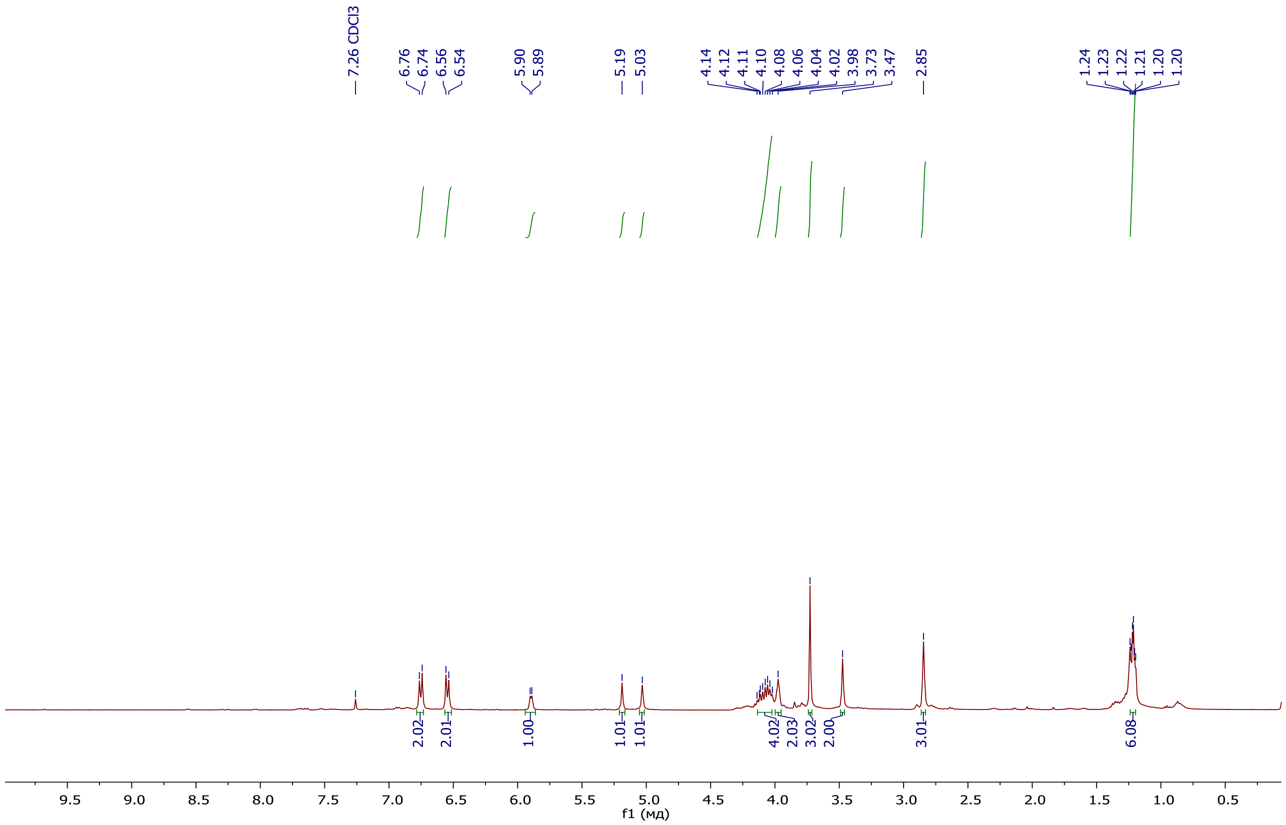

Figure S73. ^{1}H NMR of **4c** in $CDCl_3$

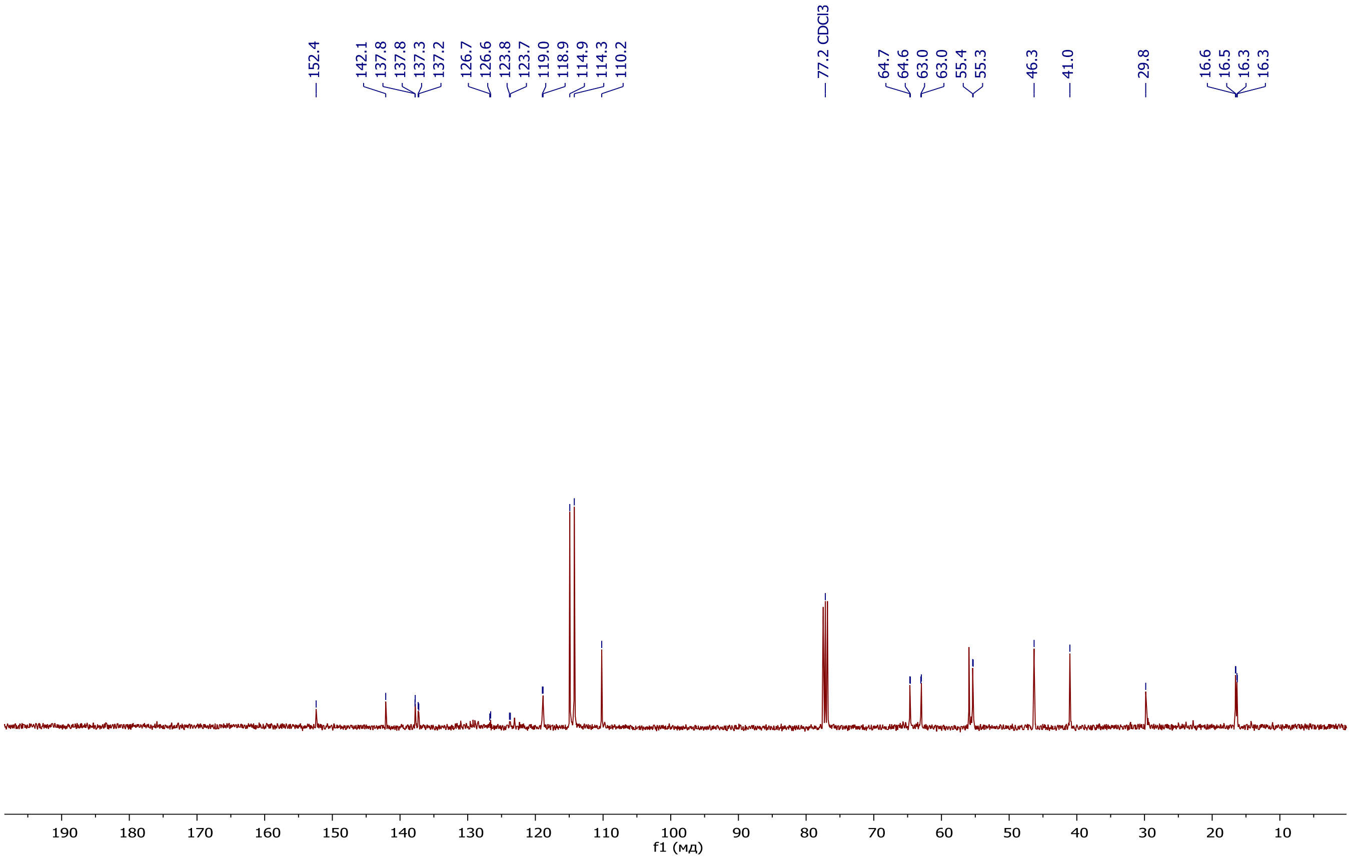

Figure S74. ^{13}C NMR of **4c** in $CDCl_3$

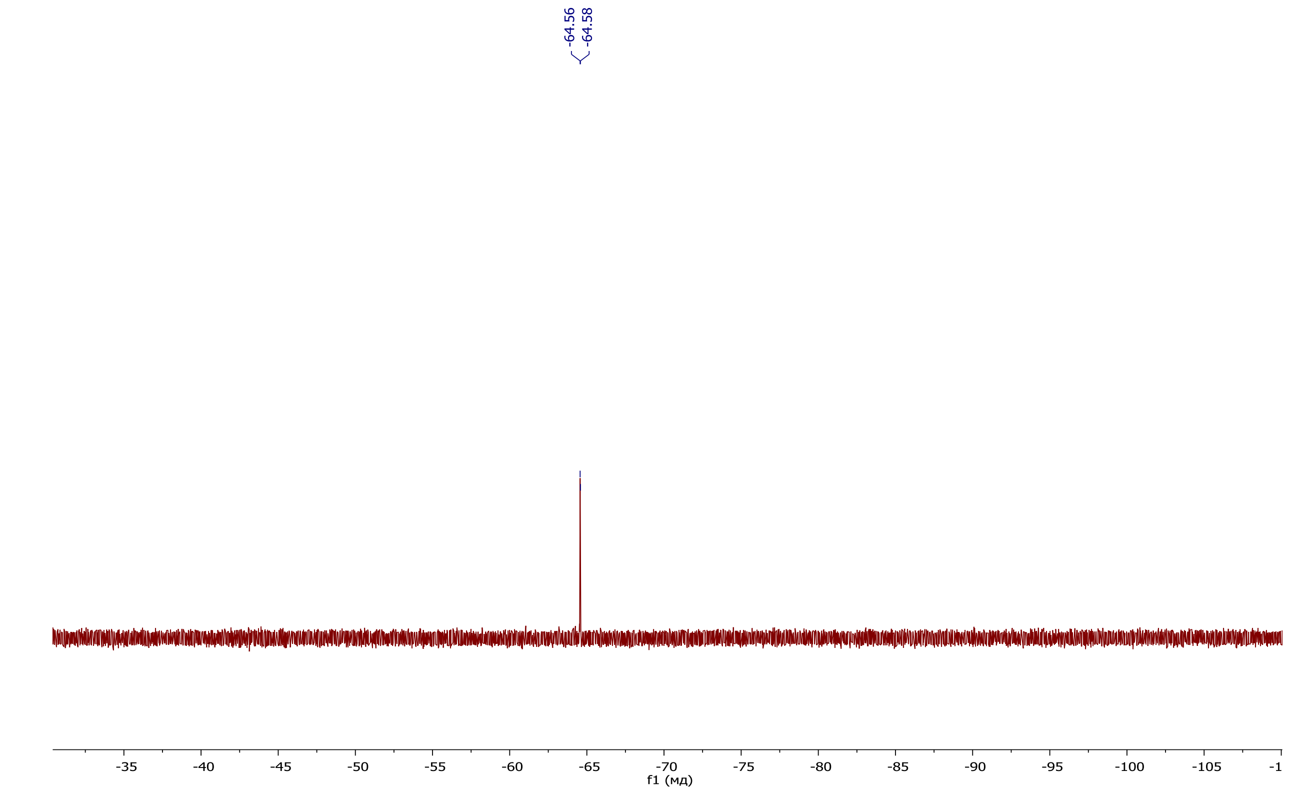

Figure S75. ^{19}F NMR of **4c** in $CDCl_3$

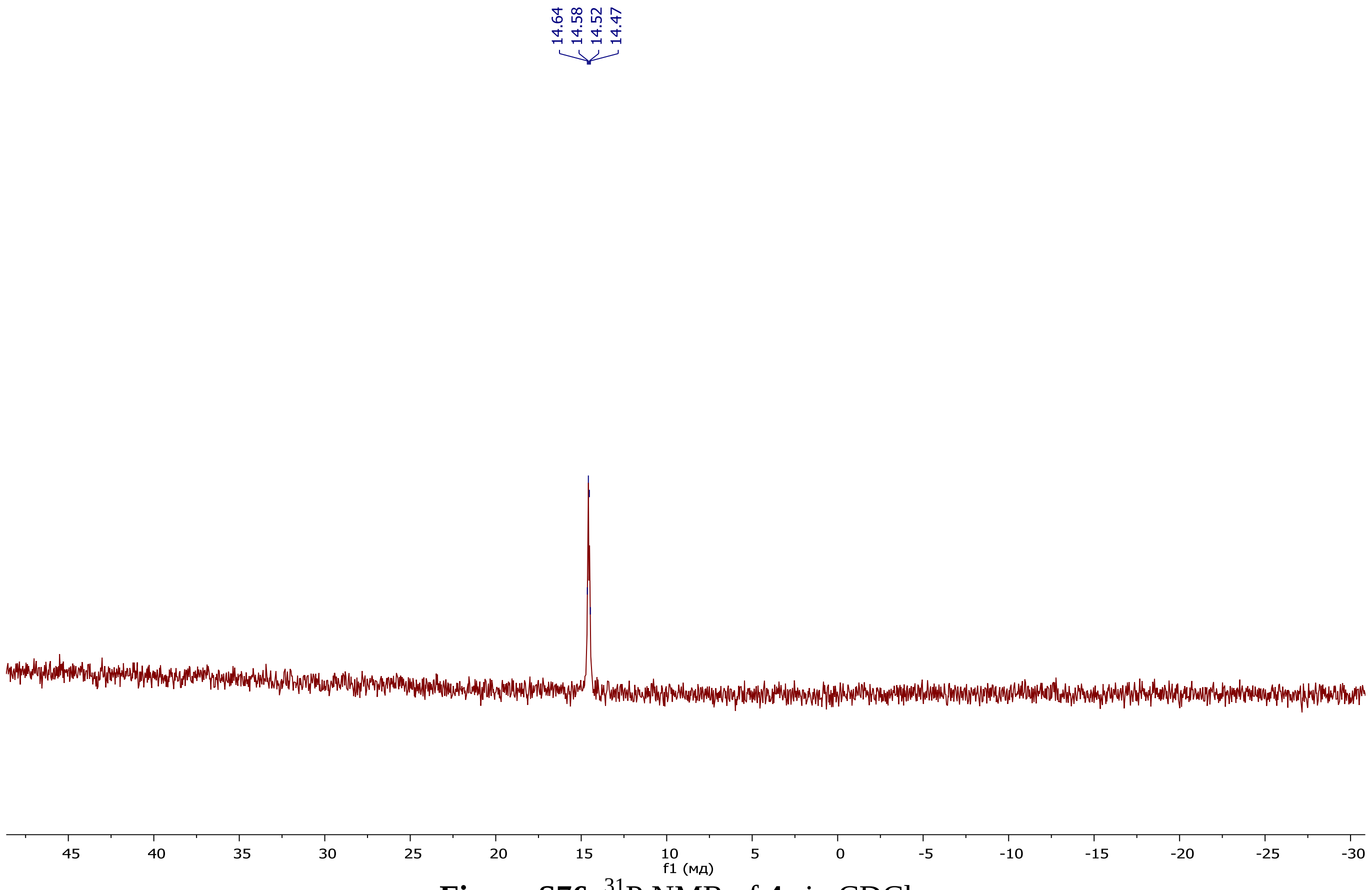

Figure S76. ^{31}P NMR of **4c** in $CDCl_3$

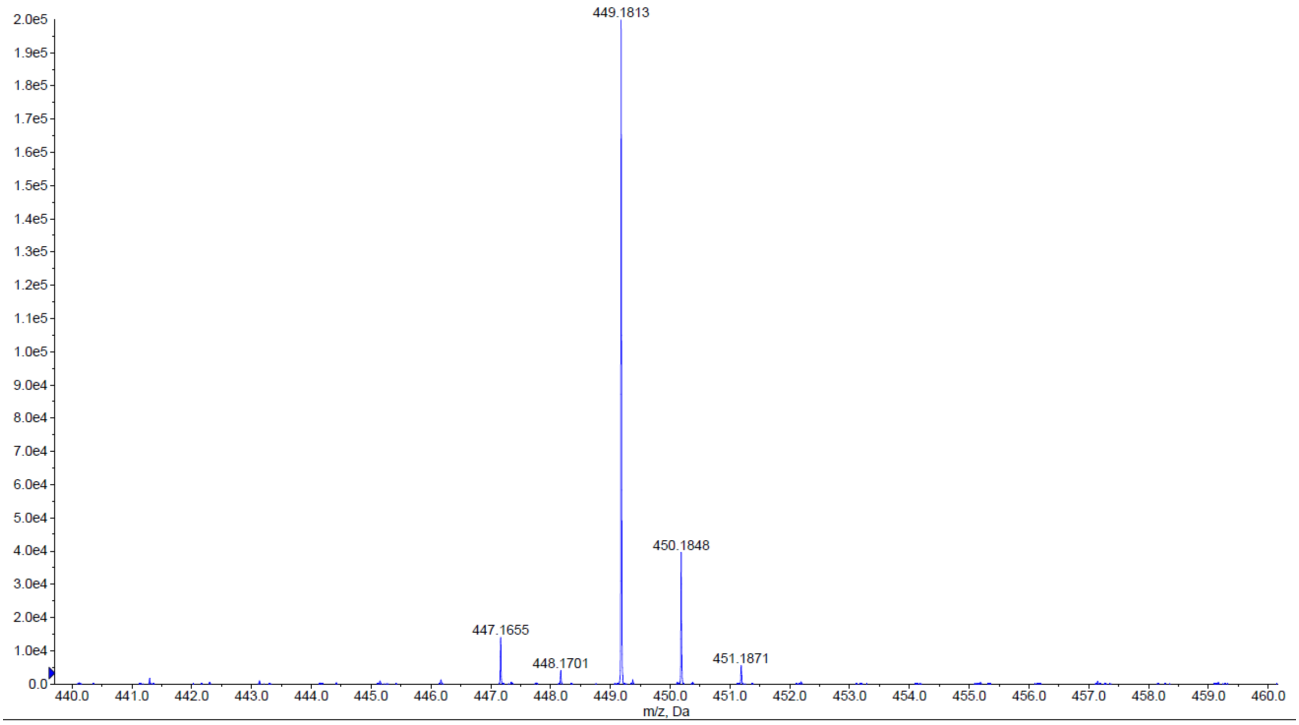

Figure S77. HRMS of **4c**

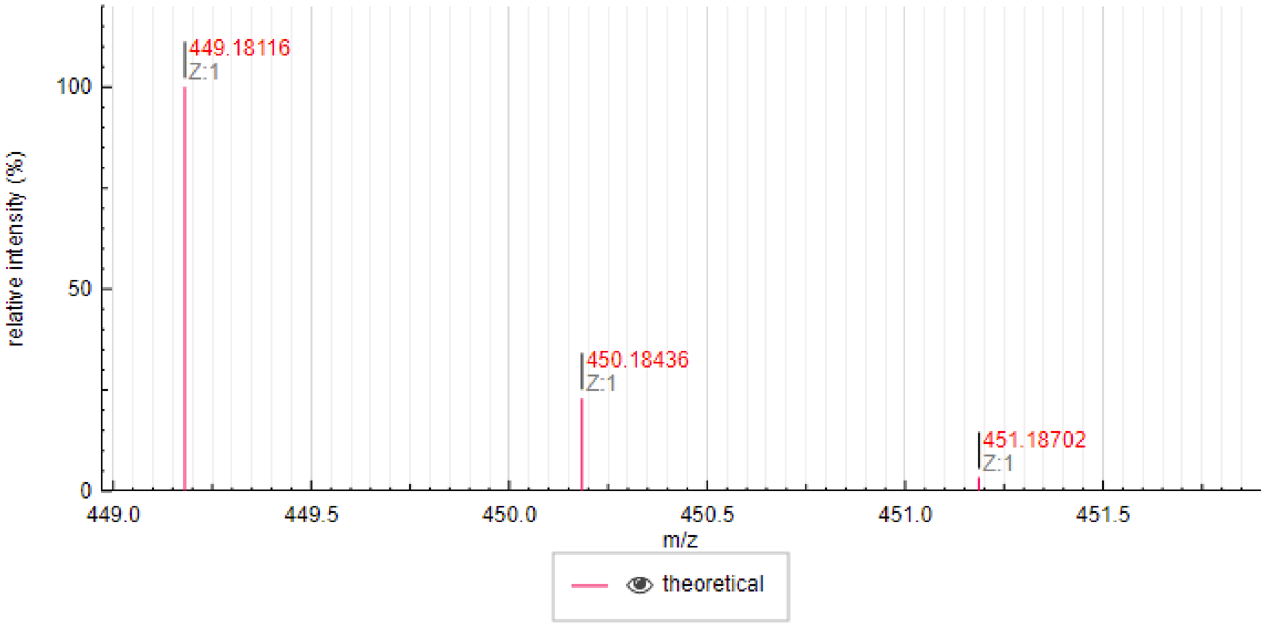

Figure S78. Theoretical HRMS $[M+H]^+$ of **4c**

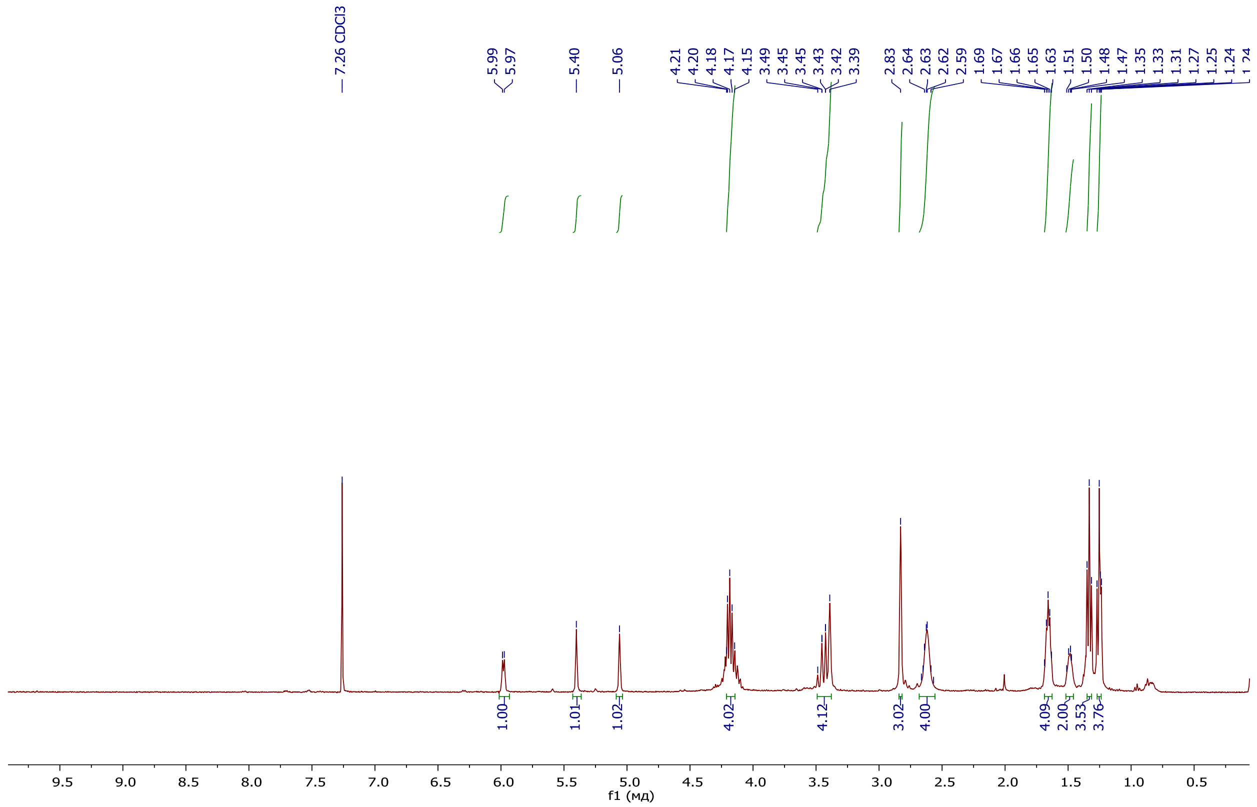

Figure S79. ^{1}H NMR of **4d** in $CDCl_3$

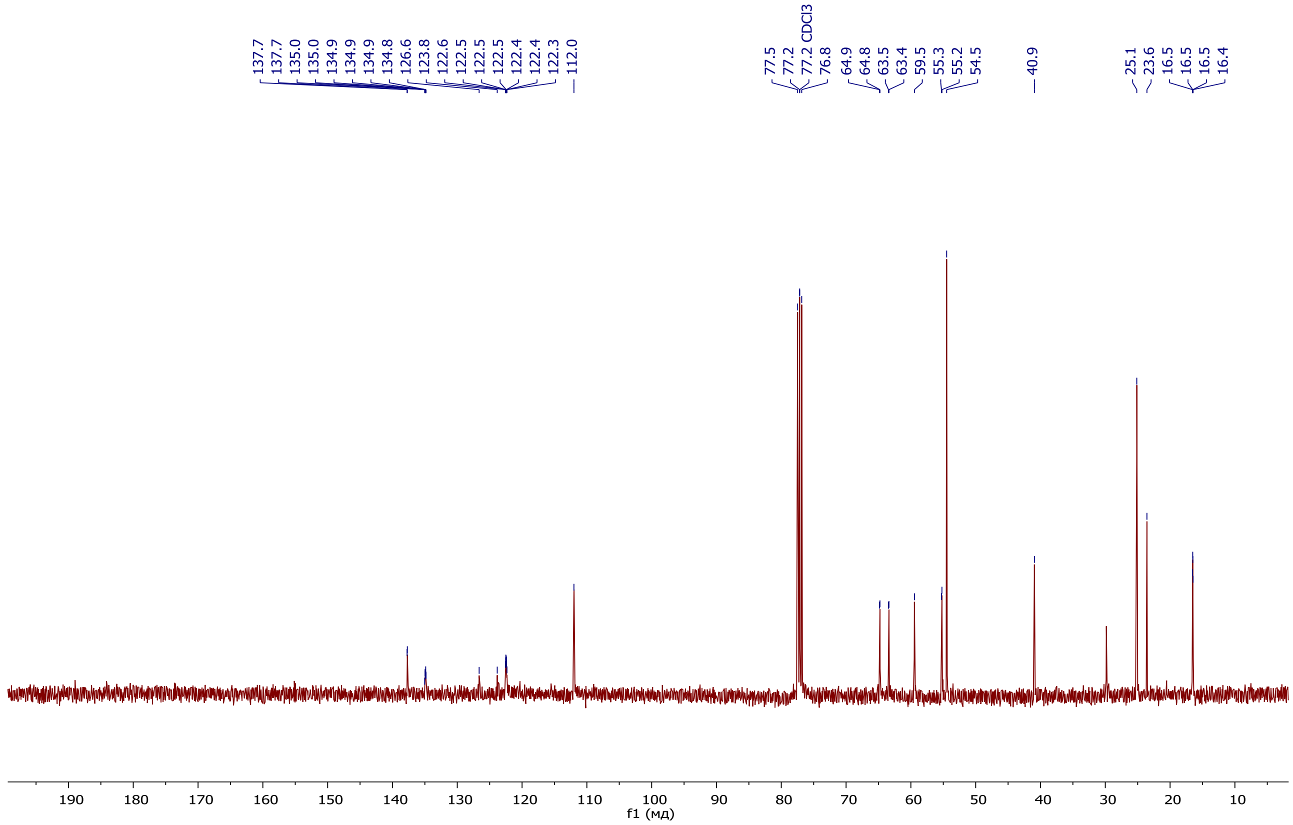

Figure S80. ^{13}C NMR of **4d** in $CDCl_3$

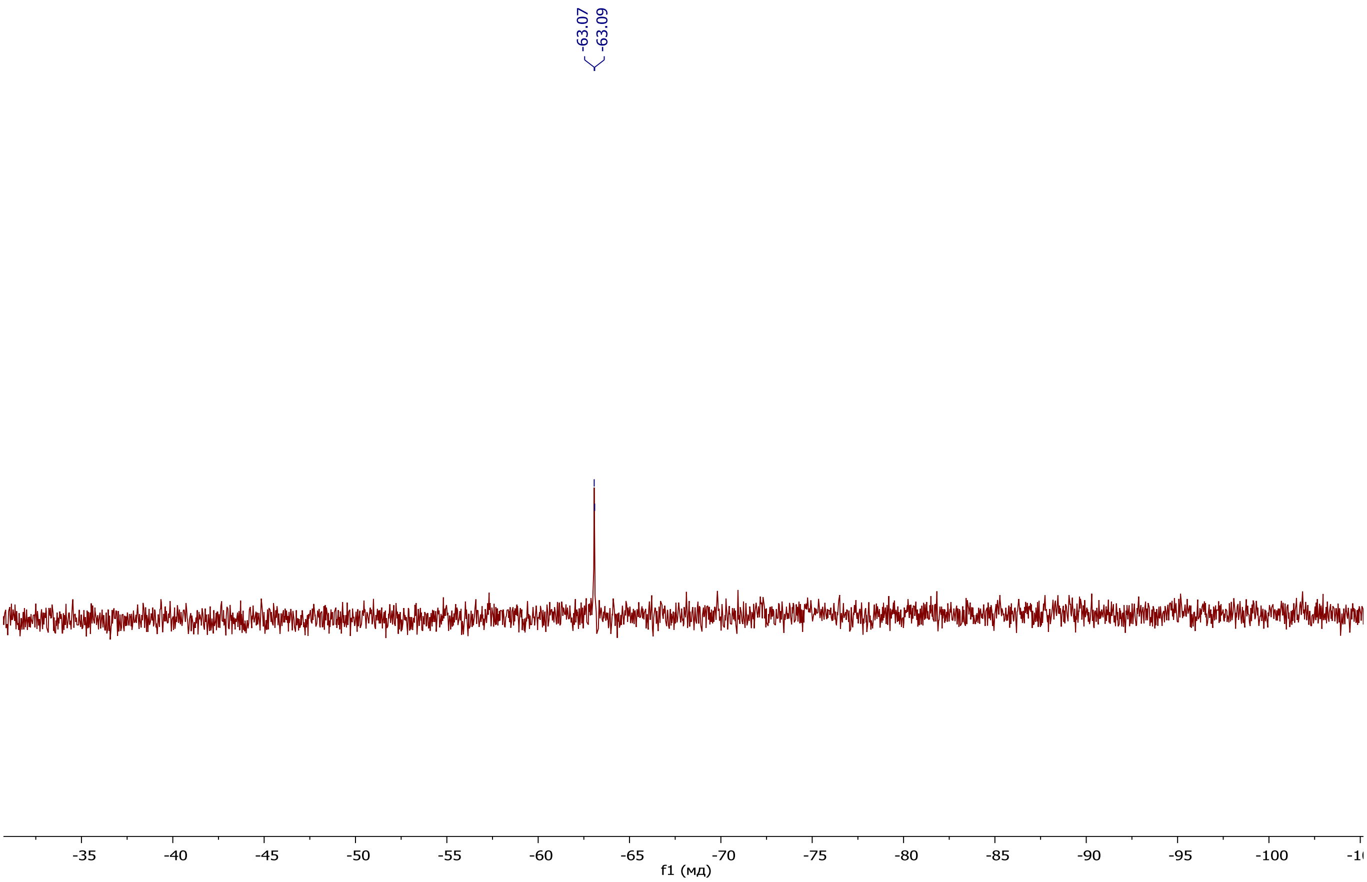

Figure S81. ^{19}F NMR of **4d** in $CDCl_3$

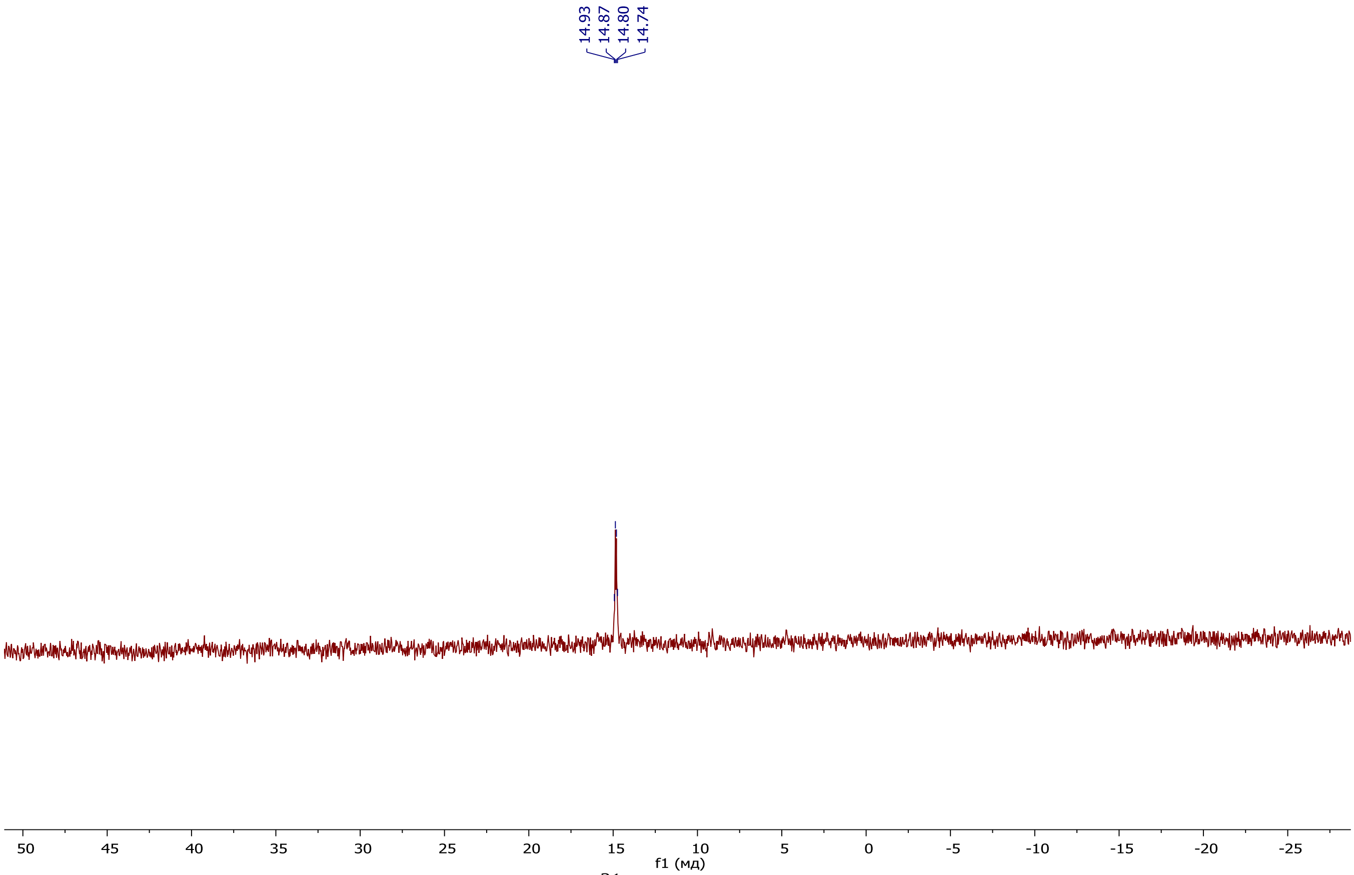

Figure S82. ^{31}P NMR of **4d** in $CDCl_3$

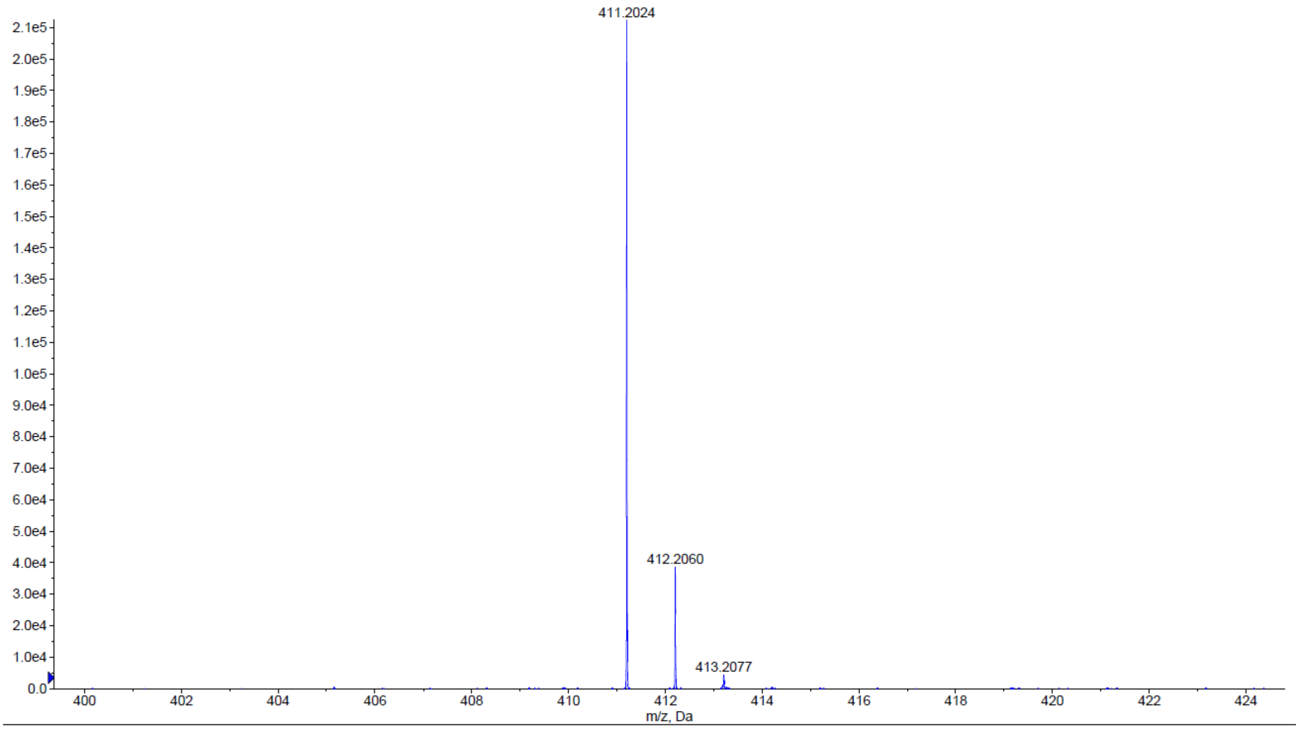

Figure S83. HRMS of **4d**

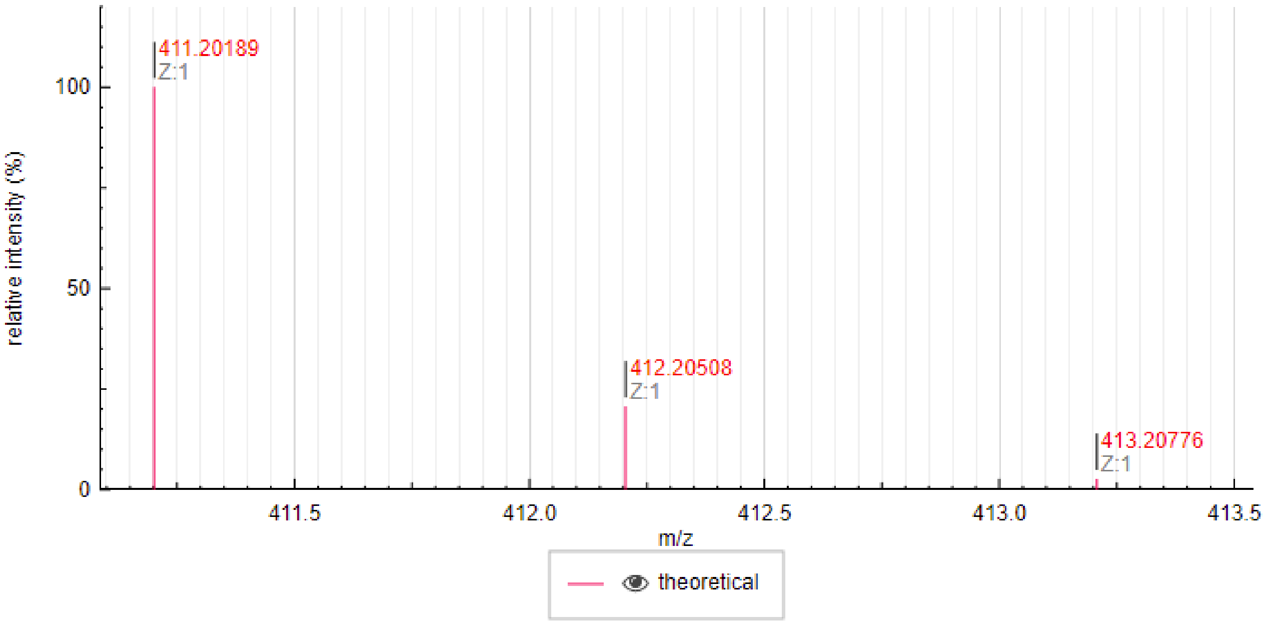

Figure S84. Theoretical HRMS $[M+H]^+$ of **4d**

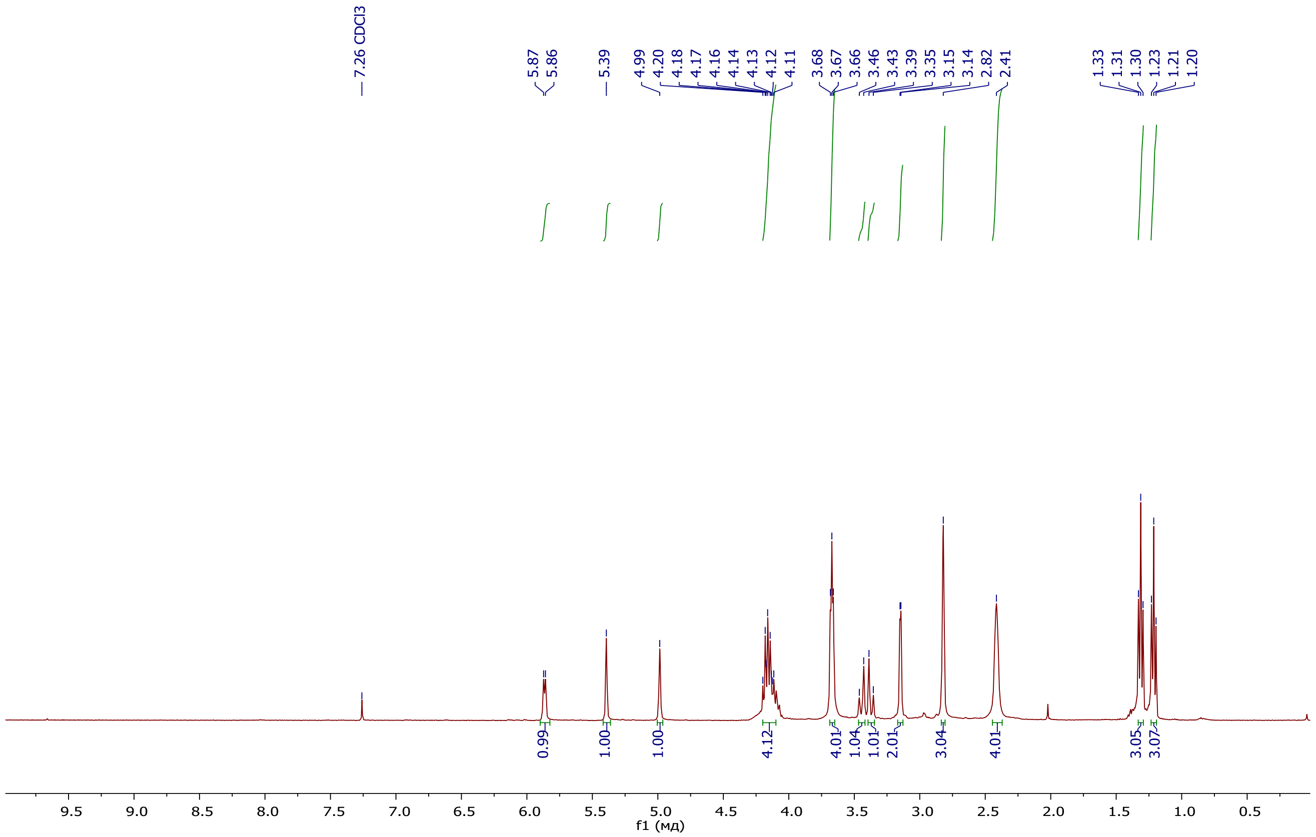

Figure S85. ^{1}H NMR of **4e** in $CDCl_3$

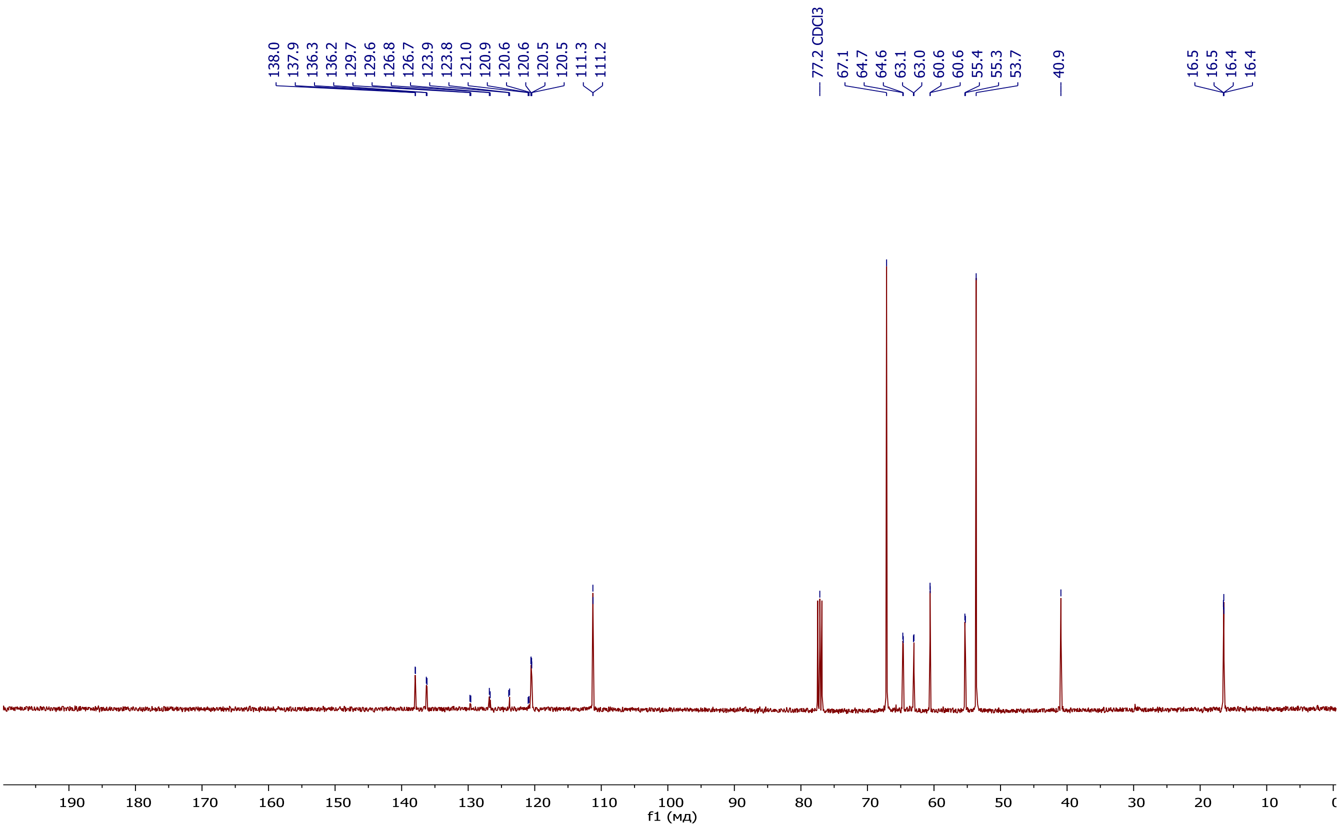

Figure S86. ^{13}C NMR of **4e** in $CDCl_3$

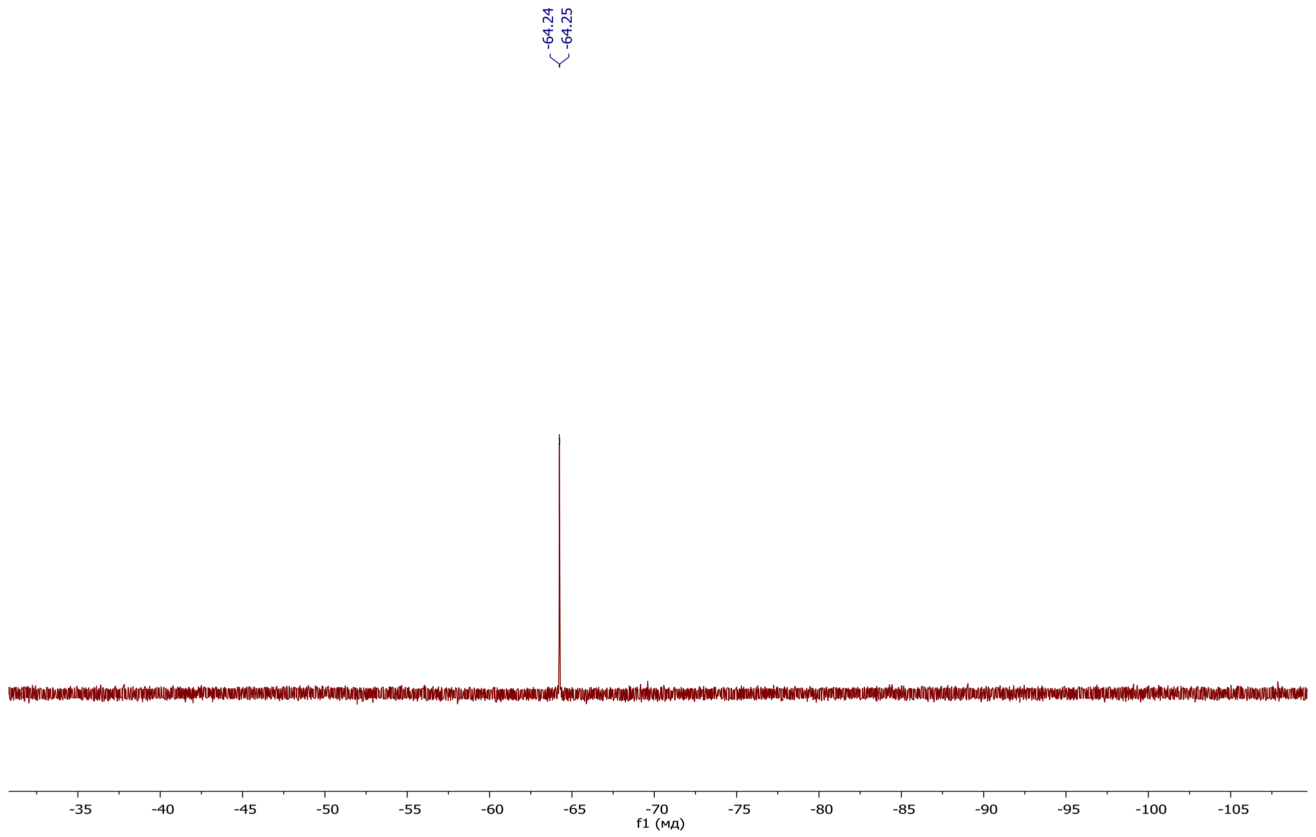

Figure S87. ^{19}F NMR of **4e** in $CDCl_3$

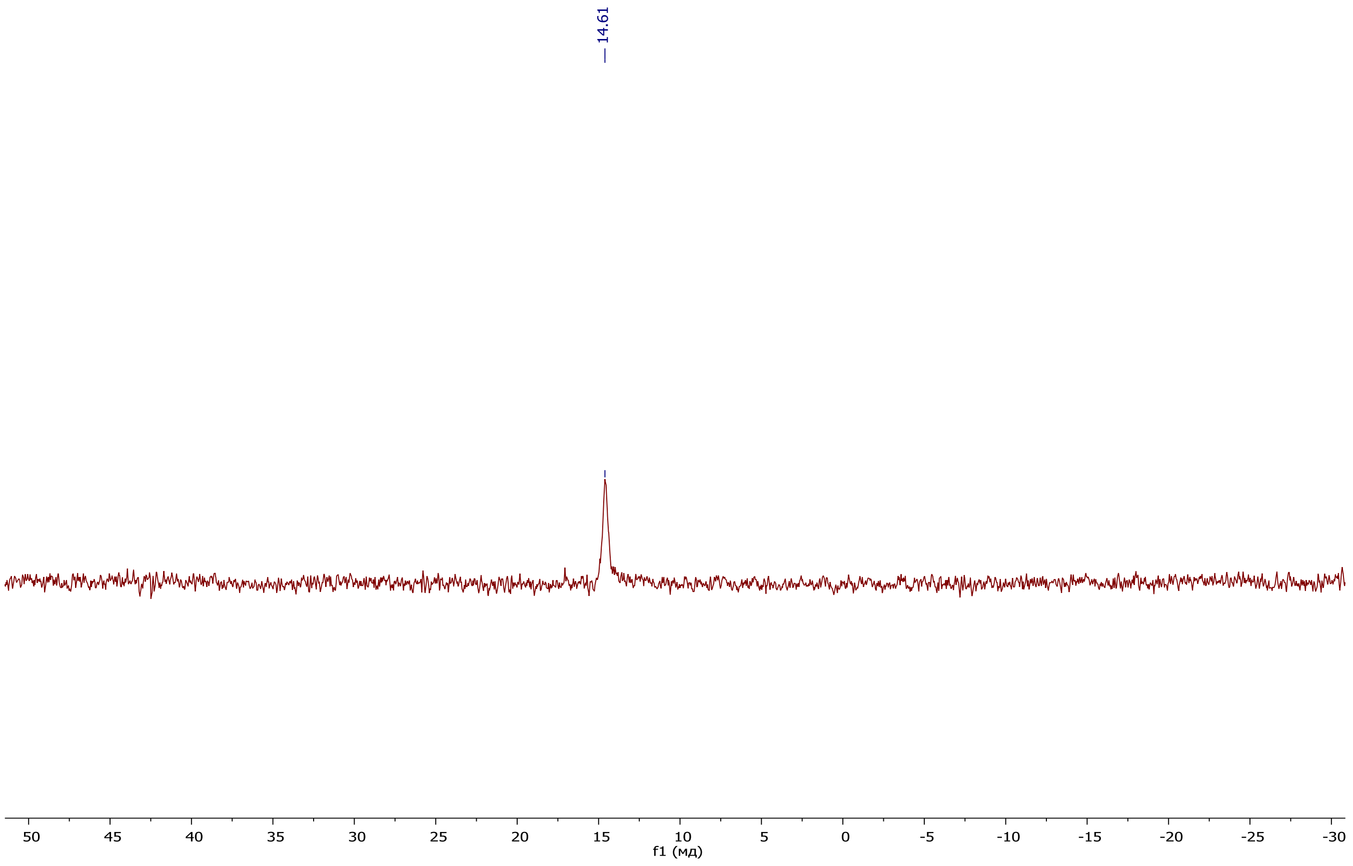

Figure S88. ^{31}P NMR of **4e** in $CDCl_3$

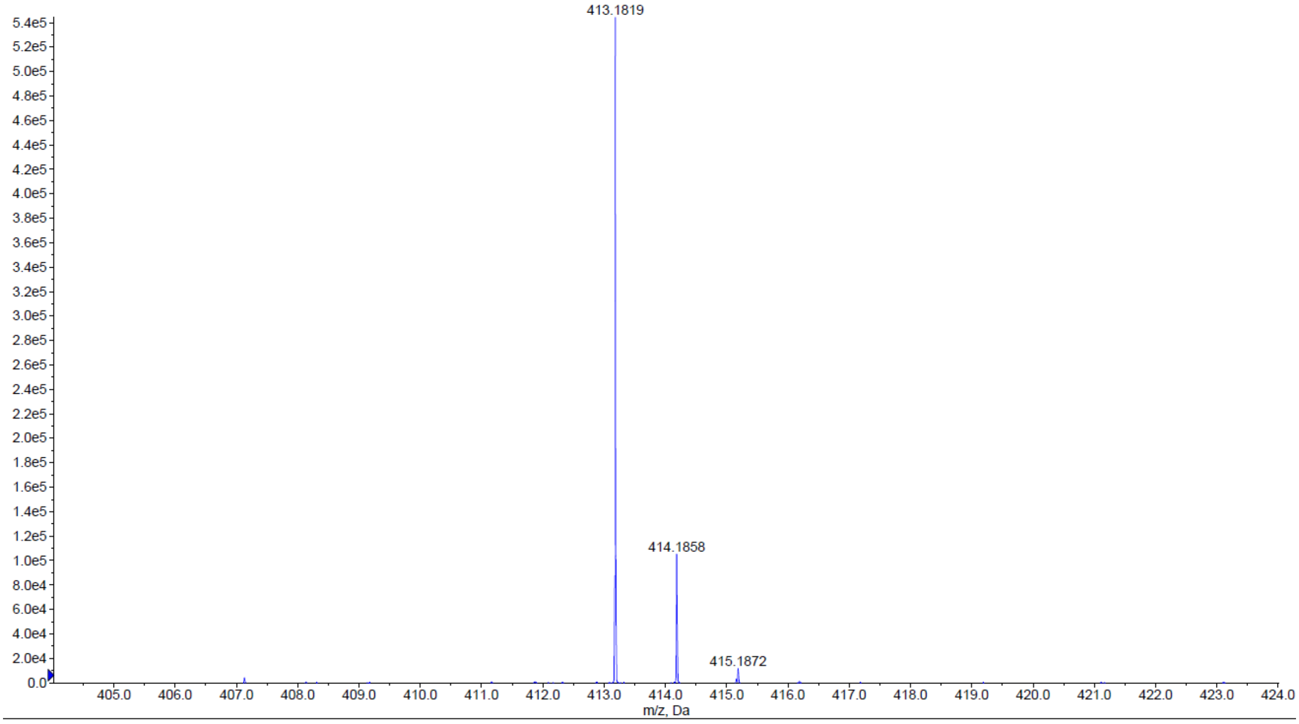

Figure S89. HRMS of **4e**

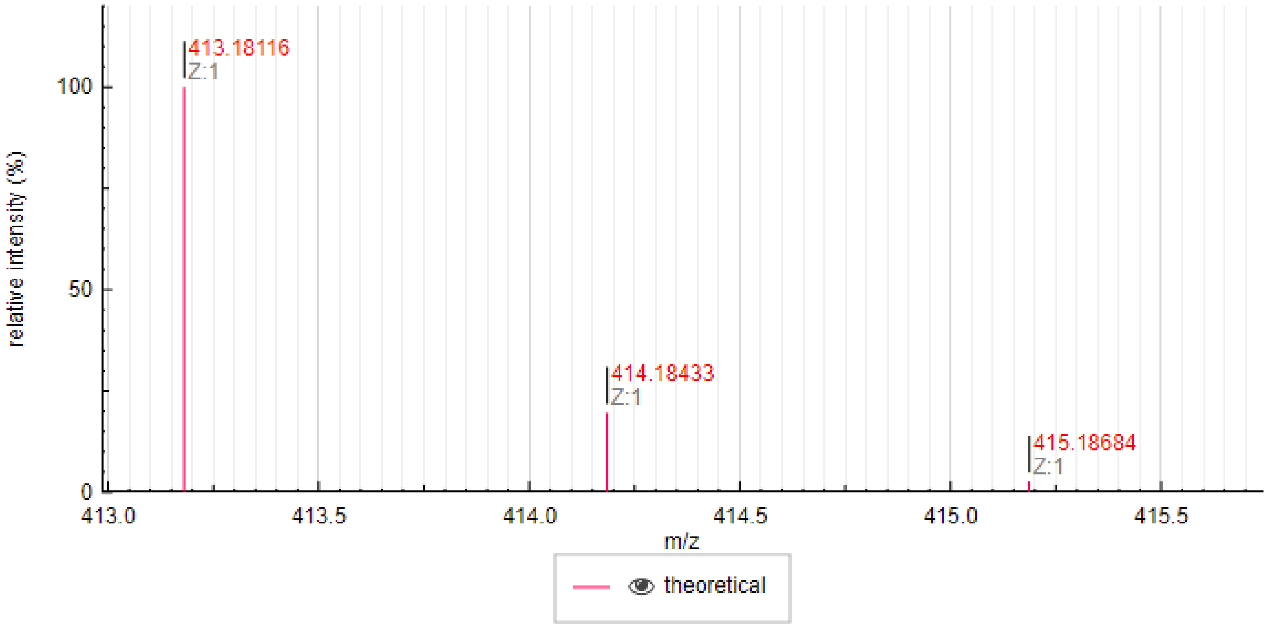

Figure S90. Theoretical HRMS $[M+H]^+$ of **4e**

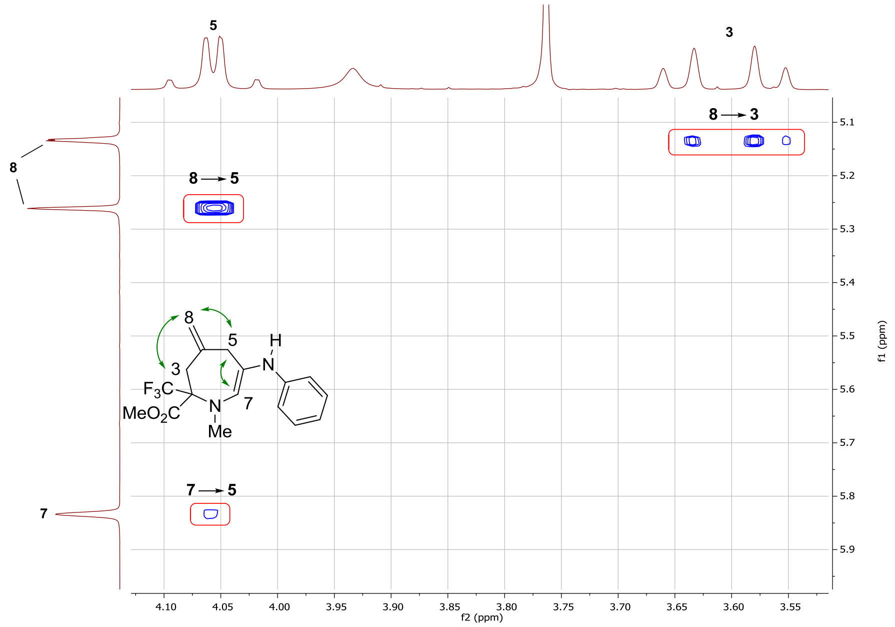

Figure S91. A fragment of 2D ^{1}H ROESY NMR spectrum (500 MHz, $CDCl_3$) of **3a**.

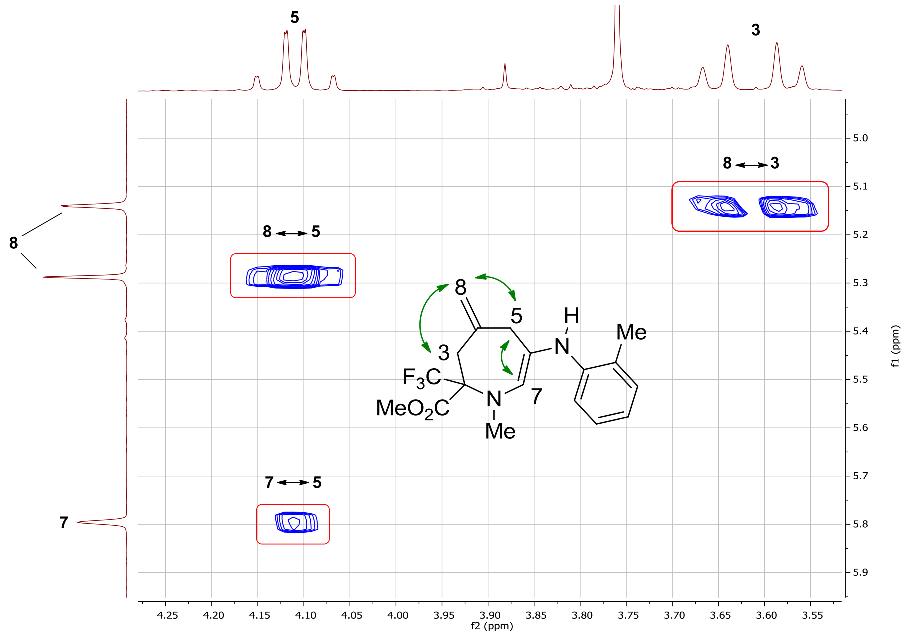

Figure S92. A fragment of 2D ^{1}H ROESY NMR spectrum (500 MHz, $CDCl_3$) of **3d**.
